# Pain Severity Trajectories and Patient Profiles in Chronic Pain Patients Treated with Cannabis‐Based Medicinal Products: A k‐Means Clustering Analysis of the UK Medical Cannabis Registry

**DOI:** 10.1002/jcph.70313

**Published:** 2026-10-16

**Authors:** Simon Erridge, Aritra Datta, Aditya Mukherjee, Dhruv Vasooja, Ross Coomber, Sushil Beri, Shaheen A. Khan, Mark W. Weatherall, Michael W. Platt, James J. Rucker, Pedro A. M. Mediano, Mikael H. Sodergren

**Affiliations:** ^1^ Medical Cannabis Research Group Department of Surgery and Cancer Imperial College London London UK; ^2^ Curaleaf Clinic Curaleaf International London UK; ^3^ St. George's Hospital London UK; ^4^ Imperial College Healthcare NHS Trust London UK; ^5^ Guy's and St. Thomas' NHS Foundation Trust London UK; ^6^ Buckinghamshire Healthcare NHS Trust Amersham UK; ^7^ Department of Psychological Medicine King's College London London UK; ^8^ South London and Maudsley NHS Foundation Trust London UK; ^9^ Department of Computing Imperial College London London UK

**Keywords:** anxiety, cannabis, cannabidiol, pain, sleep, tetrahydrocannabinol

## Abstract

Cannabis‐based medicinal products (CBMPs) are increasingly prescribed for chronic pain, yet the supporting evidence remains limited and the extent to which patients differ in their response is poorly understood. This study applied longitudinal k‐means clustering to pain‐severity trajectories in patients treated with CBMPs for chronic pain to distinguish patterns of response and identify the baseline characteristics predicting them over 24 months. A cohort study was undertaken of UK Medical Cannabis Registry participants whose primary indication was chronic pain. Pain, health‐related quality of life, anxiety, and sleep were assessed with patient‐reported outcome measures, including the Brief Pain Inventory Short Form (BPI‐SF), EuroQol 5‐Dimension 5‐Level (EQ‐5D‐5L), Generalised Anxiety Disorder‐7, and Single‐Item Sleep Quality Scale, at baseline up to 24 months. BPI‐SF pain‐severity trajectories were grouped by longitudinal k‐means clustering. The number of clusters was chosen using the gap statistic and predictors of cluster membership were examined by logistic regression. Of 3744 patients included, every outcome improved from baseline to 24 months, including BPI‐SF pain severity (d = 0.57), pain interference (d = 0.59), and the EQ‐5D‐5L index value (d = −0.76; all *P* <  .001). More than half of patients achieved a minimal clinically important difference in each pain measure at 24 months. Five distinct trajectory clusters emerged. All ended below their baseline severity, but the magnitude and pattern of change varied markedly. Adverse events were reported by 14.37% (n = 538) of patients, predominantly mild or moderate. Baseline sleep quality, anxiety, and treatment indication were more strongly associated with response than product‐ or dose‐related factors.

## Introduction

Chronic pain is defined as pain that persists or recurs for longer than 3 months.^1^ It is among the most prevalent and disabling conditions, affecting more than 30% of people globally and among the leading contributors to years lived with disability.^2^ In the United Kingdom, a meta‐analysis of 19 population studies estimated the pooled prevalence of chronic pain at 43.5%, equivalent to just under 28 million adults, of whom 10.4%–14.3% experience moderately‐to‐severely disabling pain.^3^ Beyond its physical manifestations, chronic pain is bidirectionally associated with psychological distress and sleep disturbance, contributing to further reduction in health‐related quality of life.^2^ Its economic consequences are commensurately large. In the United States, the total burden attributable to pain has been estimated at $560–635 billion USD annually, comprising $261–300 billion USD in additional healthcare costs and $299–335 billion USD in lost productivity, exceeding the respective costs of heart disease, cancer, and diabetes.^4^ Moreover, chronic pain remission is uncommon. In the HUNT pain study, individuals with moderate‐to‐severe chronic pain had only an 8% probability of recovery over 1 year.^5^ Among those who did recover, just 52% had sustained recovery at the subsequent follow‐up, with the prognosis worsening the longer pain persisted.^5^

The principal cannabinoids within cannabis‐based medicinal products (CBMPs), (−)‐*trans*‐Δ^9^‐tetrahydrocannabinol (THC) and cannabidiol (CBD), interact with molecular targets at each level of the pain neuraxis.^6^ THC is a partial agonist at the cannabinoid CB1 and CB2 receptors. Peripherally, CB1 receptors expressed on primary afferent nociceptors and dorsal root ganglion neurons, together with CB2 receptors on immune cells, reduce nociceptor excitability and the release of pro‐inflammatory mediators that drive peripheral sensitization.^7^ At the spinal level, CB1 receptors within the superficial dorsal horn attenuate excitatory glutamatergic neurotransmission and thereby limit the central sensitization that underlies chronic pain.^8^ Supraspinally, CB1 receptors in the periaqueductal grey and rostral ventromedial medulla engage descending inhibitory control of nociceptive transmission, whilst CB1‐mediated signaling within the amygdala, anterior cingulate, and medial prefrontal cortex modulates the affective and cognitive dimensions of the pain experience.^8^ CBD, by contrast, has low affinity for CB1 and CB2 receptors and instead exerts analgesic and anxiolytic effects predominantly through transient receptor potential vanilloid‐1 (TRPV1) and 5‐hydroxytryptamine 1A (5‐HT1A) receptors.^9^ Both cannabinoids produce dose‐dependent antinociception in inflammatory, osteoarthritic, and neuropathic models of chronic pain.^9^, ^10^, ^11^, ^12^, ^13^, ^14^, ^15^ In humans, however, their clinical pharmacology is characterized by marked inter‐individual variability. The oral bioavailability of THC is low and erratic, at approximately 4%–12%, compared with 10%–35% following inhalation.^16^ Both THC and CBD undergo extensive hepatic metabolism by cytochrome P450 enzymes, creating the potential for pharmacokinetic interactions with concomitant medicines.^16^, ^17^ Tolerance also develops to several effects of THC with repeated exposure, to a degree that varies with prior cannabis use.^18^ These properties suggest that the analgesic response to CBMPs is unlikely to be uniform across patients.

Notwithstanding the mechanistic rationale, the clinical evidence base remains limited, with recent systematic reviews reaching different conclusions according to the inclusion and exclusion criteria applied. In a meta‐analysis of 32 randomized trials, Wang and colleagues found that non‐inhaled medical cannabis confers a 10% risk difference in the likelihood of patients achieving the minimally important difference in pain relief, alongside small improvements in physical functioning and sleep quality.^19^ Reviewing 36 trials over a similar period, Fisher and colleagues identified evidence of benefit only for cannabis given for under 7 days and for nabiximols beyond 7 days (risk difference 0.06, 95% CI 0.01–0.12).^20^ With 81% of subgroup analyses negative, the authors concluded that the evidence neither supported nor refuted efficacy for CBMPs in pain.^20^ Barakji and colleagues, synthesizing 65 trials, reported that cannabinoids reduced chronic pain, but the margin of improvement was below their pre‐specified minimally important difference, and the authors judged the harms to outweigh the benefits.^21^ A recurring conclusion across these syntheses is therefore that, where an analgesic effect of medical cannabis is detectable, its magnitude is consistently negligible to small.^19^, ^20^, ^21^ Notably, when methodologically rigorous trials are undertaken, clinically and statistically significant benefits emerge.^22^, ^23^ In a Phase 3, placebo‐controlled trial of the full‐spectrum extract VER‐01 in 820 adults with chronic low back pain, mean pain intensity fell by 1.9 points on an 11‐point scale with a mean difference of −0.6 compared to placebo.^23^ This reduction increased to 2.9 points during open‐label extension, whilst a separate Phase 3 trial found VER‐01 superior to opioids for both pain reduction and sleep quality with a 4‐fold lower risk of constipation.^22^, ^23^ The small average effects reported across randomized trials may conceal heterogeneous individual responses, in which some patients derive substantial benefit and others little or none.^24^ Analyses based on mean change cannot distinguish these patterns. An earlier longitudinal k‐means clustering analysis of health‐related quality of life across all indications in the UK Medical Cannabis Registry identified distinct response trajectories, including subgroups that did not improve.^25^ Whether comparable heterogeneity exists in pain severity among patients treated for chronic pain has not been examined. Against this background, the present study aims to report the outcomes of a cohort of patients treated with CBMPs for chronic pain in the United Kingdom, to identify distinct trajectories of pain severity over 24 months using longitudinal k‐means clustering, and to determine the patient and treatment characteristics associated with each trajectory. Such phenotypes may inform patient selection and stratification in future randomized trials, as well as the development of licensed therapies, clinical guidelines, and patient care.

## Methods

### Study Design, Setting, and Participants

Established in December 2019, the UK Medical Cannabis Registry is a prospective database that captures clinical outcomes for patients across the United Kingdom and Crown Dependencies who are prescribed CBMPs through Curaleaf Clinic.^26^ The registry holds a favorable ethical opinion from the Central Bristol Research Ethics Committee (22/SW/0145). Enrolment is consecutive, written informed consent is obtained beforehand, and data collection and analysis adhere to both the Declaration of Helsinki and Good Clinical Practice. For the present cohort, study eligibility was restricted to registry participants whose primary treatment indication was chronic pain and who received unlicensed CBMPs produced to Good Manufacturing Practice standards, initiated at the discretion of a clinician on the General Medical Council Specialist Register and overseen by a multidisciplinary team.^27^, ^28^ Individuals were ineligible if no baseline patient‐reported outcome measures (PROMs) had been completed, or if they had been enrolled for less than 2 years at the point of data extraction (January 6, 2025). Reporting follows the STrengthening the Reporting of OBservational studies in Epidemiology (STROBE) statement.^29^

### Data Collection

At the baseline consultation, clinicians documented demographic information, including age, sex, body mass index (BMI), and occupation coded to the International Standard Classification of Occupations.^30^ Medical history; comorbidities and the Charlson Comorbidity Index^31^; drug history; and the primary, secondary, and tertiary indications for treatment were also documented. Tobacco and alcohol consumption, and pre‐treatment cannabis status, categorized as cannabis naïve, former consumer, or current consumer, were also recorded, and all participants were advised to stop other sources of cannabis while under treatment. Concurrent medications were coded using the Systematized Nomenclature of Medicine.^32^ Opioid doses were expressed as the oral morphine equivalent (OME) in mg/day using conversion values from the British National Formulary, and the minimal clinically important difference (MCID) for OME reduction was set at a fall of at least 28.2% from baseline.^33^, ^34^ Gabapentinoid doses were expressed as a daily pregabalin‐equivalent dose in mg/day.^35^

For each active CBMP prescription, the formulation, administration route, and dose were used to derive daily CBD and THC exposure, taking the midpoint of the permitted daily dosing range where a range was specified.

PROMs were completed remotely at baseline and at 1, 3, 6, 12, 18, and 24 months, with automated email reminders sent every 72 h until a response was received.^26^ Pain was captured with the Brief Pain Inventory Short Form (BPI‐SF), which provides distinct pain severity and pain interference scores, together with the Short‐Form McGill Pain Questionnaire‐2 (SF‐MPQ‐2) and a pain visual analogue scale (VAS).^2^, ^36^, ^37^, ^38^, ^39^

The BPI‑SF is an instrument that quantifies pain severity and the interference of pain with daily function.^36^ Pain severity is derived from four 0–10 numerical rating items and pain interference from seven 0–10 items spanning general activity, mood, walking, work, relationships, sleep, and enjoyment of life, each averaged to yield a severity score and an interference score from 0 to 10.^36^ Although originally developed for cancer pain, its reliability and validity have been confirmed in chronic non‑malignant pain, with good internal consistency (Cronbach's α = 0.85 for the severity items and 0.88 for the interference items).^40^, ^41^

The SF‑MPQ‑2 characterizes the sensory and affective qualities of both neuropathic and non‑neuropathic pain across 22 descriptors, each rated on a 0–10 numerical rating scale from none to worst possible.^38^, ^42^ It yields a total score and four subscales (continuous pain, intermittent pain, neuropathic pain, and affective descriptors), each expressed as the mean of its constituent items from 0 to 10.^38^, ^42^ Developed and validated across diverse chronic pain syndromes and in painful diabetic peripheral neuropathy, it has demonstrated excellent reliability and validity, with both exploratory and confirmatory analyses supporting its four‑factor structure.^38^, ^42^

The pain VAS records overall pain intensity on a continuous line anchored between no pain and worst imaginable pain, scored from 0 to 10.^39^ It is quick to complete, correlates closely with other pain‑intensity measures, and has good test–retest reliability (r > 0.71).^39^

Health‐related quality of life and related domains were measured with the EuroQol 5‐Dimension 5‐Level (EQ‐5D‐5L), from which an index value was calculated using the UK value set favored by the National Institute for Health and Care Excellence for health technology assessment.^43^, ^44^, ^45^ The Generalised Anxiety Disorder‐7 (GAD‐7), the Single‐Item Sleep Quality Scale (SQS), and the Patient Global Impression of Change (PGIC) were additionally administered.^46^, ^47^, ^48^ Full details on scoring and psychometric properties are detailed in a previous paper by our group.^25^

For each pain‐specific PROM, three thresholds of improvement from baseline were pre‐specified. An MCID of a reduction of at least 1 point, a moderately important improvement (MII) of at least a 30% reduction, and a substantially important improvement (SII) of at least a 50% reduction.^37^ As no established MCID exists for the EQ‐5D‐5L index value, any positive improvement from baseline was used, whereas the MCID was defined as an improvement of at least 4 points for the GAD‐7 and at least 2.6 points for the SQS.^47^, ^49^

Adverse events were recorded as they arose, whether during PROM completion, at the time of occurrence, or at clinical review, and were graded with the Common Terminology Criteria for Adverse Events, version 4.0.^50^

### Missing Data

The extent and pattern of missing data were assessed and handled according to the assumed mechanism of missingness. Baseline demographic and comorbidity data were handled by listwise deletion, being assumed missing completely at random and constituting a small proportion of the dataset. The absence of an adverse event or concurrent medication record was taken to indicate that no event had occurred, no medication had been prescribed, or dosing had remained unchanged. Missing PROM data were addressed by multiple imputation using the *mice* package in R, generating five imputed datasets from an imputation model incorporating all PROM variables across timepoints to preserve the longitudinal correlation structure with efficiency ≥90%.^51^, ^52^ Data were assumed to be missing at random conditional on the observed PROM values. This approach was utilized to develop a consistent dataset for all subsequent analyses on PROMs, rather than utilizing different methods to address missingness across analyses. The number of observed responses for each PROM at each timepoint was reported. To assess the potential for attrition bias, baseline characteristics were compared between participants with and without an observed BPI‐SF pain‐severity score at 24 months using Welch's t‐test or the χ^2^ test. The change in BPI‐SF pain severity from baseline to 24 months was additionally repeated as a complete‐case sensitivity analysis.

### Statistical Analysis

Descriptive statistics were presented as count (%), mean ± standard deviation (SD), median [interquartile range (IQR)], or odds ratio (OR) with 95% confidence interval (CI). Parametric approaches were applied throughout, justified by the central limit theorem given the large sample.^53^ Change across the seven timepoints in each PROM, OME dose, and pregabalin‐equivalent dose was evaluated by repeated‐measures analysis of variance. Mauchly's test was used to assess sphericity and the Greenhouse–Geisser correction applied where it was violated.^54^ Pairwise post hoc testing was carried out with paired t‐tests. The family‐wise error rate was controlled using the Bonferroni–Holm method. Effect sizes were reported as Cohen's d and interpreted against the conventional small (0.2), medium (0.5), and large (0.8) benchmarks.^55^

For each outcome, a binary variable indicating whether the relevant improvement threshold had been met was derived. Univariable and multivariable logistic regression were then used to evaluate predictors of clinically significant improvement at 24 months in the EQ‐5D‐5L index value, GAD‐7, SQS, BPI‐SF pain severity, BPI‐SF pain interference, SF‐MPQ‐2 total score, and pain VAS, as well as of adverse‐event occurrence. The candidate predictors spanned demographic characteristics (age category, sex, and BMI category), substance use history (cannabis and smoking status), clinical variables (primary diagnosis category, baseline anxiety and sleep scores, and concomitant medication), and treatment‐related factors (CBMP formulation and CBD and THC dosing).

Distinct pain‐severity trajectories were identified by applying longitudinal k‐means clustering to the BPI‐SF pain‐severity scores over the seven timepoints using the *kml* package.^56^ Clustering was performed on the mean of the five imputed values at each timepoint. The number of clusters was chosen by the gap statistic estimated from 1000 bootstrap samples. Assignments were obtained from 100 random starts, each optimized by the standard k‐means algorithm. The solution with the highest silhouette score was retained. Each cluster was then contrasted with the remainder combined. Associations with baseline characteristics were tested by univariable logistic regression with Benjamini–Hochberg adjustment.

Cluster membership was additionally modeled by multivariable logistic regression. This used both a stepwise procedure guided by a modified Akaike Information Criterion (penalty k = 5) with class weighting to accommodate imbalance and a least absolute shrinkage and selection operator (LASSO)‐regularized model fitted with the *glmnet* package.^57^ The penalty parameter was chosen by 5‐fold cross‐validation under the one‐standard‐error rule. Discrimination was quantified by the area under the curve (AUC). All analyses were conducted in R (version 4.4.3; R Core Team, Vienna, Austria) within RStudio (Version 2024.04.2+764; Posit Software, MA). Figures were generated with *ggplot2*.^58^ Statistical significance was set at *P* <  .050.

## Results

Of the 34,563 patients enrolled in the UK Medical Cannabis Registry at the data‐extraction date of January 6, 2025, those with no complete baseline PROMs (n = 3055; 8.84%) and patients enrolled <2 years prior to data extraction (n = 22,563; 71.61%) were excluded from analysis. Patients without chronic pain were removed from analysis (n = 5201; 58.14%). Therefore, 3744 met the inclusion criteria for the present analysis.

The mean age of the cohort was 46.36 ± 13.92 years, and 2172 (58.01%) were male (Table 1). Most participants were current (n = 2152; 57.48%) or former (n = 590; 15.76%) cannabis consumers prior to commencing CBMPs, with a median lifetime cannabis exposure of 8.00 [2.00–20.00] gram years. Their geographic distribution across the United Kingdom is provided in Table S1. The most frequent primary indications were chronic non‐cancer pain (n = 2276; 60.79%) and neuropathic pain (n = 600; 16.03%), with the full distribution of primary indications presented in Figure 1 and the primary, secondary, and tertiary indications in Table S2. Additional comorbidities and the distribution of the Charlson Comorbidity Index are detailed in Table S3.

**Table 1:** Demographics, Tobacco, Alcohol, and Cannabis Consumption

Demographics	Mean ± SD / Median [IQR] / n (%)
**Age (years)**	46.36 ± 13.92
**Sex**	
Female	1572 (41.99%)
Male	2172 (58.01%)
**Occupation**	
Armed forces occupations	6 (0.16%)
Clerical support workers	89 (2.38%)
Craft and related trades workers	137 (3.66%)
Elementary occupations	107 (2.86%)
Managers	200 (5.34%)
Other occupations	626 (16.72%)
Plant and machine operators, and assemblers	33 (0.88%)
Professional	445 (11.89%)
Retired	54 (1.44%)
Service and sales workers	125 (3.34%)
Skilled agricultural, forestry, and fishery workers	18 (0.48%)
Technicians and associate professionals	187 (4.99%)
Unemployed	1560 (41.67%)
Not recorded	157 (4.19%)
**Weight (kg)**	82.22 ± 21.95
**Height (cm)**	172.21 ± 10.94
**BMI (kg/m^2^)**	27.73 ± 7.26
**Charlson comorbidity index**	1.00 [0.00–6.00]
**Smoking status**	
Current smoker	1090 (29.11%)
Ex‐smoker	1590 (42.47%)
Never smoked	1064 (28.42%)
**Lifetime smoking exposure (pack years)** ^a^	10.00 [4.00–20.00]
**Weekly alcohol consumption (units)**	0.00 [0.00–4.00]
**Cannabis consumer status**	
Current cannabis consumer	2152 (57.48%)
Former cannabis consumer	590 (15.76%)
Never consumed cannabis	1002 (26.76%)
**Cannabis use frequency** ^b^	
Every day	1833 (85.18%)
Every other day	164 (7.62%)
1–2 times per week	116 (5.39%)
>1 times per month	20 (0.93%)
<1 times per month	19 (0.88%)
**Cannabis consumption method** ^b^	
Ingestion	862 (40.06%)
Smoking	1253 (58.22%)
Topical	150 (6.97%)
Vaporization (flower)	1421 (66.03%)
Vaporization (liquid)	3 (0.14%)
**Daily cannabis consumption (g)** ^b^	1.00 [1.00–2.00]
**Lifetime cannabis exposure (gram years)** ^c^	8.00 [2.00–20.00]

BMI, body mass index; IQR, interquartile range; SD, standard deviation.

Note: Missing data: occupation (n = 157; 4.19%); weight (n = 172; 4.59%); height (n = 171; 4.57%); BMI (n = 184; 4.91%).

^a^ Calculated from current and ex‐smokers.

^b^ Calculated from current cannabis consumers.

^c^ Calculated from current and former cannabis consumers.

**Figure 1:**
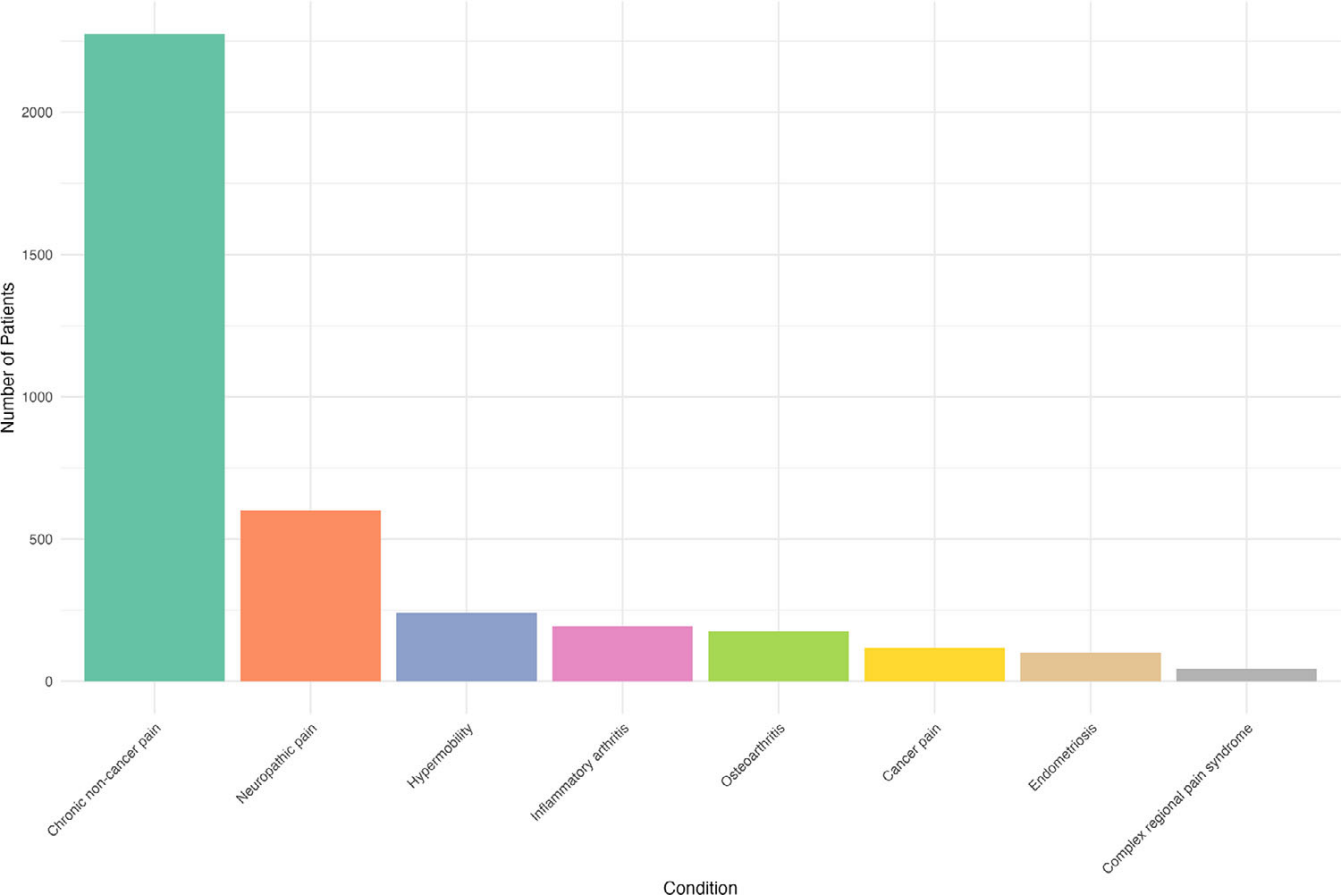
Distribution of primary indications for treatment with cannabis‐based medicinal products (CBMPs).

### Follow‐Up and Missing Data

Observed BPI‐SF pain‐severity scores were available for 3658 (97.70%) participants at baseline, 3112 (83.12%) at 1 month, 2637 (70.43%) at 3 months, 2206 (58.92%) at 6 months, 1863 (49.76%) at 12 months, 1600 (42.74%) at 18 months, and 1309 (34.96%) at 24 months (Table S4). Completion was comparable across all PROMs, with between 1269 (33.89%) and 1348 (36.00%) participants completing each measure at 24 months.

Participants with an observed BPI‐SF pain‐severity score at 24 months had a marginally lower baseline pain severity than those without (5.86 ± 1.70 vs 6.04 ± 1.72; P =  .002; d = −0.10). There was no difference in age, baseline pain interference, GAD‐7, or SQS (all *P* ≥  .050; Table S5). Completers were more likely to be male and current or former cannabis consumers, and less likely to be unemployed or to have a primary indication of cancer pain (all *P* <  .050). In a complete‐case analysis of participants with observed data at both baseline and 24 months (n = 1309), BPI‐SF pain severity improved from 5.86 ± 1.70 to 4.55 ± 1.96 (mean difference 1.31 ± 1.87; *P* < .001; d = 0.70), and 742 (56.68%) achieved the MCID. These estimates were larger than those derived from the imputed dataset.

### Cannabis‐Based Medicinal Products

At the commencement of treatment, participants were most frequently prescribed both dried flower/flos and oil (n = 1732; 46.26%) or oil alone (n = 1614; 43.11%), with a smaller proportion prescribed dried flower/flos alone (n = 383; 10.23%; Table S6). At 24 months, the corresponding proportions were 1748 (46.69%), 879 (23.48%), and 801 (21.39%), alongside an increasing use of combinations incorporating pastilles or other formulations. The median daily CBD dose increased from 15.00 [1.50–20.00] mg/day at baseline to 20.53 [11.00–50.21] mg/day at 24 months, and the median daily THC dose from 19.00 [1.00–22.00] mg/day to 126.50 [26.19–247.50] mg/day (Table S6).

### Oral Morphine Equivalent and Pregabalin Equivalent

With repeated‐measures analysis of variance a difference in daily prescribed OME and pregabalin‐equivalent dose was observed across the studied time periods (both *P* <  .001; Tables S7–S10). The mean prescribed OME at baseline was 33.09 ± 68.32 mg/day. No difference was detected at 1 month (*P* = 1.000; d = 0.05), although a reduction was observed at 3, 6, 12, 18, and 24 months (*P* <  .050; Table S8). Nearly one in four participants prescribed opioids at baseline reported an MCID reduction in OME by 24 months (n = 302; 22.66%; Table S9). The mean pregabalin‐equivalent dose at baseline was 294.55 ± 255.42 mg/day, with no difference at 1 or 3 months (*P* ≥  .050), but a reduction from 6 months onward (*P* <  .050; Table S10).

### Adverse Events

A total of 538 participants (14.37%) reported one or more adverse events. There were 4898 events in total, graded as mild (n = 2027; 41.38%), moderate (n = 2143; 43.75%), severe (n = 720; 14.70%), or life‐threatening/disabling (n = 8; 0.16%). The severity distribution of the 10 most frequent events is illustrated in Figure 2. The most frequently reported events were fatigue (n = 400; 10.68%), dry mouth (n = 303; 8.09%), lethargy (n = 300; 8.01%), and somnolence (n = 300; 8.01%) (Table S11).

**Figure 2:**
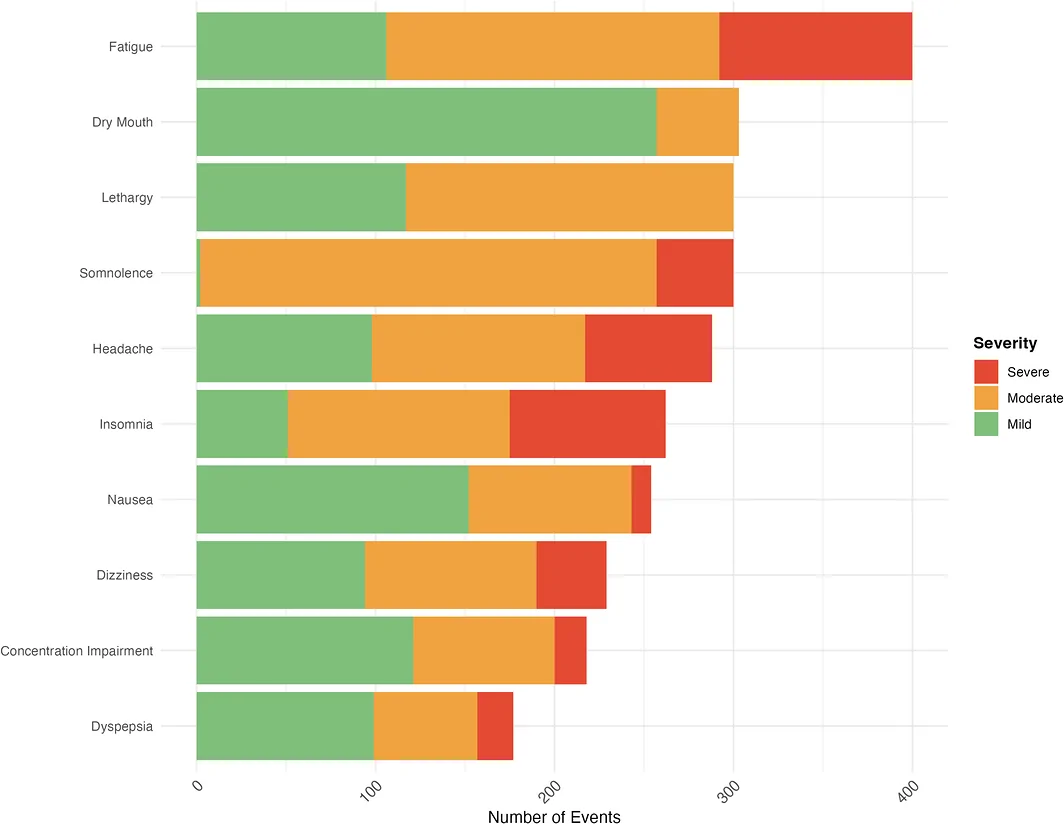
Severity distribution of the 10 most frequent adverse events. Adverse events graded per the Common Terminology Criteria for Adverse Events version 4.0.

Tables S12 and S13 detail the univariable and multivariable logistic regression for reporting one or more adverse events. On multivariable analysis, increasing age was associated with greater odds of an adverse event, relative to those aged under 30 years (51–60 years OR 1.78, 95% CI: 1.20–2.66; *P* =  .004; over 60 years OR 2.18, 95% CI: 1.44–3.35; *P* <  .001), as were female sex (OR 1.53, 95% CI: 1.23–1.91; *P* <  .001), a primary diagnosis of hypermobility (OR 1.84, 95% CI: 1.28–2.62; *P* <  .001), and a daily CBD dose in the highest quartile at 24 months (OR 1.81, 95% CI: 1.35–2.45; *P* <  .001). Current cannabis use at baseline (OR 0.47, 95% CI: 0.36–0.62; *P* <  .001) and a primary diagnosis of cancer pain (OR 0.19, 95% CI: 0.06–0.43; *P* <  .001) were each associated with lower odds of reporting an adverse event.

### Patient‐Reported Outcome Measures

A difference across the seven timepoints was observed for every PROM (all *P* <  .001; Table 2), with full pairwise comparisons presented in Tables S14–S29. For the primary measure of pain severity, the BPI‐SF pain‐severity score improved from baseline (5.97 ± 1.71) to 1 (5.06 ± 1.82; *P* <  .001; d = 0.53), 3 (4.87 ± 1.80; *P* <  .001; d = 0.63), 6 (4.76 ± 1.81; *P* <  .001; d = 0.65), 12 (4.77 ± 1.75; *P* <  .001; d = 0.65), 18 (4.73 ± 1.77; *P* <  .001; d = 0.63), and 24 months (4.86 ± 1.67; *P* <  .001; d = 0.57; Table S23). Parallel improvements from baseline to 24 months were observed for BPI‐SF pain interference (mean difference 1.49 ± 2.52; *P* <  .001; d = 0.59), SF‐MPQ‐2 total score (mean difference 1.19 ± 2.12; *P* <  .001; d = 0.56), and the pain VAS (mean difference 1.22 ± 2.67; *P* <  .001; d = 0.46). Furthermore, the EQ‐5D‐5L index value (mean difference −0.23 ± 0.31; *P* <  .001; d = −0.76), the GAD‐7 (mean difference 3.68 ± 6.25; *P* <  .001; d = 0.59), and the SQS (mean difference −1.92 ± 2.91; *P* <  .001; d = −0.66) improved from baseline to 24 months (Table 2; Tables S14–S22 and S24–S29).

**Table 2:** Outcomes of Repeated‐Measures Analysis of Variance for Patient‐Reported Outcome Measures at Baseline, 1, 3, 6, 12, 18, and 24 Months

	Mean ± SD							
Patient‐Reported Outcome Measures	Baseline	1 Month	3 Months	6 Months	12 Months	18 Months	24 Months	*P*‐value
EQ‐5D‐5L mobility	2.88 ± 1.11	2.60 ± 1.08	2.57 ± 1.05	2.52 ± 1.03	2.50 ± 1.02	2.48 ± 1.01	2.54 ± 1.01	<.001
EQ‐5D‐5L self‐care	2.17 ± 1.09	2.01 ± 1.01	1.99 ± 0.99	1.96 ± 0.96	1.97 ± 0.94	1.95 ± 0.92	1.98 ± 0.93	<.001
EQ‐5D‐5L usual activities	3.08 ± 1.11	2.58 ± 1.04	2.56 ± 1.01	2.49 ± 0.97	2.50 ± 0.95	2.47 ± 0.96	2.46 ± 0.96	<.001
EQ‐5D‐5L pain and discomfort	3.82 ± 0.89	3.07 ± 0.92	2.95 ± 0.88	2.91 ± 0.86	2.94 ± 0.83	2.91 ± 0.84	2.88 ± 0.82	<.001
EQ‐5D‐5L anxiety and depression	2.52 ± 1.21	2.11 ± 1.04	2.09 ± 0.99	2.06 ± 0.98	2.06 ± 0.97	2.03 ± 0.96	1.99 ± 0.94	<.001
EQ‐5D‐5L index value	0.30 ± 0.31	0.49 ± 0.27	0.51 ± 0.26	0.52 ± 0.25	0.53 ± 0.23	0.53 ± 0.23	0.53 ± 0.23	<.001
GAD‐7	8.51 ± 6.42	5.55 ± 5.10	5.60 ± 5.04	5.42 ± 4.84	5.29 ± 4.76	5.29 ± 4.74	4.83 ± 4.42	<.001
SQS	3.80 ± 2.36	5.53 ± 2.51	5.57 ± 2.47	5.60 ± 2.46	5.60 ± 2.43	5.68 ± 2.50	5.73 ± 2.47	<.001
PGIC		5.00 ± 1.54	5.32 ± 1.36	5.51 ± 1.27	5.61 ± 1.30	5.61 ± 1.30	5.84 ± 1.24	<.001
BPI‐SF pain severity	5.97 ± 1.71	5.06 ± 1.82	4.87 ± 1.80	4.76 ± 1.81	4.77 ± 1.75	4.73 ± 1.77	4.86 ± 1.67	<.001
BPI‐SF pain interference	6.87 ± 2.14	5.55 ± 2.41	5.31 ± 2.38	5.12 ± 2.37	5.10 ± 2.31	5.32 ± 2.32	5.38 ± 2.20	<.001
SF‐MPQ‐2 total	4.80 ± 1.99	3.86 ± 1.98	3.54 ± 1.95	3.55 ± 1.87	3.73 ± 1.99	3.69 ± 1.79	3.61 ± 1.56	<.001
SF‐MPQ‐2 continuous pain	5.74 ± 2.17	4.74 ± 2.21	4.36 ± 2.18	4.31 ± 2.37	4.50 ± 2.13	3.60 ± 2.47	5.08 ± 2.58	<.001
SF‐MPQ‐2 intermittent pain	4.91 ± 2.46	3.99 ± 2.55	3.65 ± 2.36	3.65 ± 2.51	3.76 ± 2.38	3.30 ± 2.41	3.10 ± 2.28	<.001
SF‐MPQ‐2 neuropathic pain	3.64 ± 2.35	2.92 ± 2.12	2.83 ± 2.08	2.78 ± 2.21	3.19 ± 2.48	2.42 ± 2.52	1.87 ± 2.31	<.001
Pain VAS	7.12 ± 1.98	5.99 ± 2.35	5.75 ± 2.37	5.62 ± 2.42	5.73 ± 2.37	5.64 ± 2.37	5.90 ± 2.36	<.001

BPI‐SF, Brief Pain Inventory Short Form; EQ‐5D‐5L, EuroQol 5‐Dimension 5‐Level; GAD‐7, Generalised Anxiety Disorder‐7; PGIC, Patient Global Impression of Change; SD, standard deviation; SF‐MPQ‐2, Short‐Form McGill Pain Questionnaire‐2; SQS, Single‐Item Sleep Quality Scale; VAS, visual analogue scale.

*Note*: Values are derived from the multiply imputed dataset (n = 3744). The number of observed responses for each measure at each timepoint is reported in Table S4.

Approximately three quarters of participants achieved a positive improvement in the EQ‐5D‐5L index value at each timepoint. This reached 76.71% (n = 2872) at 24 months, at which point 42.39% (n = 1587) and 39.10% (n = 1464) had also achieved the MCID in the GAD‐7 and SQS, respectively (Table S30). Both BPI‐SF subscales showed progressively smaller proportions of individuals meeting increasingly stringent thresholds at 24 months. For pain severity, 54.62% (n = 2045) achieved the MCID, 34.32% (n = 1285) an MII, and 14.88% (n = 557) an SII. For pain interference, the corresponding proportions were 62.77% (n = 2350), 42.07% (n = 1575), and 21.21% (n = 794). More than half of participants achieved the MCID for each of the remaining pain‐specific PROMs at 24 months. The full set of MCID, MII, and SII proportions is presented in Table S31.

The univariable and multivariable logistic regression models for a clinically significant improvement in each PROM at 24 months are detailed in Tables S32–S45. Across outcomes, poorer baseline sleep quality and higher baseline anxiety were the most consistent independent predictors of improvement (*P* <  .050). Relative to good baseline sleep (SQS 7–10), poor baseline sleep (SQS 0–3) was associated with higher odds of achieving the MCID in BPI‐SF pain severity (OR 2.24, 95% CI: 1.82–2.76; *P* <  .001) and pain VAS (OR 2.07, 95% CI: 1.69–2.55; *P* <  .001). Severe baseline anxiety (GAD‐7 ≥15) was associated with higher odds of improvement in BPI‐SF pain severity (OR 1.30, 95% CI: 1.09–1.56; *P* =  .004). Baseline opioid and gabapentinoid prescription were each associated with greater odds of improvement in the EQ‐5D‐5L index value (OR 1.25, 95% CI: 1.05–1.49; *P* =  .014 and OR 1.33, 95% CI: 1.06–1.68; *P* =  .015, respectively). A primary diagnosis of cancer pain was associated with lower odds of improvement in BPI‐SF pain interference (OR 0.59, 95% CI: 0.39–0.89; *P* =  .011) and pain VAS (OR 0.63, 95% CI: 0.42–0.95; *P* =  .027).

### k‐Means Clustering

The gap statistic identified a five‐cluster solution as optimal (Figure 3). The five clusters (A–E) were derived from 100 random initializations with k‐means optimization, the final solution being that with the highest silhouette score (Figure 4; Table S46). Cluster A was the largest (n = 956) and Cluster E the smallest (n = 488), with Clusters B (n = 946), C (n = 679), and D (n = 675) intermediate in size. The clusters displayed distinct pain‐severity trajectories. Cluster A began at moderate baseline severity (5.85 ± 1.15) and showed an initial improvement to a nadir of 5.12 at 3 months, followed by a return toward baseline, ending essentially unchanged at 24 months (5.79 ± 1.00; net change −0.06 points). Cluster B achieved an early reduction to 3.67 at 3 months that partially attenuated by 24 months (5.10 ± 1.33 at baseline to 4.44 ± 1.23; net change −0.66 points). Clusters C and D had the highest baseline severity (7.72 ± 1.15 and 6.86 ± 1.19, respectively), with Cluster C improving gradually and modestly (to 6.30 ± 1.26; net change −1.41 points) and Cluster D demonstrating the largest progressive and sustained reduction of any cluster (to 4.03 ± 1.17; net change −2.84 points). Cluster E had the mildest baseline severity (4.26 ± 1.67) and achieved a rapid improvement within the first month that was thereafter maintained, ending at 2.57 ± 1.36 at 24 months (net change −1.69 points).

**Figure 3:**
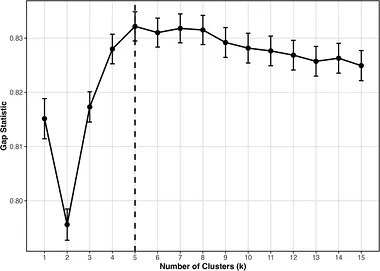
Gap statistic according to number of clusters (k). The optimal number of clusters of Brief Pain Inventory Short Form (BPI‐SF) pain‐severity trajectories between baseline, 1, 3, 6, 12, 18, and 24 months was determined using the gap statistic method, computed with 1000 bootstrap samples (K.max = 15). The mean gap statistic and standard deviation are plotted. The vertical dotted line indicates the value of k with the highest mean gap statistic.

**Figure 4:**
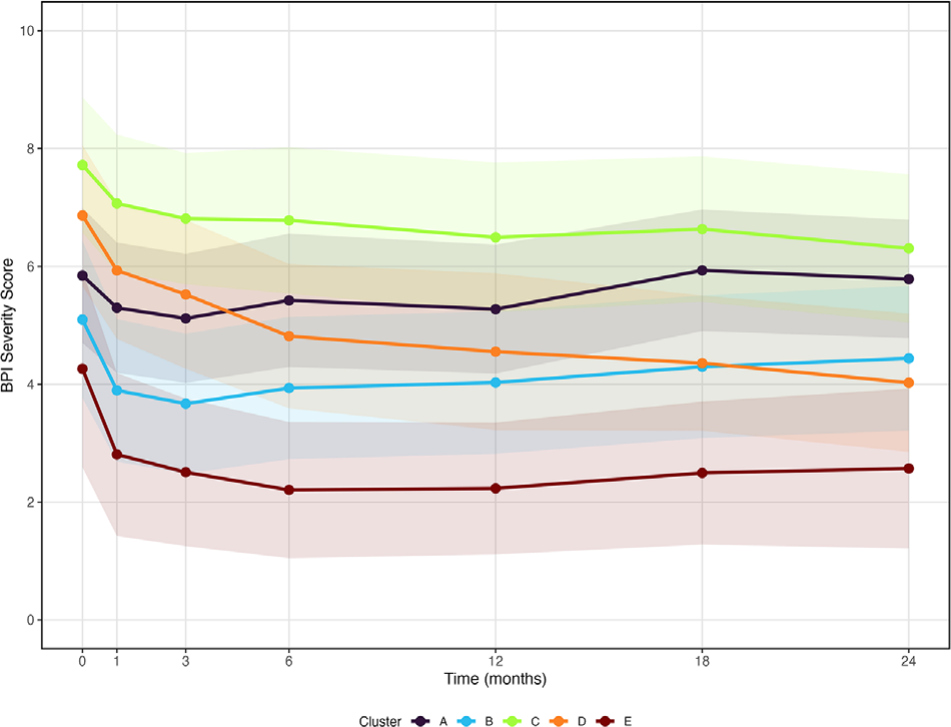
Mean trajectories of Brief Pain Inventory Short Form (BPI‐SF) pain severity across five clusters. Assignments to five clusters were determined based on 100 random initializations (nstart = 100) following the standard k‐means optimization procedure. Shaded regions represent one standard deviation.

### Univariable Logistic Regression of Cluster Membership

Univariable analysis examined 515 associations across 34 predictors, identifying 72 statistically significant associations (Figure 5; Table S47; *P* <  .050). Baseline sleep quality and generalized anxiety emerged as the strongest discriminators, each yielding six significant associations, followed by BMI category (five) and occupation status (four), all significant at *P* <  .050. Poor baseline sleep quality (SQS 0–3), relative to good sleep (SQS 7–10), was positively associated with membership of the higher‐severity Clusters C (OR 2.75, 95% CI: 2.06–3.73; *P* <  .001) and D (OR 2.49, 95% CI: 1.87–3.38; *P* <  .001) and negatively associated with the mild, rapidly improving Cluster E (OR 0.34, 95% CI: 0.27–0.44; *P* <  .001).

**Figure 5:**
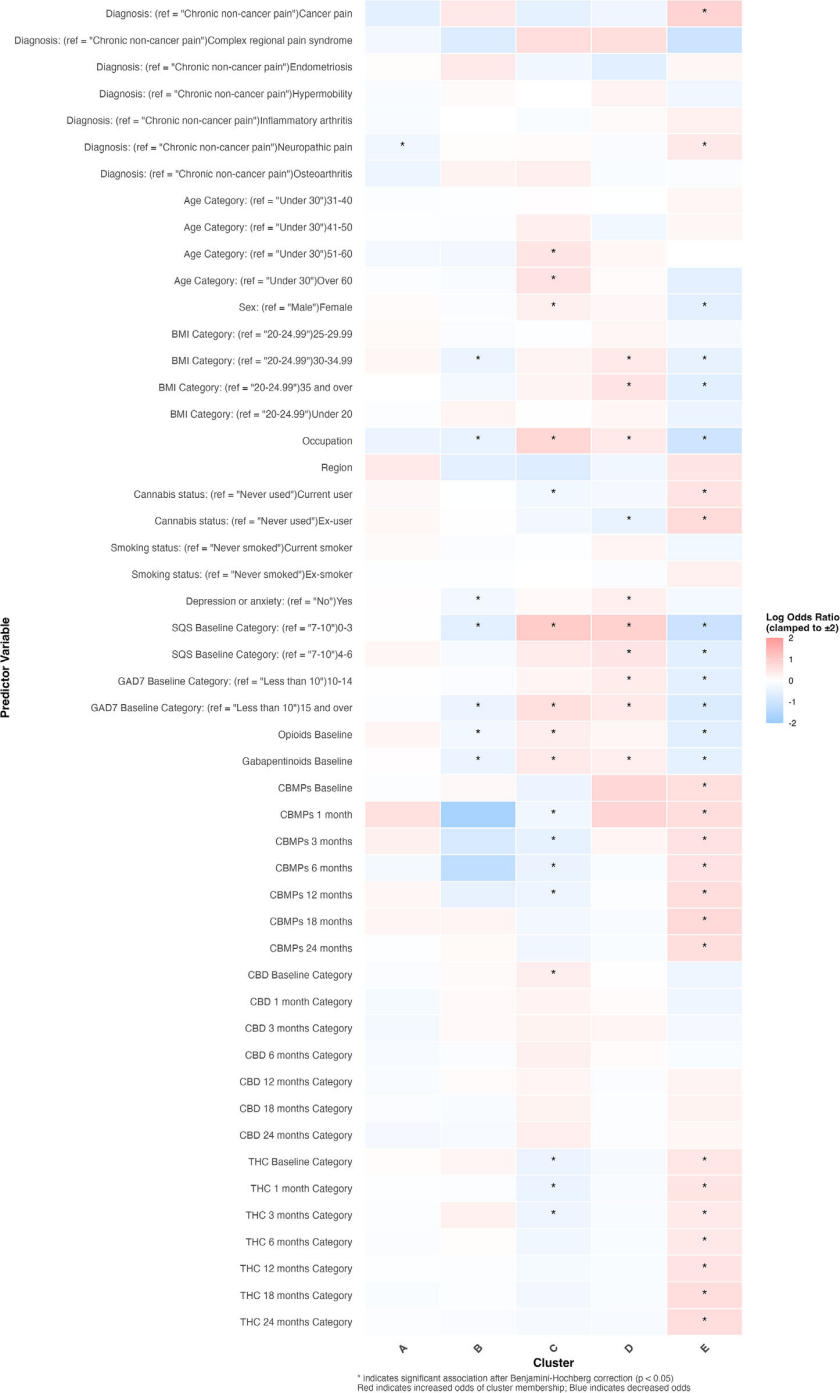
Heatmap of associations between independent variables and cluster membership on univariable analysis. Red indicates a positive association, blue a negative association according to log(odds ratio). Strength clamped at  ± 2. * *P* <  .050 with Benjamini–Hochberg correction. BMI, body mass index; CBD, cannabidiol; CBMPs, cannabis‐based medicinal products; GAD‐7, Generalised Anxiety Disorder‐7; PTSD, post‐traumatic stress disorder; SQS, Single‐Item Sleep Quality Scale; THC, (−)‐trans‐Δ^9^‐tetrahydrocannabinol.

### Stepwise Multivariable Logistic Regression of Cluster Membership

Stepwise selection retained 21 of the candidate predictors, yielding 71 significant associations across the five clusters (Table S48; *P* <  .050). Baseline sleep quality and occupation status remained among the strongest discriminators. Poor baseline sleep (SQS 0–3) was positively associated with Clusters C (OR 2.31, 95% CI: 1.93–2.77; *P* <  .001) and D (OR 2.18, 95% CI: 1.82–2.61; *P* <  .001) and negatively associated with Clusters B (OR 0.59, 95% CI: 0.50–0.69; *P* <  .001) and E (OR 0.45, 95% CI: 0.38–0.53; *P* <  .001). Unemployment, relative to employment, followed a similar pattern, being positively associated with Clusters C (OR 1.87, 95% CI: 1.67–2.10) and D (OR 1.28, 95% CI: 1.14–1.43) and negatively with Clusters B (OR 0.66, 95% CI: 0.59–0.75) and E (OR 0.48, 95% CI: 0.42–0.54; all *P* <  .001). Severe baseline anxiety (GAD‐7 ≥15) and baseline opioid prescription showed a comparable pattern, each being positively associated with the higher‐severity Cluster C (anxiety OR 1.53, 95% CI: 1.34–1.76; opioid prescription OR 1.28, 95% CI: 1.14–1.43; both *P* <  .001) and negatively with Cluster E (anxiety OR 0.72, 95% CI: 0.62–0.84; opioid prescription OR 0.68, 95% CI: 0.60–0.76; both *P* <  .001). A primary diagnosis of cancer or neuropathic pain was positively associated with membership of Cluster E (cancer pain OR 3.09, 95% CI: 2.31–4.17; neuropathic pain OR 1.45, 95% CI: 1.25–1.68; both *P* <  .001).

### LASSO Multivariable Logistic Regression of Cluster Membership

LASSO regularization yielded 34 significant associations, with variable selection broadly consistent with the stepwise model (Table S49; *P* <  .050). Poor baseline sleep (SQS 0–3) again showed positive associations with Clusters C (OR 1.85, 95% CI: 1.66–2.07; *P* <  .001) and D (OR 1.62, 95% CI: 1.46–1.81; *P* <  .001) and negative associations with Clusters B (OR 0.66, 95% CI: 0.60–0.74; *P* <  .001) and E (OR 0.66, 95% CI: 0.59–0.74; *P* <  .001). A primary diagnosis of cancer or neuropathic pain retained a positive association with Cluster E (cancer pain OR 3.12, 95% CI: 2.35–4.17; neuropathic pain OR 1.40, 95% CI: 1.21–1.61; both *P* <  .001).

A narrative synthesis of the baseline characteristics associated with cluster membership on both the stepwise and LASSO multivariable models is presented in Table S50. Discrimination was comparable between the stepwise and LASSO models across all clusters, with the AUC ranging from 0.54 to 0.72 and from 0.59 to 0.71, respectively, and highest for Cluster E in both approaches (Figure S1).

## Discussion

This longitudinal analysis applied k‐means clustering to BPI‐SF pain‐severity trajectories in 3744 patients treated with CBMPs for chronic pain in the UK Medical Cannabis Registry over 24 months, identifying five distinct trajectory clusters and demonstrating that the pattern and magnitude of pain response are heterogeneous. Improvements were observed across every pain, health‐related quality of life, anxiety, and sleep outcome, accompanied by a reduction in prescribed opioid burden and an adverse‐event profile that was predominantly mild to moderate. In contrast to an earlier clustering analysis of health‐related quality of life in the same registry,^25^ every cluster reported improved symptoms at 24 months compared to baseline. Therefore, whilst there was still heterogeneity in this cohort it was instead reflected in the degree and durability of improvement. Membership of the five clusters was distinguished predominantly by baseline patient characteristics rather than by the specific CBMP formulation or dose prescribed.

At the level of the whole cohort, treatment with CBMPs was accompanied by improvements across every pain‐specific measure. Small‐to‐moderate effect sizes were reported from 1 month and maintained to 24 months. More than half of participants reported the MCID for each measure. Parallel improvements were observed in health‐related quality of life, generalized anxiety, and sleep quality. That these gains were sustained across 2 years is of note in a chronic and frequently treatment‐refractory population, in which spontaneous remission is uncommon and the probability of recovery over a comparable interval is low.^5^, ^59^ Prescribed opioid burden also fell, with 22.66% of participants receiving opioids at baseline achieving a clinically important reduction in OME by 24 months. This is consistent with the opioid‐sparing signal reported among observational studies in the systematic review by Noori and colleagues.^60^ However, this is dependent upon very low‐certainty evidence and these findings have yet to be routinely corroborated by randomized trials. Adverse events were reported by 14.37% of participants and were predominantly mild or moderate in severity.

Examination of the individual trajectories showed that the heterogeneity of response reflected differences in both the completeness and the durability of improvement. Clusters C, D, and E sustained a mean reduction beyond the one‐point MCID to 24 months. The remaining two clusters, together comprising just over half of the cohort, exhibited an initial improvement that was not maintained. Cluster B achieved a meaningful early reduction from baseline by 3 months that subsequently attenuated, leaving only a modest net change below the MCID at 24 months. Cluster A, the largest cluster, showed a smaller initial reduction that reversed, with mean severity returning to marginally below baseline at 24 months. This fluctuating, non‐sustained pattern is consistent with the recognized natural history of chronic pain, in which longitudinal latent class analysis of the HUNT general‐population cohort similarly identified five pain trajectories over 4 years, the largest of which was characterized by pain that fluctuated around the chronic‐pain threshold rather than remitting.^59^ The probability of spontaneous recovery from established moderate‐to‐severe chronic pain is approximately 8% over 1 year.^5^, ^59^ This is maintained in only around half of those who do recover. The sustained reductions observed in Clusters C, D, and E exceed what a natural course would predict, although the uncontrolled design precludes attributing this difference to treatment. The identification of a substantial subgroup in whom an early analgesic response is not sustained is nonetheless clinically relevant, as it indicates that initial improvement following CBMP initiation cannot be assumed to persist and supports structured reassessment of treatment response over time.

The magnitude and durability of the pain improvements observed exceed those reported in meta‐analyses of randomized controlled trials, in which the analgesic effect of cannabis‐based medicines has generally been small or inconsistent.^19^, ^20^, ^21^, ^61^, ^62^ Several features of the present study may account for this discrepancy. First, the uncontrolled, open‐label design means the responses are at risk of expectancy bias. A meta‐analysis of blinded cannabinoid pain trials found that the placebo response was amplified by disproportionately positive media attention.^63^ Second, registry participants represent a self‐selected population, many of whom were already accessing illicit cannabis prior to treatment. This may have conflicting effects on the outcomes reported. It represents a significant selection bias of identified responders to the effects of cannabis. However, these individuals may have already developed pharmacological tolerance to the effects of the major cannabinoids.^64^ Third, real‐world prescribing permits individualized titration across a range of formulations and doses that fixed‐dose trials do not, whilst regression to the mean would be expected to contribute to apparent improvement in a cohort enrolled at a point of high symptom burden. The study, therefore, cannot isolate the effects of CBMPs. Nonetheless, the sustained improvement documented under real‐world conditions remains of relevance to ongoing clinical practice. The trajectories identified here also carry implications for the design of future randomized trials. Pre‐specified responder analyses may help to distinguish a clinically meaningful effect within a subgroup from a small average effect across a heterogeneous population.^24^ Stratified randomization by the baseline characteristics associated with clinically important improvement, such as poor sleep quality and severe anxiety, would additionally allow these associations to be tested under controlled conditions. Serial assessment beyond the early treatment period would be required to determine whether an initial response is sustained.

The primary indication for treatment was among the characteristics most consistently associated with cluster membership. A primary diagnosis of cancer pain or neuropathic pain was positively associated, in both the stepwise and LASSO models, with membership of Cluster E, which had the mildest baseline severity and a rapid and sustained response. For neuropathic pain, this aligns with a broader body of evidence identifying it as one of the more promising indications for CBMPs. The mechanistic rationale is well established, as cannabinoids act on receptors at successive levels of the pain neuraxis, producing antinociception in preclinical models of neuropathic pain.^7^, ^8^, ^12^ Among randomized trials, the clearest signals of benefit have been reported for neuropathic indications.^6^, ^20^, ^65^ Moreover, the recent Phase 3 trial of the full‐spectrum extract VER‐01 in chronic low back pain met its secondary endpoint in the pre‐specified subgroup with a neuropathic pain component, in whom the Neuropathic Pain Symptom Inventory fell significantly relative to placebo.^23^ The association of cancer pain with a mild, rapidly improving trajectory should be interpreted more cautiously, as the evidence for CBMPs in cancer pain is weaker and less consistent.^19^, ^66^, ^67^ This association may therefore partly reflect case‐mix, including distinct pain mechanisms, or the effects of concurrent oncological treatment, rather than a specific effect of CBMPs. Participants with cancer pain were also the least likely of any indication to complete follow‐up, such that their trajectories relied more heavily on imputed values. As diagnoses were aggregated into broad categories, residual confounding by the specific underlying condition cannot be excluded.

Poor baseline sleep quality, severe baseline anxiety, and baseline prescription of opioids or gabapentinoids were each positively associated with membership of the higher‐baseline‐severity clusters, C and D. These features therefore appear to mark a more severe and complex pain presentation rather than a specific propensity either to respond or not to respond, since they characterized both the slowly improving Cluster C and the strongly improving Cluster D. Consistent with this, the regression analyses of clinically important improvement showed higher baseline anxiety and poorer sleep to be positively associated with achieving an MCID. The association was most evident for pain interference, for which both moderate and severe baseline anxiety were independently associated with improvement, alongside poor sleep. It was present but attenuated for pain severity, for which only severe anxiety reached significance. The association with poor sleep was of similar magnitude across both outcomes. A comparable pattern was reported in the earlier analysis of health‐related quality of life across all conditions in the registry, in which severe anxiety and poor sleep likewise predicted a more favorable response.^25^ Two mechanisms may contribute to this finding. First, patients with the most disturbed baseline sleep and the most severe anxiety have the greatest scope for improvement in these domains. Second, improvement in sleep and anxiety may reduce pain, particularly its interference with daily function.^68^, ^69^, ^70^, ^71^ Improvement in these domains can lessen pain‐related catastrophizing and fear avoidance and reduce attentional focus on pain, enhance the capacity to engage in physical activity and rehabilitation, and interrupt the bidirectional relationship between disturbed sleep and pain.^72^, ^73^, ^74^ That the association was stronger for pain interference than for pain severity is consistent with functional and affective gains rather than direct changes in nociception.

Demographic and substance use characteristics also distinguished the clusters. Female sex and older age were each less likely to be associated with membership of the mild, rapidly responding Cluster E, and female sex was additionally associated with a greater likelihood of reporting an adverse event. Sex differences in cannabinoid pharmacology are described in preclinical and emerging clinical evidence which indicates that females may be more sensitive than males to the effects of THC.^75^, ^76^, ^77^ This is secondary to differences in cannabinoid receptor expression, metabolism, and the influence of gonadal hormones on the endocannabinoid system.^75^, ^76^, ^77^ These associations may also be partly confounded by sex‐related differences in the prevalence of specific conditions, such as hypermobility‐related pain, that could not be fully controlled given the aggregation of diagnoses.^78^ Unemployment was associated with the higher‐severity clusters and employment with Cluster E, consistent with the strong and bidirectional relationship between chronic pain and reduced work participation.^79^ Finally, former cannabis use was inversely associated with membership of the progressively improving Cluster D, echoing the more favorable response observed among cannabis‐naïve patients in the registry's earlier analysis.^25^ This may reflect pharmacological tolerance or a ceiling effect in individuals who had already derived benefit from prior cannabis use.^64^

Across both multivariable approaches, treatment‐related variables were largely eliminated during stepwise selection or shrunk toward zero under LASSO regularization. The high collinearity between serial dosing measurements is likely to have contributed to their exclusion, and only a small number of early treatment variables were retained. Nonetheless, the overall pattern indicates that the baseline patient phenotype predicted the trajectory of pain response more strongly than the specific products subsequently prescribed. This does not imply that formulation or dose is unimportant as individualized titration may exert its effects within rather than between trajectory groups. Each prescription is also confounded by the underlying patient factors and the desired clinical effect. At the level of the whole cohort, the daily THC dose rose substantially over follow‐up, whilst the early improvement in Clusters A and B was not sustained. This pattern is compatible with the development of pharmacodynamic tolerance, which has been described for several effects of THC with repeated exposure.^18^ It may equally reflect upward titration in response to inadequate benefit, or the natural fluctuation of chronic pain. As most participants were current cannabis consumers at baseline, pre‐existing tolerance may also have limited the scope for further analgesic gain.^64^ Variability in bioavailability between formulations and routes of administration, and interactions with concomitant hepatically metabolized medicines, represent further sources of between‐patient variation that the registry was not designed to capture.^16^ Incorporating plasma cannabinoid concentrations alongside serial PROMs would allow exposure–response relationships to be examined within each trajectory.

This study has several limitations. As an observational analysis of routinely collected data, it cannot establish causation, and its findings are susceptible to selection and attrition bias, regression to the mean, and, given the open‐label context, expectancy effects. Adverse event and concurrent medication data were recorded on the assumption that the absence of a record indicated no event or no change, which may have led to underestimation of both. Missing PROM data increased over follow‐up, with approximately one in three participants contributing an observed BPI‐SF score at 24 months, and were handled by multiple imputation. The reported response proportions and trajectory estimates were consequently derived from the full enrolled cohort (n = 3744) rather than from the smaller number completing assessments at each timepoint. Multiple imputation performs less well as a method for handling missing data when data loss is not random.^52^ Loss to follow‐up may be related to treatment discontinuation, including discontinuation because of insufficient benefit or adverse events. Data are therefore unlikely to be missing completely at random, and participants with a less favorable course may be under‐represented at later timepoints. Reassuringly, completers differed only modestly from non‐completers in baseline pain severity, and the imputed estimates of improvement were more conservative than those from a complete‐case analysis. Nonetheless, trajectories in the later phase of follow‐up rest substantially on imputed values and should be interpreted with corresponding caution. The cohort was drawn from a single clinical service, which may limit generalizability. The aggregation of diagnoses into broad categories may cause residual confounding by the underlying condition. The clustering was performed on pain severity alone and therefore does not capture other dimensions of the pain experience, such as interference or health‐related quality of life. As the clustering was applied to the absolute BPI‐SF pain‐severity scores, including the baseline value, the resulting groups are separated by both their starting severity and their pattern of change over time, such that cluster membership partly encodes baseline pain level rather than the response to treatment alone.^80^ Reassuringly, clusters with comparable baseline severity nonetheless followed markedly divergent courses indicating that the solution captured genuine heterogeneity in the pattern of response and not simply a stratification by baseline pain.^81^ Finally, the absence of a comparator group receiving standard care precludes any conclusion regarding the comparative effectiveness of CBMPs.

## Conclusion

In this longitudinal analysis of 3744 patients treated with CBMPs for chronic pain, k‐means clustering identified five distinct trajectories of pain severity over 24 months. Every trajectory improved relative to baseline value. Yet, the clusters differed markedly in the degree and durability of improvement. For example, the largest showed no clinically meaningful net change. Membership of these trajectories was distinguished predominantly by baseline patient characteristics rather than by the specific products prescribed. Alongside sustained improvements across pain, quality‐of‐life, anxiety, and sleep outcomes, and a reduction in opioid burden, participants reported a predominantly mild‐to‐moderate adverse‐event profile. These findings characterize the heterogeneity of real‐world response to CBMPs and identify baseline phenotypes that may help to anticipate which patients sustain benefit and which warrant earlier reassessment. Further assessment through randomized controlled trials or novel methodologies utilizing real‐world evidence will be further needed to identify the true effect of CBMPs.

## Conflicts of Interest

SE is the Research Director for Curaleaf Clinic. AD, AM, DV are Research Associates at Curaleaf Clinic. RC is the Operations Director at Curaleaf Clinic. SB, SAK, MWW, MWP, and JJR are clinicians at Curaleaf Clinic. MHS is the Chief Medical Officer for Curaleaf International.

## Supporting information



Supporting Information

Supporting Information

Supporting Information

Supporting Information

Supporting Information

## Data Availability

The data that support the findings of this study are available from the UK Medical Cannabis Registry. Restrictions apply to the availability of these data, which were used under licence for the current study and are not publicly available. Data are available from the authors upon reasonable request and with the permission of the UK Medical Cannabis Registry. Data specifications and applications are available from the corresponding author.
