# Supplementary material for: Pain Severity Trajectories and Patient Profiles in Chronic Pain Patients Treated with Cannabis‐Based Medicinal Products: A k‐Means Clustering Analysis of the UK Medical Cannabis Registry
Source: J Clin Pharmacol. 2026 Oct 16;66(10):e70313. doi: 10.1002/jcph.70313 (PMC13673507; doi:10.1002/jcph.70313)
Supplement: Supplementary file 1 [file JCPH-66-0-s001.docx]

**Supplementary Table 1. Geographic Breakdown of Study Population by Government Office Region**

| **Government Office Region** | **n (%)** |
| --- | --- |
| **Channel Islands** | 114 (3.04%) |
| **East Midlands** | 166 (4.43%) |
| **East of England** | 264 (7.05%) |
| **Isle of Man** | 28 (0.75%) |
| **London** | 423 (11.30%) |
| **North East** | 170 (4.54%) |
| **North West** | 340 (9.08%) |
| **Northern Ireland** | 116 (3.10%) |
| **Scotland** | 821 (21.93%) |
| **South East** | 468 (12.50%) |
| **South West** | 306 (8.17%) |
| **Wales** | 161 (4.30%) |
| **West Midlands** | 199 (5.32%) |
| **Yorkshire and the Humber** | 168 (4.49%) |

**Supplementary Table 2. Primary, Secondary and Tertiary Indications for Treatment with Cannabis-Based Medicinal Products (CBMPs)**

| **Condition** | **Primary Indication n (%)** | **Secondary Indication n (%)** | **Tertiary Indication n (%)** |
| --- | --- | --- | --- |
| **Agoraphobia** | - | 2 (0.05%) | - |
| **Anxiety** | - | 98 (2.62%) | 30 (0.80%) |
| **Attention-Deficit/Hyperactivity Disorder** | - | 6 (0.16%) | 4 (0.11%) |
| **Autism Spectrum Disorder** | - | 11 (0.29%) | 6 (0.16%) |
| **Chemotherapy-Induced Nausea and Vomiting** | - | 1 (0.03%) | - |
| **Chronic Cancer Pain** | 116 (3.10%) | 2 (0.05%) | - |
| **Chronic Non-Cancer Pain** | 2,276 (60.79%) | 68 (1.82%) | 8 (0.21%) |
| **Cluster Headache** | - | 2 (0.05%) | - |
| **Complex Regional Pain Syndrome** | 44 (1.18%) | 11 (0.29%) | 3 (0.08%) |
| **Crohn’s Disease** | - | 6 (0.16%) | 4 (0.11%) |
| **Depression** | - | 23 (0.61%) | 37 (0.99%) |
| **Eating Disorder** | - | - | 1 (0.03%) |
| **Endometriosis** | 101 (2.70%) | 4 (0.11%) | 1 (0.03%) |
| **Epilepsy (adult)** | - | 5 (0.13%) | 4 (0.11%) |
| **Fibromyalgia** | - | 141 (3.77%) | 18 (0.48%) |
| **Headache** | - | 11 (0.29%) | 4 (0.11%) |
| **Hypermobility** | 240 (6.41%) | 33 (0.88%) | 11 (0.29%) |
| **Inflammatory Arthritis** | 192 (5.13%) | 5 (0.13%) | 1 (0.03%) |
| **Insomnia** | - | 23 (0.61%) | 17 (0.45%) |
| **Migraine** | - | 26 (0.69%) | 8 (0.21%) |
| **Multiple Sclerosis** | - | 3 (0.08%) | - |
| **Neuropathic Pain** | 600 (16.03%) | 70 (1.87%) | 11 (0.29%) |
| **Obsessive Compulsive Disorder** | - | 2 (0.05%) | 1 (0.03%) |
| **Osteoarthritis** | 175 (4.67%) | 26 (0.69%) | 5 (0.13%) |
| **Palliative Care** | - | 1 (0.03%) | - |
| **Panic Disorder** | - | - | 1 (0.03%) |
| **Parkinson's** | - | 3 (0.08%) | - |
| **Post-Traumatic Stress Disorder** | - | 28 (0.75%) | 14 (0.37%) |
| **Rare and Challenging Skin Condition** | - | 1 (0.03%) | - |
| **Social Phobia** | - | 1 (0.03%) | - |
| **Trigeminal Neuralgia** | - | 4 (0.11%) | 2 (0.05%) |
| **Ulcerative Colitis** | - | 3 (0.08%) | 1 (0.03%) |

**Supplementary Table 3. Proportion of Collected Comorbidities in the Study Population**

| **Comorbidities** | **n (%)** |
| --- | --- |
| **Acquired Immunodeficiency Syndrome** | 0 (0%) |
| **Arthritis** | 1011 (27.00%) |
| **Cerebrovascular Accident** | 113 (3.02%) |
| **Chronic Obstructive Pulmonary Disease** | 120 (3.21%) |
| **Congestive Heart Failure** | 36 (0.96%) |
| **Connective Tissue Disease** | 454 (12.13%) |
| **Dementia** | 16 (0.43%) |
| **Depression / Anxiety** | 1137 (30.37%) |
| **Diabetes** |  |
| **Uncomplicated** | 184 (4.91%) |
| **End Organ Damage** | 56 (1.50%) |
| **Endocrine / Thyroid Dysfunction** | 222 (5.93%) |
| **Epilepsy** | 93 (2.48%) |
| **Hemiplegia** | 45 (1.20%) |
| **Hypertension** | 377 (10.07%) |
| **Leukaemia** | 23 (0.61%) |
| **Liver Disease** |  |
| **Mild** | 77 (2.06%) |
| **Moderate to Severe** | 19 (0.51%) |
| **Lymphoma** | 29 (0.77%) |
| **Moderate to Severe Chronic Kidney Disease** | 100 (2.67%) |
| **Myocardial Infarction** | 81 (2.16%) |
| **Peptic Ulcer Disease** | 99 (2.64%) |
| **Peripheral Vascular Disease** | 50 (1.34%) |
| **Solid Tumour** |  |
| **Localised** | 162 (4.33%) |
| **Metastatic** | 83 (2.22%) |
| **Venous Thromboembolism** | 123 (3.29%) |

**Supplementary Table 4. Number of Observed (Non-Imputed) Responses for Each Patient-Reported Outcome Measure at Baseline, 1, 3, 6, 12, 18, and 24 Months.**

Values are presented as n (%) of the full cohort (n=3,744) before multiple imputation. The number of observed responses for each SF-MPQ-2 subscale and EQ-5D-5L domain was identical to that of the parent measure at each timepoint, except for the EQ-5D-5L mobility, self-care, usual activities, and pain and discomfort domains at baseline (n=3,725; 99.49%). The PGIC was not administered at baseline. *BPI-SF – Brief Pain Inventory Short Form; EQ-5D-5L – EuroQol 5-Dimension 5-Level; GAD-7 – Generalised Anxiety Disorder-7; PGIC – Patient Global Impression of Change; SF-MPQ-2 – Short-Form McGill Pain Questionnaire-2; SQS – Single-Item Sleep Quality Scale; VAS – visual analogue scale.*

| **Patient-Reported Outcome Measure** | **Baseline** | **1 month** | **3 months** | **6 months** | **12 months** | **18 months** | **24 months** |
| --- | --- | --- | --- | --- | --- | --- | --- |
| **EQ-5D-5L index value** | 3,724 (99.47%) | 3,192 (85.26%) | 2,704 (72.22%) | 2,262 (60.42%) | 1,908 (50.96%) | 1,638 (43.75%) | 1,342 (35.84%) |
| **GAD-7** | 3,739 (99.87%) | 3,210 (85.74%) | 2,717 (72.57%) | 2,270 (60.63%) | 1,903 (50.83%) | 1,643 (43.88%) | 1,348 (36.00%) |
| **SQS** | 3,700 (98.82%) | 3,170 (84.67%) | 2,685 (71.71%) | 2,247 (60.02%) | 1,898 (50.69%) | 1,629 (43.51%) | 1,335 (35.66%) |
| **PGIC** | – | 3,176 (84.83%) | 2,675 (71.45%) | 2,233 (59.64%) | 1,878 (50.16%) | 1,609 (42.98%) | 1,314 (35.10%) |
| **BPI-SF pain severity** | 3,658 (97.70%) | 3,112 (83.12%) | 2,637 (70.43%) | 2,206 (58.92%) | 1,863 (49.76%) | 1,600 (42.74%) | 1,309 (34.96%) |
| **BPI-SF pain interference** | 3,658 (97.70%) | 3,112 (83.12%) | 2,637 (70.43%) | 2,206 (58.92%) | 1,863 (49.76%) | 1,600 (42.74%) | 1,309 (34.96%) |
| **SF-MPQ-2 total** | 3,512 (93.80%) | 2,990 (79.86%) | 2,544 (67.95%) | 2,141 (57.18%) | 1,810 (48.34%) | 1,550 (41.40%) | 1,269 (33.89%) |
| **Pain VAS** | 3,637 (97.14%) | 3,077 (82.18%) | 2,613 (69.79%) | 2,192 (58.55%) | 1,856 (49.57%) | 1,589 (42.44%) | 1,301 (34.75%) |

**Supplementary Table 5. Baseline Characteristics of Participants With and Without an Observed Brief Pain Inventory Short Form (BPI-SF) Pain Severity Score at 24 Months.**

Continuous variables were compared with Welch's t-test and categorical variables with the χ² test. Percentages are calculated within each column. Denominators for baseline patient-reported outcome measures vary slightly according to baseline completion. *BMI – body mass index; BPI-SF – Brief Pain Inventory Short Form; CBD – cannabidiol; EQ-5D-5L – EuroQol 5-Dimension 5-Level; GAD-7 – Generalised Anxiety Disorder-7; SD – standard deviation; SF-MPQ-2 – Short-Form McGill Pain Questionnaire-2; SQS – Single-Item Sleep Quality Scale; THC – (−)-trans-Δ^9^-tetrahydrocannabinol; VAS – visual analogue scale.*

| **Characteristic** | **Observed at 24 Months (n=1,309)** | **Not Observed at 24 Months (n=2,435)** | **p-value** |
| --- | --- | --- | --- |
| **Age (years), mean ± SD** | 46.61 ± 11.81 | 46.22 ± 14.93 | 0.381 |
| **Female sex, n (%)** | 518 (39.57%) | 1,054 (43.29%) | 0.031 |
| **BMI (kg/m²), mean ± SD** | 27.91 ± 7.10 | 27.63 ± 7.35 | 0.270 |
| **Unemployed, n (%)** | 492 (37.59%) | 1,068 (43.86%) | <0.001 |
| **Cannabis status, n (%)** |  |  | <0.001 |
| Current consumer | 855 (65.32%) | 1,297 (53.26%) |  |
| Former consumer | 229 (17.49%) | 361 (14.83%) |  |
| Cannabis naïve | 225 (17.19%) | 777 (31.91%) |  |
| **Smoking status, n (%)** |  |  | <0.001 |
| Current smoker | 357 (27.27%) | 733 (30.10%) |  |
| Ex-smoker | 649 (49.58%) | 941 (38.64%) |  |
| Never smoked | 303 (23.15%) | 761 (31.25%) |  |
| **Primary indication, n (%)** |  |  | <0.001 |
| Chronic non-cancer pain | 742 (56.68%) | 1,534 (63.00%) |  |
| Neuropathic pain | 256 (19.56%) | 344 (14.13%) |  |
| Hypermobility | 89 (6.80%) | 151 (6.20%) |  |
| Osteoarthritis | 75 (5.73%) | 100 (4.11%) |  |
| Inflammatory arthritis | 71 (5.42%) | 121 (4.97%) |  |
| Endometriosis | 34 (2.60%) | 67 (2.75%) |  |
| Cancer pain | 21 (1.60%) | 95 (3.90%) |  |
| Complex regional pain syndrome | 21 (1.60%) | 23 (0.94%) |  |
| **Charlson Comorbidity Index, mean ± SD** | 2.82 ± 3.49 | 2.65 ± 3.48 | 0.144 |
| **BPI-SF pain severity, mean ± SD** | 5.86 ± 1.70 | 6.04 ± 1.72 | 0.002 |
| **BPI-SF pain interference, mean ± SD** | 6.80 ± 2.16 | 6.91 ± 2.14 | 0.144 |
| **Pain VAS, mean ± SD** | 7.07 ± 2.00 | 7.16 ± 1.98 | 0.195 |
| **SF-MPQ-2 total, mean ± SD** | 4.77 ± 2.03 | 4.83 ± 2.03 | 0.456 |
| **EQ-5D-5L index value, mean ± SD** | 0.32 ± 0.31 | 0.29 ± 0.31 | 0.039 |
| **GAD-7, mean ± SD** | 8.28 ± 6.31 | 8.64 ± 6.49 | 0.099 |
| **SQS, mean ± SD** | 3.83 ± 2.33 | 3.79 ± 2.38 | 0.615 |
| **Baseline CBD dose (mg/day), mean ± SD** | 11.12 ± 10.40 | 12.37 ± 10.35 | <0.001 |
| **Baseline THC dose (mg/day), mean ± SD** | 15.76 ± 14.30 | 13.13 ± 15.46 | <0.001 |

**Supplementary Table 6. Cannabis-Based Medicinal Product (CBMP) Formulations and Cannabinoid Doses at Baseline, 1, 3, 6, 12, 18, and 24 Months.**

*CBD – cannabidiol; THC – (−)-trans-Δ^9^-tetrahydrocannabinol. * 5 most common administration routes at 24 months. All else aggregated into 'Other' category. † Before 12 months data for category aggregated into 'Other' category to prevent reidentification*

| **CBMP Details** | **Baseline** | **1 month** | **3 months** | **6 months** | **12 months** | **18 months** | **24 months** |
| --- | --- | --- | --- | --- | --- | --- | --- |
| **Cannabinoid Doses** |  |  |  |  |  |  |  |
| Daily CBD Dose (mg/day) | 15.00 [1.50-20.00] | 20.00 [10.69-20.55] | 20.00 [11.00-26.30] | 20.28 [11.00-31.55] | 20.28 [11.00-41.40] | 20.55 [11.00-49.50] | 20.53 [11.00-50.21] |
| Daily THC Dose (mg/day) | 19.00 [1.00-22.00] | 114.00 [7.10-121.00] | 115.50 [11.00-115.86] | 121.00 [16.00-220.00] | 125.00 [22.44-236.00] | 126.00 [25.00-242.00] | 126.50 [26.19-247.50] |
| **Administration Routes*** |  |  |  |  |  |  |  |
| Oil n (%) | 1,614 (43.11%) | 1,360 (36.32%) | 1,139 (30.42%) | 1,000 (26.71%) | 919  (24.55%) | 898  (23.99%) | 879  (23.48%) |
| Dried flower/flos n (%) | 383  (10.23%) | 338  (9.03%) | 385  (10.28%) | 535  (14.29%) | 718  (19.18%) | 778  (20.78%) | 801  (21.39%) |
| Dried flower/flos, Oil n (%) | 1,732 (46.26%) | 2,011 (53.71%) | 2,146 (57.32%) | 2,101 (56.12%) | 1,979 (52.86%) | 1,864 (49.79%) | 1,748 (46.69%) |
| Dried flower/flos, Pastille n (%)† |  |  |  |  | 4  (0.11%) | 21  (0.56%) | 77  (2.06%) |
| Dried flower/flos, Oil, Pastille n (%)† |  |  |  |  | 4  (0.11%) | 40  (1.07%) | 68  (1.82%) |
| Other n (%) | 15  (0.40%) | 19  (0.51%) | 31  (0.83%) | 32  (0.85%) | 34  (0.91%) | 41  (1.10%) | 53  (1.42%) |
| None n (%) | 0  (0.00%) | 16  (0.43%) | 41  (1.10%) | 70  (1.87%) | 74  (1.98%) | 80  (2.14%) | 82  (2.19%) |

**Supplementary Table 7. Comparison of Prescribed Analgesia between Baseline, 1, 3, 6, 12, 18, and 24 months**

Repeated measures analysis of variance to compare daily oral morphine equivalent and pregabalin equivalent in mg/day. For each outcome, sphericity assumptions were evaluated using Mauchly's test, with Greenhouse-Geisser corrections applied when sphericity was violated. SD – standard deviation

|  | **Mean ± SD** | | | | | | |  |
| --- | --- | --- | --- | --- | --- | --- | --- | --- |
|  | **Baseline** | **1 month** | **3 months** | **6 months** | **12 months** | **18 months** | **24 months** | **p-value** |
| Oral Morphine Equivalent (mg/day) | 33.09 ± 68.32 | 32.46 ± 67.49 | 31.16 ± 66.08 | 29.51 ± 63.75 | 27.65 ± 51.68 | 27.04 ± 51.36 | 26.77 ± 50.55 | <0.001 |
| Pregabalin Equivalent (mg/day) | 294.55 ± 255.42 | 292.73 ± 256.49 | 287.78 ± 253.10 | 281.96 ± 249.30 | 270.97 ± 232.24 | 271.51 ± 229.82 | 269.27 ± 230.32 | <0.001 |

**Supplementary Table 8. Pairwise Comparison of Prescribed Daily Oral Morphine Equivalent (mg/day)**

Post-hoc pairwise comparisons between time points were performed using paired t-tests with Bonferroni-Holm correction for multiple comparisons. Effect sizes were calculated using Cohen's d for paired differences. SD – standard deviation

| **Follow-up Interval** | **Mean Difference ± SD, p-value** | | | | | | |
| --- | --- | --- | --- | --- | --- | --- | --- |
|  | **Baseline** | **1 month** | **3 months** | **6 months** | **12 months** | **18 months** | **24 months** |
| **Baseline** |  |  |  |  |  |  |  |
| **1 month** | -0.63 ± 13.12 p=1.000 d=-0.05 |  |  |  |  |  |  |
| **3 months** | -1.93 ± 19.04 p=0.002 d=-0.10 | -1.30 ± 13.77 p=0.005 d=-0.09 |  |  |  |  |  |
| **6 months** | -3.58 ± 24.08 p<0.001 d=-0.15 | -2.95 ± 20.31 p<0.001 d=-0.15 | -1.64 ± 14.77 p<0.001 d=-0.11 |  |  |  |  |
| **12 months** | -5.44 ± 34.15 p<0.001 d=-0.16 | -4.81 ± 31.65 p<0.001 d=-0.15 | -3.51 ± 28.37 p<0.001 d=-0.12 | -1.86 ± 24.56 p=0.063 d=-0.08 |  |  |  |
| **18 months** | -6.05 ± 35.02 p<0.001 d=-0.17 | -5.43 ± 32.65 p<0.001 d=-0.17 | -4.12 ± 29.39 p<0.001 d=-0.14 | -2.48 ± 25.41 p=0.003 d=-0.10 | -0.61 ± 6.74 p=0.008 d=-0.09 |  |  |
| **24 months** | -6.32 ± 36.86 p<0.001 d=-0.17 | -5.69 ± 34.62 p<0.001 d=-0.16 | -4.39 ± 31.71 p<0.001 d=-0.14 | -2.74 ± 28.13 p=0.003 d=-0.10 | -0.88 ± 13.86 p=0.274 d=-0.06 | -0.27 ± 12.11 p=1.000 d=-0.02 |  |

**Supplementary Table 9. Proportion of Patients Reporting a Minimal Clinically Important Difference (MCID) in Daily Oral Morphine Equivalent (mg/day)**

|  | **n (%)** | | | | | |
| --- | --- | --- | --- | --- | --- | --- |
|  | **1 month** | **3 months** | **6 months** | **12 months** | **18 months** | **24 months** |
| **MCID in Oral Morphine Equivalent (mg/day)** | 44 (3.43%) | 109 (8.42%) | 182 (13.97%) | 248 (18.82%) | 286 (21.60%) | 302 (22.66%) |

**Supplementary Table 10. Pairwise Comparison of Prescribed Daily Pregabalin Equivalent (mg/day)**

Post-hoc pairwise comparisons between time points were performed using paired t-tests with Bonferroni-Holm correction for multiple comparisons. Effect sizes were calculated using Cohen's *d* for paired differences. *SD – standard deviation*

| **Follow-up Interval** | **Mean Difference ± SD, p-value** | | | | | | |
| --- | --- | --- | --- | --- | --- | --- | --- |
|  | **Baseline** | **1 month** | **3 months** | **6 months** | **12 months** | **18 months** | **24 months** |
| **Baseline** |  |  |  |  |  |  |  |
| **1 month** | -1.83 ± 42.03 p=1.000 d=-0.04 |  |  |  |  |  |  |
| **3 months** | -6.77 ± 78.91 p=0.333 d=-0.09 | -4.94 ± 68.28 p=0.875 d=-0.07 |  |  |  |  |  |
| **6 months** | -12.59 ± 115.77 p=0.047 d=-0.11 | -10.77 ± 109.17 p=0.117 d=-0.10 | -5.82 ± 85.51 p=1.000 d=-0.07 |  |  |  |  |
| **12 months** | -23.58 ± 150.04 p<0.001 d=-0.16 | -21.76 ± 145.33 p<0.001 d=-0.15 | -16.81 ± 123.14 p=0.003 d=-0.14 | -10.99 ± 92.60 p=0.018 d=-0.12 |  |  |  |
| **18 months** | -23.05 ± 153.17 p<0.001 d=-0.15 | -21.22 ± 148.56 p=0.001 d=-0.14 | -16.28 ± 126.91 p=0.007 d=-0.13 | -10.45 ± 97.52 p=0.055 d=-0.11 | 0.54 ± 40.51 p=1.000 d=0.01 |  |  |
| **24 months** | -25.28 ± 158.92 p<0.001 d=-0.16 | -23.46 ± 154.50 p<0.001 d=-0.15 | -18.51 ± 133.90 p=0.002 d=-0.14 | -12.69 ± 106.39 p=0.017 d=-0.12 | -1.70 ± 59.14 p=1.000 d=-0.03 | -2.24 ± 43.71 p=1.000 d=-0.05 |  |

**Supplementary Table 11. Frequency of Reported Adverse Events**

Adverse events graded in accordance with the Common Terminology Criteria for Adverse Events version 4.0. **% calculated a proportion of total case series (n=3,744).** Single occurrences were aggregated under ‘Other’ to avoid reidentification. *Grade 1 – Mild; Grade 2 – Moderate; Grade 3 – Severe; Grade 4 – Life-threatening/disabling*

| **Adverse Event** | **Grade 1** | **Grade 2** | **Grade 3** | **Grade 4** | **Total (%)** |
| --- | --- | --- | --- | --- | --- |
| Fatigue | 106 | 186 | 108 | 0 | 400 (10.68%) |
| Dry Mouth | 257 | 46 | 0 | 0 | 303 (8.09%) |
| Lethargy | 117 | 183 | 0 | 0 | 300 (8.01%) |
| Somnolence | 2 | 255 | 43 | 0 | 300 (8.01%) |
| Headache | 98 | 119 | 71 | 0 | 288 (7.69%) |
| Insomnia | 51 | 124 | 87 | 0 | 262 (7.00%) |
| Nausea | 152 | 91 | 11 | 0 | 254 (6.78%) |
| Dizziness | 94 | 96 | 39 | 0 | 229 (6.12%) |
| Concentration Impairment | 121 | 79 | 18 | 0 | 218 (5.82%) |
| Dyspepsia | 99 | 58 | 20 | 0 | 177 (4.73%) |
| Abdominal Pain | 73 | 71 | 24 | 0 | 168 (4.49%) |
| Constipation | 109 | 51 | 8 | 0 | 168 (4.49%) |
| Generalised Muscle Weakness | 42 | 53 | 52 | 4 | 151 (4.03%) |
| Vertigo | 68 | 55 | 23 | 0 | 146 (3.90%) |
| Cognitive Disturbance | 61 | 56 | 14 | 0 | 131 (3.50%) |
| Ataxia | 62 | 40 | 13 | 0 | 115 (3.07%) |
| Blurred Vision | 49 | 41 | 16 | 0 | 106 (2.83%) |
| Confusion | 61 | 28 | 12 | 0 | 101 (2.70%) |
| Anorexia | 34 | 55 | 10 | 0 | 99 (2.64%) |
| Pharyngitis | 1 | 75 | 5 | 0 | 81 (2.16%) |
| Weight Loss | 60 | 15 | 0 | 0 | 75 (2.00%) |
| Amnesia | 36 | 28 | 1 | 0 | 65 (1.74%) |
| Vomiting | 40 | 21 | 4 | 0 | 65 (1.74%) |
| Dysgeusia | 36 | 19 | 9 | 0 | 64 (1.71%) |
| Tremor | 34 | 23 | 7 | 0 | 64 (1.71%) |
| Delirium | 28 | 15 | 8 | 0 | 51 (1.36%) |
| Rash NOS | 26 | 21 | 3 | 0 | 50 (1.34%) |
| Spasticity | 13 | 15 | 19 | 0 | 47 (1.26%) |
| Fall | 22 | 18 | 4 | 0 | 44 (1.18%) |
| Urinary Tract Infection | 0 | 37 | 6 | 1 | 44 (1.18%) |
| Lung Infection | 0 | 36 | 7 | 0 | 43 (1.15%) |
| Diarrhoea | 9 | 23 | 8 | 1 | 41 (1.10%) |
| Fever | 25 | 10 | 3 | 0 | 38 (1.01%) |
| Anxiety | 5 | 10 | 9 | 0 | 24 (0.64%) |
| Pain | 2 | 5 | 10 | 0 | 17 (0.45%) |
| Depression | 3 | 6 | 2 | 0 | 11 (0.29%) |
| Palpitations | 2 | 6 | 0 | 0 | 8 (0.21%) |
| Paraesthesia | 0 | 5 | 3 | 0 | 8 (0.21%) |
| Increased Appetite | 3 | 3 | 1 | 0 | 7 (0.19%) |
| Movements Involuntary | 2 | 5 | 0 | 0 | 7 (0.19%) |
| Upper Respiratory Infection | 1 | 1 | 5 | 0 | 7 (0.19%) |
| Seizure | 0 | 4 | 2 | 0 | 6 (0.16%) |
| Arthralgia | 0 | 3 | 1 | 0 | 4 (0.11%) |
| Back Pain | 0 | 2 | 2 | 0 | 4 (0.11%) |
| Cough | 1 | 2 | 1 | 0 | 4 (0.11%) |
| Neuralgia | 1 | 1 | 2 | 0 | 4 (0.11%) |
| Pain In Extremity | 1 | 0 | 3 | 0 | 4 (0.11%) |
| Agitation | 0 | 3 | 0 | 0 | 3 (0.08%) |
| Allergic Reaction | 3 | 0 | 0 | 0 | 3 (0.08%) |
| Bloating | 1 | 2 | 0 | 0 | 3 (0.08%) |
| Dry Eye | 0 | 3 | 0 | 0 | 3 (0.08%) |
| Flatulence | 0 | 2 | 1 | 0 | 3 (0.08%) |
| Migraine | 0 | 2 | 1 | 0 | 3 (0.08%) |
| Myalgia | 0 | 3 | 0 | 0 | 3 (0.08%) |
| Nightmare | 0 | 1 | 2 | 0 | 3 (0.08%) |
| Pruritus | 0 | 2 | 1 | 0 | 3 (0.08%) |
| Psychosis | 1 | 0 | 2 | 0 | 3 (0.08%) |
| Sinus Tachycardia | 2 | 1 | 0 | 0 | 3 (0.08%) |
| Sore Throat | 1 | 1 | 1 | 0 | 3 (0.08%) |
| Urinary Tract Pain | 0 | 2 | 1 | 0 | 3 (0.08%) |
| Urinary Urgency | 0 | 2 | 1 | 0 | 3 (0.08%) |
| Allergic Rhinitis | 2 | 0 | 0 | 0 | 2 (0.05%) |
| Dyspnoea | 0 | 2 | 0 | 0 | 2 (0.05%) |
| Hot Flashes | 0 | 2 | 0 | 0 | 2 (0.05%) |
| Non Cardiac Chest Pain | 0 | 0 | 2 | 0 | 2 (0.05%) |
| Tinnitus | 1 | 1 | 0 | 0 | 2 (0.05%) |
| Vasovagal Reaction | 0 | 0 | 1 | 1 | 2 (0.05%) |
| Weight Gain | 0 | 1 | 1 | 0 | 2 (0.05%) |
| **Other (single occurrences)** | 9 | 17 | 12 | 1 | 39 (1.04%) |
| **Total** | **2,027** | **2,143** | **720** | **8** | **4,898** |

**Supplementary Table 12. Univariable Logistic Regression for Reporting One or More Adverse Events Across 24 Months.**

*BMI – body mass index; CBD – cannabidiol; CBMPs – cannabis-based medicinal products; CI – confidence interval; GAD-7 – Generalised Anxiety Disorder-7; Max – maximum; Min – minimum; OR – odds ratio; Q1 – quartile 1; Q3 – quartile 3; SQS – Single-Item Sleep Quality Scale; THC – (-)-trans-Δ9-tetrahydrocannabinol.*

| **Variable** | **n** | **OR (95% CI)** | **p-value** |
| --- | --- | --- | --- |
| **Primary Diagnosis** |  |  |  |
| Chronic Non-Cancer Pain | 2,276 | Ref. | - |
| Cancer pain | 116 | 0.30 (0.10-0.66) | 0.009 |
| Complex regional pain syndrome | 44 | 1.47 (0.63-3.03) | 0.331 |
| Endometriosis | 101 | 1.43 (0.82-2.37) | 0.178 |
| Hypermobility | 240 | 2.20 (1.60-3.01) | <0.001 |
| Inflammatory arthritis | 192 | 1.08 (0.69-1.63) | 0.716 |
| Neuropathic pain | 600 | 1.24 (0.96-1.59) | 0.088 |
| Osteoarthritis | 175 | 1.15 (0.73-1.75) | 0.518 |
| **Age (Years)** |  |  |  |
| 18-30 | 416 | Ref. | - |
| 31-40 | 1,007 | 1.05 (0.74-1.51) | 0.800 |
| 41-50 | 1,027 | 1.07 (0.76-1.54) | 0.700 |
| 51-60 | 728 | 1.50 (1.05-2.16) | 0.027 |
| 61+ | 566 | 2.15 (1.51-3.11) | <0.001 |
| **Sex** |  |  |  |
| Male | 2,172 | Ref. | - |
| Female | 1,572 | 2.06 (1.71-2.48) | <0.001 |
| **BMI (kg/m^2^)** |  |  |  |
| 20-24.99 | 1,093 | Ref. | - |
| <20 | 296 | 1.38 (0.98-1.93) | 0.062 |
| 25-29.99 | 1,165 | 0.97 (0.76-1.23) | 0.805 |
| 30-34.99 | 551 | 1.09 (0.81-1.45) | 0.564 |
| 35+ | 455 | 0.94 (0.68-1.28) | 0.689 |
| **Cannabis Status** |  |  |  |
| Never Consumed Cannabis | 1,002 | Ref. | - |
| Former Cannabis Consumer | 590 | 0.62 (0.48-0.80) | <0.001 |
| Current Cannabis Consumer | 2,152 | 0.33 (0.27-0.41) | <0.001 |

| **CBMPs at 24 Months** |  |  |  |
| --- | --- | --- | --- |
| Oil | 879 | Ref. | - |
| Dried flower/flos | 801 | 0.64 (0.49-0.82) | <0.001 |
| Dried flower/flos, Oil | 1,748 | 0.50 (0.40-0.63) | <0.001 |
| Dried flower/flos, Pastille | 77 | 0.52 (0.24-1.00) | 0.071 |
| Dried flower/flos, Oil, Pastille | 68 | 0.31 (0.11-0.71) | 0.013 |
| Other | 89 | 0.67 (0.35-1.19) | 0.197 |
| None | 82 | 1.35 (0.78-2.24) | 0.265 |
| **Daily CBD Dose (mg/day) at 24 Months** |  |  |  |
| Min to Q1 | 962 | Ref. | - |
| Q1 to Median | 914 | 0.99 (0.75-1.31) | 0.926 |
| Median to Q3 | 932 | 1.23 (0.94-1.60) | 0.136 |
| Q3 to Max | 936 | 1.78 (1.38-2.29) | <0.001 |
| **Daily THC Dose (mg/day) at 24 Months** |  |  |  |
| Min to Q1 | 936 | Ref. | - |
| Q1 to Median | 942 | 0.53 (0.41-0.68) | <0.001 |
| Median to Q3 | 949 | 0.58 (0.45-0.74) | <0.001 |
| Q3 to Max | 917 | 0.45 (0.34-0.58) | <0.001 |
| **SQS at Baseline** |  |  |  |
| 7-10 | 558 | Ref. | - |
| 4-6 | 1,213 | 1.01 (0.76-1.36) | 0.919 |
| 0-3 | 1,970 | 1.04 (0.80-1.36) | 0.795 |
| **GAD-7 at Baseline** |  |  |  |
| <10 | 2,245 | Ref. | - |
| 10-14 | 683 | 1.01 (0.79-1.28) | 0.945 |
| 15+ | 817 | 0.78 (0.61-0.99) | 0.046 |
| **Prescribed Opioids at Baseline** |  |  |  |
| No | 2,300 | Ref. | - |
| Yes | 1,444 | 1.41 (1.17-1.70) | <0.001 |
| **Prescribed Gabapentinoids at Baseline** |  |  |  |
| No | 3,020 | Ref. | - |
| Yes | 724 | 1.42 (1.14-1.76) | 0.002 |

**Supplementary Table 13. Multivariable Logistic Regression for Reporting One or More Adverse Events Across 24 Months.**

*BMI – body mass index; CBD – cannabidiol; CBMPs – cannabis-based medicinal products; CI – confidence interval; GAD-7 – Generalised Anxiety Disorder-7; Max – maximum; Min – minimum; OR – odds ratio; Q1 – quartile 1; Q3 – quartile 3; SQS – Single-Item Sleep Quality Scale; THC – (−)-trans-Δ^9^-tetrahydrocannabinol.*

| **Variable** | **n** | **OR (95% CI)** | **p-value** |
| --- | --- | --- | --- |
| **Primary Diagnosis** |  |  |  |
| Chronic Non-Cancer Pain | 2,276 | Ref. | - |
| Cancer pain | 110 | 0.19 (0.06-0.42) | <0.001 |
| Complex regional pain syndrome | 42 | 1.21 (0.48-2.69) | 0.654 |
| Endometriosis | 97 | 1.12 (0.61-1.96) | 0.701 |
| Hypermobility | 228 | 1.84 (1.27-2.62) | <0.001 |
| Inflammatory arthritis | 187 | 0.96 (0.60-1.48) | 0.849 |
| Neuropathic pain | 576 | 1.13 (0.86-1.47) | 0.374 |
| Osteoarthritis | 167 | 0.86 (0.53-1.35) | 0.523 |
| **Age (Years)** |  |  |  |
| 18-30 | 393 | Ref. | - |
| 31-40 | 969 | 1.32 (0.90-1.95) | 0.158 |
| 41-50 | 990 | 1.35 (0.93-2.01) | 0.125 |
| 51-60 | 697 | 1.78 (1.20-2.66) | 0.004 |
| 61+ | 511 | 2.18 (1.44-3.35) | <0.001 |
| **Sex** |  |  |  |
| Male | 2,077 | Ref. | - |
| Female | 1,483 | 1.53 (1.23-1.91) | <0.001 |
| **BMI (kg/m^2^)** |  |  |  |
| 20-24.99 | 1,093 | Ref. | - |
| <20 | 296 | 1.35 (0.94-1.92) | 0.104 |
| 25-29.99 | 1,165 | 0.98 (0.76-1.26) | 0.874 |
| 30-34.99 | 551 | 0.97 (0.71-1.30) | 0.832 |
| 35+ | 455 | 0.74 (0.53-1.04) | 0.087 |
| **Cannabis Status** |  |  |  |
| Never Consumed Cannabis | 923 | Ref. | - |
| Former Cannabis Consumer | 571 | 0.82 (0.61-1.11) | 0.200 |
| Current Cannabis Consumer | 2,066 | 0.47 (0.36-0.62) | <0.001 |

| **CBMPs at 24 Months** |  |  |  |
| --- | --- | --- | --- |
| Oil | 821 | Ref. | - |
| Dried flower/flos | 777 | 1.48 (0.77-2.94) | 0.247 |
| Dried flower/flos, Oil | 1,667 | 1.00 (0.54-1.93) | 0.993 |
| Dried flower/flos, Pastille | 76 | 1.10 (0.40-2.83) | 0.849 |
| Dried flower/flos, Oil, Pastille | 68 | 0.46 (0.14-1.34) | 0.176 |
| Other | 84 | 0.73 (0.33-1.51) | 0.415 |
| None | 67 | 2.61 (1.36-4.89) | 0.003 |
| **Daily CBD Dose (mg/day) at 24 Months** |  |  |  |
| Min to Q1 | 898 | Ref. | - |
| Q1 to Median | 863 | 1.00 (0.72-1.39) | 1.000 |
| Median to Q3 | 888 | 1.37 (0.99-1.88) | 0.055 |
| Q3 to Max | 911 | 1.81 (1.35-2.45) | <0.001 |
| **Daily THC Dose (mg/day) at 24 Months** |  |  |  |
| Min to Q1 | 863 | Ref. | - |
| Q1 to Median | 879 | 0.72 (0.38-1.33) | 0.308 |
| Median to Q3 | 916 | 0.82 (0.41-1.58) | 0.558 |
| Q3 to Max | 902 | 0.73 (0.36-1.43) | 0.361 |
| **SQS at Baseline** |  |  |  |
| 7-10 | 533 | Ref. | - |
| 4-6 | 1,155 | 1.06 (0.78-1.46) | 0.697 |
| 0-3 | 1,872 | 1.05 (0.78-1.42) | 0.762 |
| **GAD-7 at Baseline** |  |  |  |
| <10 | 2,139 | Ref. | - |
| 10-14 | 649 | 1.05 (0.80-1.36) | 0.724 |
| 15+ | 772 | 0.84 (0.64-1.10) | 0.206 |
| **Prescribed Opioids at Baseline** |  |  |  |
| No | 2,177 | Ref. | - |
| Yes | 1,383 | 1.22 (1.00-1.49) | 0.055 |
| **Prescribed Gabapentinoids at Baseline** |  |  |  |
| No | 2,876 | Ref. | - |
| Yes | 684 | 1.37 (1.08-1.74) | 0.010 |

**Supplementary Table 14. Pairwise Comparison of EuroQol 5-Dimension 5-Level (EQ-5D-5L) Mobility**
*Post-hoc pairwise comparisons between time points were performed using paired t-tests with Bonferroni-Holm correction for multiple comparisons. Effect sizes were calculated using Cohen's d for paired differences. SD – standard deviation*

| **Follow-up Interval** | **Mean Difference ± SD, p-value** | | | | | | |
| --- | --- | --- | --- | --- | --- | --- | --- |
|  | **Baseline** | **1 month** | **3 months** | **6 months** | **12 months** | **18 months** | **24 months** |
| **Baseline** |  |  |  |  |  |  |  |
| **1 month** | -0.28 ± 0.89 p<0.001 d=-0.32 |  |  |  |  |  |  |
| **3 months** | -0.31 ± 0.92 p<0.001 d=-0.33 | -0.03 ± 0.81 p=1.000 d=-0.03 |  |  |  |  |  |
| **6 months** | -0.36 ± 0.99 p<0.001 d=-0.36 | -0.07 ± 0.88 p<0.001 d=-0.08 | -0.05 ± 0.80 p=0.005 d=-0.06 |  |  |  |  |
| **12 months** | -0.37 ± 1.01 p<0.001 d=-0.37 | -0.09 ± 0.89 p<0.001 d=-0.10 | -0.07 ± 0.85 p<0.001 d=-0.08 | -0.02 ± 0.82 p=1.000 d=-0.02 |  |  |  |
| **18 months** | -0.40 ± 1.04 p<0.001 d=-0.39 | -0.12 ± 0.94 p<0.001 d=-0.13 | -0.09 ± 0.89 p<0.001 d=-0.11 | -0.05 ± 0.89 p=0.030 d=-0.05 | -0.03 ± 0.79 p=0.684 d=-0.03 |  |  |
| **24 months** | -0.34 ± 1.05 p<0.001 d=-0.32 | -0.05 ± 0.98 p=0.017 d=-0.05 | -0.03 ± 0.94 p=1.000 d=-0.03 | 0.02 ± 0.88 p=1.000 d=0.02 | 0.04 ± 0.85 p=0.112 d=0.05 | 0.07 ± 0.83 p<0.001 d=0.08 |  |

**Supplementary Table 15. Pairwise Comparison of EuroQol 5-Dimension 5-Level (EQ-5D-5L) Self-Care**

*Post-hoc pairwise comparisons between time points were performed using paired t-tests with Bonferroni-Holm correction for multiple comparisons. Effect sizes were calculated using Cohen's d for paired differences. SD – standard deviation*

| **Follow-up Interval** | **Mean Difference ± SD, p-value** | | | | | | |
| --- | --- | --- | --- | --- | --- | --- | --- |
|  | **Baseline** | **1 month** | **3 months** | **6 months** | **12 months** | **18 months** | **24 months** |
| **Baseline** |  |  |  |  |  |  |  |
| **1 month** | -0.16 ± 0.83 p<0.001 d=-0.19 |  |  |  |  |  |  |
| **3 months** | -0.18 ± 0.88 p<0.001 d=-0.21 | -0.03 ± 0.79 p=0.857 d=-0.03 |  |  |  |  |  |
| **6 months** | -0.22 ± 0.95 p<0.001 d=-0.23 | -0.06 ± 0.84 p<0.001 d=-0.07 | -0.03 ± 0.78 p=0.276 d=-0.04 |  |  |  |  |
| **12 months** | -0.20 ± 0.97 p<0.001 d=-0.21 | -0.04 ± 0.86 p=0.086 d=-0.05 | -0.01 ± 0.84 p=1.000 d=-0.02 | 0.02 ± 0.84 p=1.000 d=0.02 |  |  |  |
| **18 months** | -0.22 ± 0.93 p<0.001 d=-0.24 | -0.07 ± 0.85 p<0.001 d=-0.08 | -0.04 ± 0.81 p=0.059 d=-0.05 | -0.01 ± 0.82 p=1.000 d=-0.01 | -0.03 ± 0.76 p=0.833 d=-0.03 |  |  |
| **24 months** | -0.19 ± 0.99 p<0.001 d=-0.20 | -0.04 ± 0.93 p=0.353 d=-0.04 | -0.01 ± 0.88 p=1.000 d=-0.01 | 0.02 ± 0.93 p=1.000 d=0.02 | 0.00 ± 0.82 p=1.000 d=0.01 | 0.03 ± 0.78 p=0.406 d=0.04 |  |

**Supplementary Table 16. Pairwise Comparison of EuroQol 5-Dimension 5-Level (EQ-5D-5L) Usual Activities**

*Post-hoc pairwise comparisons between time points were performed using paired t-tests with Bonferroni-Holm correction for multiple comparisons. Effect sizes were calculated using Cohen's d for paired differences. SD – standard deviation*

| **Follow-up Interval** | **Mean Difference ± SD, p-value** | | | | | | |
| --- | --- | --- | --- | --- | --- | --- | --- |
|  | **Baseline** | **1 month** | **3 months** | **6 months** | **12 months** | **18 months** | **24 months** |
| **Baseline** |  |  |  |  |  |  |  |
| **1 month** | -0.50 ± 1.05 p<0.001 d=-0.48 |  |  |  |  |  |  |
| **3 months** | -0.52 ± 1.08 p<0.001 d=-0.48 | -0.02 ± 0.96 p=1.000 d=-0.02 |  |  |  |  |  |
| **6 months** | -0.59 ± 1.13 p<0.001 d=-0.52 | -0.10 ± 0.99 p<0.001 d=-0.10 | -0.07 ± 0.90 p<0.001 d=-0.08 |  |  |  |  |
| **12 months** | -0.58 ± 1.13 p<0.001 d=-0.51 | -0.08 ± 1.01 p<0.001 d=-0.08 | -0.06 ± 0.97 p=0.006 d=-0.06 | 0.01 ± 0.93 p=1.000 d=0.02 |  |  |  |
| **18 months** | -0.60 ± 1.18 p<0.001 d=-0.51 | -0.11 ± 1.11 p<0.001 d=-0.10 | -0.08 ± 1.06 p<0.001 d=-0.08 | -0.01 ± 1.03 p=1.000 d=-0.01 | -0.03 ± 0.93 p=1.000 d=-0.03 |  |  |
| **24 months** | -0.61 ± 1.20 p<0.001 d=-0.51 | -0.12 ± 1.11 p<0.001 d=-0.11 | -0.09 ± 1.07 p<0.001 d=-0.09 | -0.02 ± 1.00 p=1.000 d=-0.02 | -0.04 ± 0.96 p=0.451 d=-0.04 | -0.01 ± 0.91 p=1.000 d=-0.01 |  |

**Supplementary Table 17. Pairwise Comparison of EuroQol 5-Dimension 5-Level (EQ-5D-5L) Pain / Discomfort**

*Post-hoc pairwise comparisons between time points were performed using paired t-tests with Bonferroni-Holm correction for multiple comparisons. Effect sizes were calculated using Cohen's d for paired differences. SD – standard deviation*

| **Follow-up Interval** | **Mean Difference ± SD, p-value** | | | | | | |
| --- | --- | --- | --- | --- | --- | --- | --- |
|  | **Baseline** | **1 month** | **3 months** | **6 months** | **12 months** | **18 months** | **24 months** |
| **Baseline** |  |  |  |  |  |  |  |
| **1 month** | -0.75 ± 0.98 p<0.001 d=-0.76 |  |  |  |  |  |  |
| **3 months** | -0.86 ± 1.00 p<0.001 d=-0.86 | -0.11 ± 0.89 p<0.001 d=-0.13 |  |  |  |  |  |
| **6 months** | -0.91 ± 1.01 p<0.001 d=-0.89 | -0.16 ± 0.93 p<0.001 d=-0.17 | -0.04 ± 0.85 p=0.043 d=-0.05 |  |  |  |  |
| **12 months** | -0.88 ± 1.05 p<0.001 d=-0.84 | -0.13 ± 1.00 p<0.001 d=-0.13 | -0.02 ± 0.90 p=1.000 d=-0.02 | 0.02 ± 0.87 p=1.000 d=0.03 |  |  |  |
| **18 months** | -0.91 ± 1.08 p<0.001 d=-0.84 | -0.16 ± 1.06 p<0.001 d=-0.15 | -0.04 ± 0.97 p=0.162 d=-0.04 | 0.00 ± 0.95 p=1.000 d=0.00 | -0.02 ± 0.88 p=1.000 d=-0.03 |  |  |
| **24 months** | -0.94 ± 1.05 p<0.001 d=-0.90 | -0.19 ± 1.04 p<0.001 d=-0.19 | -0.08 ± 1.00 p<0.001 d=-0.08 | -0.03 ± 0.97 p=0.563 d=-0.04 | -0.06 ± 0.90 p=0.001 d=-0.07 | -0.04 ± 0.88 p=0.292 d=-0.04 |  |

**Supplementary Table 18. Pairwise Comparison of EuroQol 5-Dimension 5-Level (EQ-5D-5L) Anxiety / Depression**

*Post-hoc pairwise comparisons between time points were performed using paired t-tests with Bonferroni-Holm correction for multiple comparisons. Effect sizes were calculated using Cohen's d for paired differences. SD – standard deviation*

| **Follow-up Interval** | **Mean Difference ± SD, p-value** | | | | | | |
| --- | --- | --- | --- | --- | --- | --- | --- |
|  | **Baseline** | **1 month** | **3 months** | **6 months** | **12 months** | **18 months** | **24 months** |
| **Baseline** |  |  |  |  |  |  |  |
| **1 month** | -0.41 ± 0.99 p<0.001 d=-0.41 |  |  |  |  |  |  |
| **3 months** | -0.43 ± 1.06 p<0.001 d=-0.41 | -0.03 ± 0.85 p=1.000 d=-0.03 |  |  |  |  |  |
| **6 months** | -0.46 ± 1.13 p<0.001 d=-0.41 | -0.05 ± 0.94 p=0.008 d=-0.06 | -0.03 ± 0.87 p=1.000 d=-0.03 |  |  |  |  |
| **12 months** | -0.46 ± 1.20 p<0.001 d=-0.38 | -0.06 ± 1.03 p=0.021 d=-0.05 | -0.03 ± 0.96 p=1.000 d=-0.03 | -0.00 ± 0.90 p=1.000 d=-0.00 |  |  |  |
| **18 months** | -0.49 ± 1.23 p<0.001 d=-0.40 | -0.09 ± 1.06 p<0.001 d=-0.08 | -0.06 ± 1.02 p=0.007 d=-0.06 | -0.03 ± 0.99 p=1.000 d=-0.03 | -0.03 ± 0.96 p=1.000 d=-0.03 |  |  |
| **24 months** | -0.53 ± 1.22 p<0.001 d=-0.44 | -0.13 ± 1.07 p<0.001 d=-0.12 | -0.10 ± 1.01 p<0.001 d=-0.10 | -0.07 ± 1.00 p<0.001 d=-0.07 | -0.07 ± 0.99 p<0.001 d=-0.07 | -0.04 ± 0.93 p=0.176 d=-0.04 |  |

**Supplementary Table 19. Pairwise Comparison of EuroQol 5-Dimension 5-Level (EQ-5D-5L) Index Value**

*Post-hoc pairwise comparisons between time points were performed using paired t-tests with Bonferroni-Holm correction for multiple comparisons. Effect sizes were calculated using Cohen's d for paired differences. SD – standard deviation*

| **Follow-up Interval** | **Mean Difference ± SD, p-value** | | | | | | |
| --- | --- | --- | --- | --- | --- | --- | --- |
|  | **Baseline** | **1 month** | **3 months** | **6 months** | **12 months** | **18 months** | **24 months** |
| **Baseline** |  |  |  |  |  |  |  |
| **1 month** | 0.19 ± 0.27 p<0.001 d=0.69 |  |  |  |  |  |  |
| **3 months** | 0.21 ± 0.28 p<0.001 d=0.73 | 0.02 ± 0.22 p<0.001 d=0.09 |  |  |  |  |  |
| **6 months** | 0.22 ± 0.29 p<0.001 d=0.75 | 0.03 ± 0.24 p<0.001 d=0.14 | 0.01 ± 0.21 p=0.001 d=0.07 |  |  |  |  |
| **12 months** | 0.22 ± 0.31 p<0.001 d=0.73 | 0.04 ± 0.26 p<0.001 d=0.15 | 0.02 ± 0.24 p<0.001 d=0.08 | 0.01 ± 0.22 p=1.000 d=0.02 |  |  |  |
| **18 months** | 0.23 ± 0.31 p<0.001 d=0.74 | 0.04 ± 0.27 p<0.001 d=0.16 | 0.02 ± 0.25 p<0.001 d=0.09 | 0.01 ± 0.24 p=0.311 d=0.04 | 0.00 ± 0.22 p=1.000 d=0.02 |  |  |
| **24 months** | 0.23 ± 0.31 p<0.001 d=0.76 | 0.05 ± 0.27 p<0.001 d=0.18 | 0.03 ± 0.25 p<0.001 d=0.11 | 0.01 ± 0.24 p=0.012 d=0.06 | 0.01 ± 0.22 p=0.513 d=0.04 | 0.00 ± 0.21 p=1.000 d=0.02 |  |

**Supplementary Table 20. Pairwise Comparison of Generalised Anxiety Disorder-7**

*Post-hoc pairwise comparisons between time points were performed using paired t-tests with Bonferroni-Holm correction for multiple comparisons. Effect sizes were calculated using Cohen's d for paired differences. SD – standard deviation*

| **Follow-up Interval** | **Mean Difference ± SD, p-value** | | | | | | |
| --- | --- | --- | --- | --- | --- | --- | --- |
|  | **Baseline** | **1 month** | **3 months** | **6 months** | **12 months** | **18 months** | **24 months** |
| **Baseline** |  |  |  |  |  |  |  |
| **1 month** | -2.96 ± 5.42 p<0.001 d=-0.55 |  |  |  |  |  |  |
| **3 months** | -2.91 ± 5.74 p<0.001 d=-0.51 | 0.05 ± 3.96 p=1.000 d=0.01 |  |  |  |  |  |
| **6 months** | -3.09 ± 5.95 p<0.001 d=-0.52 | -0.13 ± 4.26 p=1.000 d=-0.03 | -0.18 ± 3.79 p=0.092 d=-0.05 |  |  |  |  |
| **12 months** | -3.22 ± 6.31 p<0.001 d=-0.51 | -0.26 ± 4.80 p=0.017 d=-0.05 | -0.31 ± 4.58 p<0.001 d=-0.07 | -0.13 ± 4.16 p=1.000 d=-0.03 |  |  |  |
| **18 months** | -3.22 ± 6.26 p<0.001 d=-0.51 | -0.26 ± 4.86 p=0.019 d=-0.05 | -0.31 ± 4.67 p<0.001 d=-0.07 | -0.13 ± 4.40 p=1.000 d=-0.03 | -0.00 ± 4.40 p=1.000 d=-0.00 |  |  |
| **24 months** | -3.68 ± 6.25 p<0.001 d=-0.59 | -0.72 ± 4.94 p<0.001 d=-0.15 | -0.77 ± 4.57 p<0.001 d=-0.17 | -0.59 ± 4.43 p<0.001 d=-0.13 | -0.46 ± 4.11 p<0.001 d=-0.11 | -0.46 ± 4.17 p<0.001 d=-0.11 |  |

**Supplementary Table 21. Pairwise Comparison of Single-Item Sleep Quality Scale**

*Post-hoc pairwise comparisons between time points were performed using paired t-tests with Bonferroni-Holm correction for multiple comparisons. Effect sizes were calculated using Cohen's d for paired differences. SD – standard deviation*

| **Follow-up Interval** | **Mean Difference ± SD, p-value** | | | | | | |
| --- | --- | --- | --- | --- | --- | --- | --- |
|  | **Baseline** | **1 month** | **3 months** | **6 months** | **12 months** | **18 months** | **24 months** |
| **Baseline** |  |  |  |  |  |  |  |
| **1 month** | 1.73 ± 2.71 p<0.001 d=0.64 |  |  |  |  |  |  |
| **3 months** | 1.76 ± 2.80 p<0.001 d=0.63 | 0.04 ± 2.41 p=1.000 d=0.02 |  |  |  |  |  |
| **6 months** | 1.80 ± 2.79 p<0.001 d=0.64 | 0.07 ± 2.60 p=1.000 d=0.03 | 0.03 ± 2.34 p=1.000 d=0.01 |  |  |  |  |
| **12 months** | 1.80 ± 2.75 p<0.001 d=0.65 | 0.07 ± 2.75 p=1.000 d=0.03 | 0.03 ± 2.60 p=1.000 d=0.01 | 0.00 ± 2.38 p=1.000 d=0.00 |  |  |  |
| **18 months** | 1.88 ± 2.98 p<0.001 d=0.63 | 0.15 ± 2.78 p=0.018 d=0.05 | 0.11 ± 2.75 p=0.245 d=0.04 | 0.08 ± 2.49 p=0.965 d=0.03 | 0.08 ± 2.41 p=0.989 d=0.03 |  |  |
| **24 months** | 1.92 ± 2.91 p<0.001 d=0.66 | 0.20 ± 2.84 p<0.001 d=0.07 | 0.16 ± 2.76 p=0.010 d=0.06 | 0.13 ± 2.58 p=0.058 d=0.05 | 0.12 ± 2.49 p=0.052 d=0.05 | 0.05 ± 2.36 p=1.000 d=0.02 |  |

**Supplementary Table 22. Pairwise Comparison of Patient Global Impression of Change**

*Post-hoc pairwise comparisons between time points were performed using paired t-tests with Bonferroni-Holm correction for multiple comparisons. Effect sizes were calculated using Cohen's d for paired differences. SD – standard deviation*

| **Follow-up Interval** | **Mean Difference ± SD, p-value** | | | | | |
| --- | --- | --- | --- | --- | --- | --- |
|  | **1 month** | **3 months** | **6 months** | **12 months** | **18 months** | **24 months** |
| **1 month** |  |  |  |  |  |  |
| **3 months** | 0.32 ± 1.30 p<0.001 d=0.25 |  |  |  |  |  |
| **6 months** | 0.51 ± 1.43 p<0.001 d=0.36 | 0.19 ± 1.13 p<0.001 d=0.16 |  |  |  |  |
| **12 months** | 0.61 ± 1.52 p<0.001 d=0.40 | 0.28 ± 1.29 p<0.001 d=0.22 | 0.10 ± 1.12 p<0.001 d=0.09 |  |  |  |
| **18 months** | 0.61 ± 1.54 p<0.001 d=0.40 | 0.29 ± 1.35 p<0.001 d=0.21 | 0.10 ± 1.22 p<0.001 d=0.08 | 0.00 ± 1.15 p=1.000 d=0.00 |  |  |
| **24 months** | 0.84 ± 1.55 p<0.001 d=0.54 | 0.52 ± 1.34 p<0.001 d=0.38 | 0.33 ± 1.23 p<0.001 d=0.27 | 0.23 ± 1.10 p<0.001 d=0.21 | 0.23 ± 1.09 p<0.001 d=0.21 |  |

**Supplementary Table 23. Pairwise Comparison of Brief Pain Inventory Short Form (BPI-SF) Pain Severity**

*Post-hoc pairwise comparisons between time points were performed using paired t-tests with Bonferroni-Holm correction for multiple comparisons. Effect sizes were calculated using Cohen's d for paired differences. SD – standard deviation*

| **Follow-up Interval** | **Mean Difference ± SD, p-value** | | | | | | |
| --- | --- | --- | --- | --- | --- | --- | --- |
|  | **Baseline** | **1 month** | **3 months** | **6 months** | **12 months** | **18 months** | **24 months** |
| **Baseline** |  |  |  |  |  |  |  |
| **1 month** | -0.91 ± 1.73 p<0.001 d=-0.53 |  |  |  |  |  |  |
| **3 months** | -1.10 ± 1.74 p<0.001 d=-0.63 | -0.19 ± 1.56 p<0.001 d=-0.12 |  |  |  |  |  |
| **6 months** | -1.22 ± 1.86 p<0.001 d=-0.65 | -0.30 ± 1.68 p<0.001 d=-0.18 | -0.11 ± 1.57 p<0.001 d=-0.07 |  |  |  |  |
| **12 months** | -1.21 ± 1.87 p<0.001 d=-0.65 | -0.30 ± 1.75 p<0.001 d=-0.17 | -0.10 ± 1.63 p=0.002 d=-0.06 | 0.01 ± 1.56 p=1.000 d=0.00 |  |  |  |
| **18 months** | -1.24 ± 1.99 p<0.001 d=-0.63 | -0.33 ± 1.93 p<0.001 d=-0.17 | -0.14 ± 1.87 p<0.001 d=-0.08 | -0.03 ± 1.73 p=1.000 d=-0.02 | -0.04 ± 1.56 p=1.000 d=-0.02 |  |  |
| **24 months** | -1.12 ± 1.97 p<0.001 d=-0.57 | -0.20 ± 1.92 p<0.001 d=-0.11 | -0.01 ± 1.84 p=1.000 d=-0.01 | 0.10 ± 1.78 p=0.015 d=0.06 | 0.09 ± 1.64 p=0.013 d=0.06 | 0.13 ± 1.55 p<0.001 d=0.08 |  |

**Supplementary Table 24. Pairwise Comparison of Brief Pain Inventory Short Form (BPI-SF) Pain Interference**

*Post-hoc pairwise comparisons between time points were performed using paired t-tests with Bonferroni-Holm correction for multiple comparisons. Effect sizes were calculated using Cohen's d for paired differences. SD – standard deviation*

| **Follow-up Interval** | **Mean Difference ± SD, p-value** | | | | | | |
| --- | --- | --- | --- | --- | --- | --- | --- |
|  | **Baseline** | **1 month** | **3 months** | **6 months** | **12 months** | **18 months** | **24 months** |
| **Baseline** |  |  |  |  |  |  |  |
| **1 month** | -1.32 ± 2.18 p<0.001 d=-0.60 |  |  |  |  |  |  |
| **3 months** | -1.55 ± 2.22 p<0.001 d=-0.70 | -0.24 ± 2.01 p<0.001 d=-0.12 |  |  |  |  |  |
| **6 months** | -1.75 ± 2.36 p<0.001 d=-0.74 | -0.43 ± 2.23 p<0.001 d=-0.19 | -0.20 ± 1.97 p<0.001 d=-0.10 |  |  |  |  |
| **12 months** | -1.76 ± 2.35 p<0.001 d=-0.75 | -0.45 ± 2.31 p<0.001 d=-0.19 | -0.21 ± 2.13 p<0.001 d=-0.10 | -0.01 ± 2.01 p=1.000 d=-0.01 |  |  |  |
| **18 months** | -1.55 ± 2.47 p<0.001 d=-0.63 | -0.23 ± 2.47 p<0.001 d=-0.09 | 0.01 ± 2.36 p=1.000 d=0.00 | 0.20 ± 2.19 p<0.001 d=0.09 | 0.22 ± 1.98 p<0.001 d=0.11 |  |  |
| **24 months** | -1.49 ± 2.52 p<0.001 d=-0.59 | -0.17 ± 2.43 p<0.001 d=-0.07 | 0.06 ± 2.33 p=1.000 d=0.03 | 0.26 ± 2.25 p<0.001 d=0.11 | 0.27 ± 2.12 p<0.001 d=0.13 | 0.06 ± 2.04 p=1.000 d=0.03 |  |

**Supplementary Table 25. Pairwise Comparison of Short-Form McGill Pain Questionnaire-2 (SF-MPQ-2) Total**

*Post-hoc pairwise comparisons between time points were performed using paired t-tests with Bonferroni-Holm correction for multiple comparisons. Effect sizes were calculated using Cohen's d for paired differences. SD – standard deviation*

| **Follow-up Interval** | **Mean Difference ± SD, p-value** | | | | | | |
| --- | --- | --- | --- | --- | --- | --- | --- |
|  | **Baseline** | **1 month** | **3 months** | **6 months** | **12 months** | **18 months** | **24 months** |
| **Baseline** |  |  |  |  |  |  |  |
| **1 month** | -0.94 ± 1.80 p<0.001 d=-0.52 |  |  |  |  |  |  |
| **3 months** | -1.26 ± 1.88 p<0.001 d=-0.67 | -0.32 ± 1.64 p<0.001 d=-0.19 |  |  |  |  |  |
| **6 months** | -1.25 ± 2.03 p<0.001 d=-0.62 | -0.32 ± 1.86 p<0.001 d=-0.17 | 0.00 ± 1.63 p=1.000 d=0.00 |  |  |  |  |
| **12 months** | -1.07 ± 2.17 p<0.001 d=-0.49 | -0.13 ± 2.07 p=0.003 d=-0.06 | 0.19 ± 2.04 p<0.001 d=0.09 | 0.19 ± 1.84 p<0.001 d=0.10 |  |  |  |
| **18 months** | -1.11 ± 2.18 p<0.001 d=-0.51 | -0.17 ± 2.12 p<0.001 d=-0.08 | 0.15 ± 1.98 p<0.001 d=0.07 | 0.14 ± 1.65 p<0.001 d=0.09 | -0.05 ± 1.75 p=1.000 d=-0.03 |  |  |
| **24 months** | -1.19 ± 2.12 p<0.001 d=-0.56 | -0.25 ± 2.01 p<0.001 d=-0.13 | 0.07 ± 1.95 p=0.686 d=0.03 | 0.06 ± 1.85 p=0.712 d=0.03 | -0.12 ± 1.88 p=0.001 d=-0.07 | -0.08 ± 1.61 p=0.071 d=-0.05 |  |

**Supplementary Table 26. Pairwise Comparison of Short-Form McGill Pain Questionnaire-2 (SF-MPQ-2) Continuous Pain**

*Post-hoc pairwise comparisons between time points were performed using paired t-tests with Bonferroni-Holm correction for multiple comparisons. Effect sizes were calculated using Cohen's d for paired differences. SD – standard deviation*

| **Follow-up Interval** | **Mean Difference ± SD, p-value** | | | | | | |
| --- | --- | --- | --- | --- | --- | --- | --- |
|  | **Baseline** | **1 month** | **3 months** | **6 months** | **12 months** | **18 months** | **24 months** |
| **Baseline** |  |  |  |  |  |  |  |
| **1 month** | -1.00 ± 2.10 p<0.001 d=-0.48 |  |  |  |  |  |  |
| **3 months** | -1.38 ± 2.18 p<0.001 d=-0.63 | -0.38 ± 1.94 p<0.001 d=-0.20 |  |  |  |  |  |
| **6 months** | -1.43 ± 2.67 p<0.001 d=-0.53 | -0.42 ± 2.50 p<0.001 d=-0.17 | -0.04 ± 2.32 p=1.000 d=-0.02 |  |  |  |  |
| **12 months** | -1.24 ± 2.39 p<0.001 d=-0.52 | -0.24 ± 2.31 p<0.001 d=-0.10 | 0.14 ± 2.14 p<0.001 d=0.07 | 0.19 ± 2.17 p<0.001 d=0.09 |  |  |  |
| **18 months** | -2.14 ± 2.64 p<0.001 d=-0.81 | -1.14 ± 2.56 p<0.001 d=-0.44 | -0.76 ± 2.51 p<0.001 d=-0.30 | -0.71 ± 2.53 p<0.001 d=-0.28 | -0.90 ± 2.26 p<0.001 d=-0.40 |  |  |
| **24 months** | -0.66 ± 3.28 p<0.001 d=-0.20 | 0.35 ± 3.05 p<0.001 d=0.11 | 0.73 ± 3.04 p<0.001 d=0.24 | 0.77 ± 3.00 p<0.001 d=0.26 | 0.58 ± 2.67 p<0.001 d=0.22 | 1.48 ± 3.47 p<0.001 d=0.43 |  |

**Supplementary Table 27. Pairwise Comparison of Short-Form McGill Pain Questionnaire-2 (SF-MPQ-2) Intermittent Pain**

*Post-hoc pairwise comparisons between time points were performed using paired t-tests with Bonferroni-Holm correction for multiple comparisons. Effect sizes were calculated using Cohen's d for paired differences. SD – standard deviation*

| **Follow-up Interval** | **Mean Difference ± SD, p-value** | | | | | | |
| --- | --- | --- | --- | --- | --- | --- | --- |
|  | **Baseline** | **1 month** | **3 months** | **6 months** | **12 months** | **18 months** | **24 months** |
| **Baseline** |  |  |  |  |  |  |  |
| **1 month** | -0.91 ± 2.56 p<0.001 d=-0.36 |  |  |  |  |  |  |
| **3 months** | -1.26 ± 2.37 p<0.001 d=-0.53 | -0.35 ± 2.21 p<0.001 d=-0.16 |  |  |  |  |  |
| **6 months** | -1.26 ± 2.82 p<0.001 d=-0.44 | -0.34 ± 2.83 p<0.001 d=-0.12 | 0.01 ± 2.39 p=1.000 d=0.00 |  |  |  |  |
| **12 months** | -1.15 ± 2.64 p<0.001 d=-0.44 | -0.23 ± 2.65 p<0.001 d=-0.09 | 0.11 ± 2.33 p=0.058 d=0.05 | 0.11 ± 2.44 p=0.166 d=0.04 |  |  |  |
| **18 months** | -1.61 ± 2.69 p<0.001 d=-0.60 | -0.69 ± 2.60 p<0.001 d=-0.27 | -0.34 ± 2.52 p<0.001 d=-0.14 | -0.35 ± 2.39 p<0.001 d=-0.15 | -0.46 ± 2.06 p<0.001 d=-0.22 |  |  |
| **24 months** | -1.81 ± 2.70 p<0.001 d=-0.67 | -0.89 ± 2.61 p<0.001 d=-0.34 | -0.55 ± 2.45 p<0.001 d=-0.22 | -0.55 ± 2.43 p<0.001 d=-0.23 | -0.66 ± 2.41 p<0.001 d=-0.27 | -0.20 ± 2.27 p<0.001 d=-0.09 |  |

**Supplementary Table 28. Pairwise Comparison of Short-Form McGill Pain Questionnaire-2 (SF-MPQ-2) Neuropathic Pain**

*Post-hoc pairwise comparisons between time points were performed using paired t-tests with Bonferroni-Holm correction for multiple comparisons. Effect sizes were calculated using Cohen's d for paired differences. SD – standard deviation*

| **Follow-up Interval** | **Mean Difference ± SD, p-value** | | | | | | |
| --- | --- | --- | --- | --- | --- | --- | --- |
|  | **Baseline** | **1 month** | **3 months** | **6 months** | **12 months** | **18 months** | **24 months** |
| **Baseline** |  |  |  |  |  |  |  |
| **1 month** | -0.71 ± 1.91 p<0.001 d=-0.37 |  |  |  |  |  |  |
| **3 months** | -0.80 ± 1.91 p<0.001 d=-0.42 | -0.09 ± 1.69 p=0.022 d=-0.05 |  |  |  |  |  |
| **6 months** | -0.86 ± 2.17 p<0.001 d=-0.40 | -0.15 ± 1.96 p<0.001 d=-0.08 | -0.06 ± 1.81 p=1.000 d=-0.03 |  |  |  |  |
| **12 months** | -0.45 ± 2.59 p<0.001 d=-0.17 | 0.26 ± 2.36 p<0.001 d=0.11 | 0.35 ± 2.27 p<0.001 d=0.16 | 0.41 ± 2.39 p<0.001 d=0.17 |  |  |  |
| **18 months** | -1.22 ± 2.56 p<0.001 d=-0.48 | -0.51 ± 2.31 p<0.001 d=-0.22 | -0.41 ± 2.40 p<0.001 d=-0.17 | -0.36 ± 2.18 p<0.001 d=-0.16 | -0.77 ± 1.96 p<0.001 d=-0.39 |  |  |
| **24 months** | -1.77 ± 2.37 p<0.001 d=-0.75 | -1.06 ± 1.90 p<0.001 d=-0.55 | -0.97 ± 2.15 p<0.001 d=-0.45 | -0.91 ± 1.97 p<0.001 d=-0.46 | -1.32 ± 2.40 p<0.001 d=-0.55 | -0.55 ± 1.93 p<0.001 d=-0.29 |  |

**Supplementary Table 29. Pairwise Comparison of Pain Visual Analogue Scale (VAS)**

*Post-hoc pairwise comparisons between time points were performed using paired t-tests with Bonferroni-Holm correction for multiple comparisons. Effect sizes were calculated using Cohen's d for paired differences. SD – standard deviation*

| **Follow-up Interval** | **Mean Difference ± SD, p-value** | | | | | | |
| --- | --- | --- | --- | --- | --- | --- | --- |
|  | **Baseline** | **1 month** | **3 months** | **6 months** | **12 months** | **18 months** | **24 months** |
| **Baseline** |  |  |  |  |  |  |  |
| **1 month** | -1.14 ± 2.28 p<0.001 d=-0.50 |  |  |  |  |  |  |
| **3 months** | -1.37 ± 2.36 p<0.001 d=-0.58 | -0.23 ± 2.21 p<0.001 d=-0.11 |  |  |  |  |  |
| **6 months** | -1.51 ± 2.50 p<0.001 d=-0.60 | -0.37 ± 2.45 p<0.001 d=-0.15 | -0.14 ± 2.30 p=0.006 d=-0.06 |  |  |  |  |
| **12 months** | -1.40 ± 2.54 p<0.001 d=-0.55 | -0.26 ± 2.56 p<0.001 d=-0.10 | -0.03 ± 2.35 p=1.000 d=-0.01 | 0.11 ± 2.30 p=0.070 d=0.05 |  |  |  |
| **18 months** | -1.49 ± 2.61 p<0.001 d=-0.57 | -0.35 ± 2.70 p<0.001 d=-0.13 | -0.11 ± 2.60 p=0.158 d=-0.04 | 0.02 ± 2.55 p=1.000 d=0.01 | -0.09 ± 2.30 p=0.422 d=-0.04 |  |  |
| **24 months** | -1.22 ± 2.67 p<0.001 d=-0.46 | -0.08 ± 2.81 p=1.000 d=-0.03 | 0.15 ± 2.70 p=0.014 d=0.06 | 0.29 ± 2.69 p<0.001 d=0.11 | 0.18 ± 2.51 p<0.001 d=0.07 | 0.26 ± 2.50 p<0.001 d=0.11 |  |

**Supplementary Table 30. Proportion of Patients Reporting an Improvement or Minimal Clinically Important Difference (MCID) in Patient-Reported Outcome Measures (PROMs)**

The proportion of individuals reporting a MCID from baseline was assessed for Generalised Anxiety Disorder-7 (GAD-7) and the Single-Item Sleep Quality Scale (SQS). For EuroQol 5-Dimension 5-Level (EQ-5D-5L) index values the proportion of individuals reporting any positive improvement from baseline was calculated considering no universally accepted MCID value exists for this PROM.

| **Patient-Reported Outcome Measures** | **n (%)** | | | | | |
| --- | --- | --- | --- | --- | --- | --- |
|  | **1 month** | **3 months** | **6 months** | **12 months** | **18 months** | **24 months** |
| **EQ-5D-5L Index Value Improvement** | 2,790 (74.52%) | 2,833 (75.67%) | 2,842 (75.91%) | 2,846 (76.01%) | 2,857 (76.31%) | 2,872 (76.71%) |
| **MCID GAD-7** | 1,437 (38.38%) | 1,503 (40.14%) | 1,521 (40.63%) | 1,566 (41.83%) | 1,561 (41.69%) | 1,587 (42.39%) |
| **MCID SQS** | 1,407 (37.58%) | 1,486 (39.69%) | 1,467 (39.18%) | 1,444 (38.57%) | 1,551 (41.43%) | 1,464 (39.10%) |

**Supplementary Table 31. Proportion of Patients Reporting an Improvement or Minimal Clinically Important Difference (MCID), Moderately Important Improvement (MII) and Substantially Important Improvement (SII) in Pain-Specific Patient-Reported Outcome Measures (PROMs)**

The proportion of individuals reporting a MCID, MII, and SII from baseline was assessed for Brief Pain Inventory – Short Form (BPI-SF), Short-Form McGill Pain Questionnaire-2 (SF-MPQ-2), and Pain Visual Analogue Scale (VAS).

| **Patient-Reported Outcome Measures** | **n (%)** | | | | | |
| --- | --- | --- | --- | --- | --- | --- |
|  | **1 month** | **3 months** | **6 months** | **12 months** | **18 months** | **24 months** |
| **MCID BPI-SF Severity** | 1,777 (47.46%) | 2,037 (54.41%) | 2,046 (54.65%) | 2,184 (58.33%) | 1,979 (52.86%) | 2,045 (54.62%) |
| **MCID BPI-SF Interference** | 2,008 (53.63%) | 2,248 (60.04%) | 2,240 (59.83%) | 2,425 (64.77%) | 2,199 (58.73%) | 2,350 (62.77%) |
| **MCID SF-MPQ-2 Total Score** | 1,686 (45.03%) | 2,015 (53.82%) | 1,957 (52.27%) | 1,977 (52.80%) | 1,739 (46.45%) | 2,075 (55.42%) |
| **MCID Pain VAS** | 2,202 (58.81%) | 2,311 (61.73%) | 2,349 (62.74%) | 2,418 (64.58%) | 2,287 (61.08%) | 2,289 (61.14%) |
| **MII BPI-SF Severity** | 990 (26.44%) | 1,225 (32.72%) | 1,291 (34.48%) | 1,390 (37.13%) | 1,215 (32.45%) | 1,285 (34.32%) |
| **MII BPI-SF Interference** | 1,223 (32.67%) | 1,492 (39.85%) | 1,492 (39.85%) | 1,660 (44.34%) | 1,442 (38.51%) | 1,575 (42.07%) |
| **MII SF-MPQ-2 Total Score** | 1,347 (35.98%) | 1,765 (47.14%) | 1,655 (44.20%) | 1,728 (46.15%) | 1,451 (38.76%) | 1,774 (47.38%) |
| **MII Pain VAS** | 1,057 (28.23%) | 1,243 (33.20%) | 1,394 (37.23%) | 1,451 (38.76%) | 1,317 (35.18%) | 1,303 (34.80%) |
| **SII BPI-SF Severity** | 387 (10.34%) | 525 (14.02%) | 571 (15.25%) | 599 (16.00%) | 495 (13.22%) | 557 (14.88%) |
| **SII BPI-SF Interference** | 574 (15.33%) | 743 (19.85%) | 780 (20.83%) | 865 (23.10%) | 721 (19.26%) | 794 (21.21%) |
| **SII SF-MPQ-2 Total Score** | 696 (18.59%) | 1,076 (28.74%) | 965 (25.77%) | 1,027 (27.43%) | 800 (21.37%) | 930 (24.84%) |
| **SII Pain VAS** | 615 (16.43%) | 759 (20.27%) | 869 (23.21%) | 928 (24.79%) | 794 (21.21%) | 751 (20.06%) |

**Supplementary Table 32. Univariable Logistic Regression for Positive Improvement in EQ-5D-5L Index Value at 24 Months.**

*BMI – body mass index; CBD – cannabidiol; CBMPs – cannabis-based medicinal products; CI – confidence interval; EQ-5D-5L – EuroQol 5-Dimension 5-Level; GAD-7 – Generalised Anxiety Disorder-7; Max – maximum; Min – minimum; OR – odds ratio; Q1 – quartile 1; Q3 – quartile 3; SQS – Single-Item Sleep Quality Scale; THC – (-)-trans-Δ9-tetrahydrocannabinol.*

| **Variable** | **n** | **OR (95% CI)** | **p-value** |
| --- | --- | --- | --- |
| **Primary Diagnosis** |  |  |  |
| Chronic Non-Cancer Pain | 2,276 | Ref. | - |
| Cancer pain | 116 | 0.59 (0.40-0.89) | 0.009 |
| Complex regional pain syndrome | 44 | 1.58 (0.75-3.89) | 0.270 |
| Endometriosis | 101 | 0.74 (0.48-1.17) | 0.187 |
| Hypermobility | 240 | 1.81 (1.26-2.68) | 0.002 |
| Inflammatory arthritis | 192 | 1.21 (0.85-1.77) | 0.306 |
| Neuropathic pain | 600 | 0.83 (0.68-1.02) | 0.075 |
| Osteoarthritis | 175 | 0.95 (0.67-1.37) | 0.768 |
| **Age (Years)** |  |  |  |
| 18-30 | 416 | Ref. | - |
| 31-40 | 1,007 | 0.70 (0.52-0.93) | 0.015 |
| 41-50 | 1,027 | 0.65 (0.48-0.86) | 0.003 |
| 51-60 | 728 | 0.81 (0.60-1.10) | 0.186 |
| 61+ | 566 | 0.67 (0.49-0.91) | 0.012 |
| **Sex** |  |  |  |
| Male | 2,172 | Ref. | - |
| Female | 1,572 | 1.28 (1.09-1.49) | 0.002 |
| **BMI (kg/m2)** |  |  |  |
| 20-24.99 | 1,093 | Ref. | - |
| <20 | 296 | 1.33 (0.98-1.82) | 0.071 |
| 25-29.99 | 1,165 | 1.13 (0.93-1.36) | 0.222 |
| 30-34.99 | 551 | 1.54 (1.20-1.99) | <0.001 |
| 35+ | 455 | 1.46 (1.12-1.91) | 0.006 |
| **Cannabis Status** |  |  |  |
| Never Consumed Cannabis | 1,002 | Ref. | - |
| Former Cannabis Consumer | 590 | 1.14 (0.90-1.45) | 0.287 |
| Current Cannabis Consumer | 2,152 | 1.09 (0.91-1.30) | 0.339 |

| **CBMPs at 24 Months** |  |  |  |
| --- | --- | --- | --- |
| Oil | 879 | Ref. | - |
| Dried flower/flos | 801 | 1.36 (1.09-1.71) | 0.008 |
| Dried flower/flos, Oil | 1,748 | 1.21 (1.00-1.46) | 0.047 |
| Dried flower/flos, Pastille | 77 | 1.02 (0.61-1.78) | 0.936 |
| Dried flower/flos, Oil, Pastille | 68 | 1.27 (0.72-2.37) | 0.434 |
| Other | 89 | 1.77 (1.02-3.26) | 0.052 |
| None | 82 | 0.92 (0.56-1.55) | 0.745 |
| **Daily CBD Dose (mg/day) at 24 Months** |  |  |  |
| Min to Q1 | 962 | Ref. | - |
| Q1 to Median | 914 | 1.04 (0.84-1.29) | 0.735 |
| Median to Q3 | 932 | 0.88 (0.71-1.09) | 0.232 |
| Q3 to Max | 936 | 1.02 (0.83-1.27) | 0.823 |
| **Daily THC Dose (mg/day) at 24 Months** |  |  |  |
| Min to Q1 | 936 | Ref. | - |
| Q1 to Median | 942 | 1.22 (0.99-1.51) | 0.058 |
| Median to Q3 | 949 | 1.36 (1.10-1.69) | 0.004 |
| Q3 to Max | 917 | 1.29 (1.04-1.60) | 0.019 |
| **SQS at Baseline** |  |  |  |
| 7-10 | 558 | Ref. | - |
| 4-6 | 1,212 | 1.62 (1.31-2.01) | <0.001 |
| 0-3 | 1,974 | 3.43 (2.78-4.22) | <0.001 |
| **GAD-7 at Baseline** |  |  |  |
| <10 | 2,245 | Ref. | - |
| 10-14 | 683 | 2.75 (2.18-3.50) | <0.001 |
| 15+ | 816 | 3.41 (2.71-4.34) | <0.001 |
| **Prescribed Opioids at Baseline** |  |  |  |
| No | 2,300 | Ref. | - |
| Yes | 1,444 | 1.47 (1.25-1.73) | <0.001 |
| **Prescribed Gabapentinoids at Baseline** |  |  |  |
| No | 3,020 | Ref. | - |
| Yes | 724 | 1.57 (1.28-1.95) | <0.001 |

**Supplementary Table 33. Multivariable Logistic Regression for Positive Improvement in EQ-5D-5L Index Value at 24 Months.**

*BMI – body mass index; CBD – cannabidiol; CBMPs – cannabis-based medicinal products; CI – confidence interval; EQ-5D-5L – EuroQol 5-Dimension 5-Level; GAD-7 – Generalised Anxiety Disorder-7; Max – maximum; Min – minimum; OR – odds ratio; Q1 – quartile 1; Q3 – quartile 3; SQS – Single-Item Sleep Quality Scale; THC – (-)-trans-Δ9-tetrahydrocannabinol.*

| **Variable** | **n** | **OR (95% CI)** | **p-value** |
| --- | --- | --- | --- |
| **Primary Diagnosis** |  |  |  |
| Chronic Non-Cancer Pain | 2,153 | Ref. | - |
| Cancer pain | 110 | 0.66 (0.43-1.04) | 0.072 |
| Complex regional pain syndrome | 42 | 1.37 (0.62-3.46) | 0.471 |
| Endometriosis | 97 | 0.80 (0.49-1.33) | 0.375 |
| Hypermobility | 228 | 1.65 (1.11-2.52) | 0.017 |
| Inflammatory arthritis | 187 | 1.33 (0.91-1.98) | 0.148 |
| Neuropathic pain | 576 | 0.90 (0.72-1.13) | 0.373 |
| Osteoarthritis | 167 | 0.97 (0.66-1.45) | 0.892 |
| **Age (Years)** |  |  |  |
| 18-30 | 393 | Ref. | - |
| 31-40 | 969 | 0.67 (0.48-0.91) | 0.012 |
| 41-50 | 990 | 0.60 (0.43-0.82) | 0.002 |
| 51-60 | 697 | 0.74 (0.52-1.04) | 0.083 |
| 61+ | 511 | 0.79 (0.54-1.14) | 0.208 |
| **Sex** |  |  |  |
| Male | 2,077 | Ref. | - |
| Female | 1,483 | 1.13 (0.93-1.37) | 0.217 |
| **BMI (kg/m2)** |  |  |  |
| 20-24.99 | 1,093 | Ref. | - |
| <20 | 296 | 1.24 (0.90-1.73) | 0.190 |
| 25-29.99 | 1,165 | 1.16 (0.95-1.41) | 0.155 |
| 30-34.99 | 551 | 1.46 (1.12-1.90) | 0.005 |
| 35+ | 455 | 1.17 (0.88-1.56) | 0.280 |
| **Cannabis Status** |  |  |  |
| Never Consumed Cannabis | 923 | Ref. | - |
| Former Cannabis Consumer | 571 | 1.10 (0.83-1.46) | 0.499 |
| Current Cannabis Consumer | 2,066 | 1.04 (0.81-1.32) | 0.775 |

| **CBMPs at 24 Months** |  |  |  |
| --- | --- | --- | --- |
| Oil | 821 | Ref. | - |
| Dried flower/flos | 777 | 0.96 (0.51-1.77) | 0.905 |
| Dried flower/flos, Oil | 1,667 | 0.82 (0.45-1.46) | 0.505 |
| Dried flower/flos, Pastille | 76 | 0.52 (0.23-1.17) | 0.113 |
| Dried flower/flos, Oil, Pastille | 68 | 0.93 (0.40-2.18) | 0.862 |
| Other | 84 | 1.55 (0.78-3.22) | 0.227 |
| None | 67 | 0.81 (0.44-1.51) | 0.490 |
| **Daily CBD Dose (mg/day) at 24 Months** |  |  |  |
| Min to Q1 | 898 | Ref. | - |
| Q1 to Median | 863 | 1.00 (0.77-1.31) | 0.973 |
| Median to Q3 | 888 | 0.87 (0.67-1.13) | 0.294 |
| Q3 to Max | 911 | 0.91 (0.70-1.17) | 0.449 |
| **Daily THC Dose (mg/day) at 24 Months** |  |  |  |
| Min to Q1 | 863 | Ref. | - |
| Q1 to Median | 879 | 1.48 (0.84-2.69) | 0.183 |
| Median to Q3 | 916 | 1.61 (0.88-3.02) | 0.130 |
| Q3 to Max | 902 | 1.62 (0.87-3.06) | 0.131 |
| **SQS at Baseline** |  |  |  |
| 7-10 | 533 | Ref. | - |
| 4-6 | 1,155 | 1.45 (1.16-1.81) | 0.001 |
| 0-3 | 1,872 | 2.52 (2.00-3.16) | <0.001 |
| **GAD-7 at Baseline** |  |  |  |
| <10 | 2,139 | Ref. | - |
| 10-14 | 649 | 2.38 (1.85-3.08) | <0.001 |
| 15+ | 772 | 2.46 (1.93-3.18) | <0.001 |
| **Prescribed Opioids at Baseline** |  |  |  |
| No | 2,177 | Ref. | - |
| Yes | 1,383 | 1.25 (1.05-1.49) | 0.014 |
| **Prescribed Gabapentinoids at Baseline** |  |  |  |
| No | 2,876 | Ref. | - |
| Yes | 684 | 1.33 (1.06-1.68) | 0.015 |

**Supplementary Table 34. Univariable Logistic Regression for a MCID in GAD-7 at 24 Months.**

*BMI – body mass index; CBD – cannabidiol; CBMPs – cannabis-based medicinal products; CI – confidence interval; GAD-7 – Generalised Anxiety Disorder-7; Max – maximum; MCID – minimal clinically important difference; Min – minimum; OR – odds ratio; Q1 – quartile 1; Q3 – quartile 3; SQS – Single-Item Sleep Quality Scale; THC – (-)-trans-Δ9-tetrahydrocannabinol.*

| **Variable** | **n** | **OR (95% CI)** | **p-value** |
| --- | --- | --- | --- |
| **Primary Diagnosis** |  |  |  |
| Chronic Non-Cancer Pain | 2,276 | Ref. | - |
| Cancer pain | 116 | 1.04 (0.71-1.51) | 0.850 |
| Complex regional pain syndrome | 44 | 1.06 (0.58-1.93) | 0.841 |
| Endometriosis | 101 | 0.87 (0.58-1.30) | 0.508 |
| Hypermobility | 240 | 1.08 (0.83-1.41) | 0.574 |
| Inflammatory arthritis | 192 | 0.67 (0.49-0.91) | 0.011 |
| Neuropathic pain | 600 | 0.81 (0.67-0.97) | 0.025 |
| Osteoarthritis | 175 | 0.75 (0.55-1.03) | 0.081 |
| **Age (Years)** |  |  |  |
| 18-30 | 416 | Ref. | - |
| 31-40 | 1,007 | 0.83 (0.66-1.04) | 0.101 |
| 41-50 | 1,027 | 0.73 (0.58-0.92) | 0.007 |
| 51-60 | 728 | 0.70 (0.55-0.89) | 0.003 |
| 61+ | 566 | 0.55 (0.42-0.71) | <0.001 |
| **Sex** |  |  |  |
| Male | 2,172 | Ref. | - |
| Female | 1,572 | 1.10 (0.96-1.25) | 0.166 |
| **BMI (kg/m2)** |  |  |  |
| 20-24.99 | 1,093 | Ref. | - |
| <20 | 296 | 0.88 (0.68-1.14) | 0.337 |
| 25-29.99 | 1,165 | 0.92 (0.78-1.09) | 0.348 |
| 30-34.99 | 551 | 0.96 (0.78-1.18) | 0.677 |
| 35+ | 455 | 1.21 (0.97-1.50) | 0.096 |
| **Cannabis Status** |  |  |  |
| Never Consumed Cannabis | 1,002 | Ref. | - |
| Former Cannabis Consumer | 590 | 1.32 (1.07-1.62) | 0.010 |
| Current Cannabis Consumer | 2,152 | 1.44 (1.23-1.68) | <0.001 |

| **CBMPs at 24 Months** |  |  |  |
| --- | --- | --- | --- |
| Oil | 879 | Ref. | - |
| Dried flower/flos | 801 | 1.30 (1.07-1.58) | 0.008 |
| Dried flower/flos, Oil | 1,748 | 1.43 (1.21-1.69) | <0.001 |
| Dried flower/flos, Pastille | 77 | 1.52 (0.95-2.43) | 0.080 |
| Dried flower/flos, Oil, Pastille | 68 | 1.07 (0.64-1.77) | 0.792 |
| Other | 89 | 1.17 (0.75-1.83) | 0.478 |
| None | 82 | 1.05 (0.65-1.67) | 0.833 |
| **Daily CBD Dose (mg/day) at 24 Months** |  |  |  |
| Min to Q1 | 962 | Ref. | - |
| Q1 to Median | 914 | 1.05 (0.87-1.26) | 0.614 |
| Median to Q3 | 932 | 0.94 (0.78-1.13) | 0.503 |
| Q3 to Max | 936 | 0.95 (0.79-1.13) | 0.544 |
| **Daily THC Dose (mg/day) at 24 Months** |  |  |  |
| Min to Q1 | 936 | Ref. | - |
| Q1 to Median | 942 | 1.53 (1.27-1.84) | <0.001 |
| Median to Q3 | 949 | 1.46 (1.22-1.76) | <0.001 |
| Q3 to Max | 917 | 1.45 (1.20-1.74) | <0.001 |
| **SQS at Baseline** |  |  |  |
| 7-10 | 558 | Ref. | - |
| 4-6 | 1,212 | 2.16 (1.72-2.75) | <0.001 |
| 0-3 | 1,974 | 4.10 (3.29-5.13) | <0.001 |
| **GAD-7 at Baseline** |  |  |  |
| <10 | 2,245 | Ref. | - |
| 10-14 | 683 | 11.76 (9.66-14.37) | <0.001 |
| 15+ | 816 | 26.41 (21.25-33.08) | <0.001 |
| **Prescribed Opioids at Baseline** |  |  |  |
| No | 2,300 | Ref. | - |
| Yes | 1,444 | 1.15 (1.01-1.32) | 0.036 |
| **Prescribed Gabapentinoids at Baseline** |  |  |  |
| No | 3,020 | Ref. | - |
| Yes | 724 | 1.36 (1.15-1.60) | <0.001 |

**Supplementary Table 35. Multivariable Logistic Regression for a MCID in GAD-7 at 24 Months.**

*BMI – body mass index; CBD – cannabidiol; CBMPs – cannabis-based medicinal products; CI – confidence interval; GAD-7 – Generalised Anxiety Disorder-7; Max – maximum; MCID – minimal clinically important difference; Min – minimum; OR – odds ratio; Q1 – quartile 1; Q3 – quartile 3; SQS – Single-Item Sleep Quality Scale; THC – (-)-trans-Δ9-tetrahydrocannabinol.*

| **Variable** | **n** | **OR (95% CI)** | **p-value** |
| --- | --- | --- | --- |
| **Primary Diagnosis** |  |  |  |
| Chronic Non-Cancer Pain | 2,153 | Ref. | - |
| Cancer pain | 110 | 1.27 (0.76-2.12) | 0.360 |
| Complex regional pain syndrome | 42 | 0.72 (0.32-1.59) | 0.419 |
| Endometriosis | 97 | 1.13 (0.65-1.93) | 0.665 |
| Hypermobility | 228 | 1.04 (0.72-1.49) | 0.839 |
| Inflammatory arthritis | 187 | 0.81 (0.55-1.20) | 0.309 |
| Neuropathic pain | 576 | 0.90 (0.70-1.14) | 0.382 |
| Osteoarthritis | 167 | 1.05 (0.69-1.59) | 0.825 |
| **Age (Years)** |  |  |  |
| 18-30 | 393 | Ref. | - |
| 31-40 | 969 | 0.97 (0.71-1.31) | 0.825 |
| 41-50 | 990 | 0.84 (0.62-1.14) | 0.260 |
| 51-60 | 697 | 0.80 (0.58-1.11) | 0.185 |
| 61+ | 511 | 0.89 (0.62-1.29) | 0.540 |
| **Sex** |  |  |  |
| Male | 2,077 | Ref. | - |
| Female | 1,483 | 1.02 (0.84-1.24) | 0.827 |
| **BMI (kg/m2)** |  |  |  |
| 20-24.99 | 1,093 | Ref. | - |
| <20 | 296 | 0.65 (0.46-0.91) | 0.013 |
| 25-29.99 | 1,165 | 0.92 (0.74-1.13) | 0.421 |
| 30-34.99 | 551 | 0.86 (0.65-1.12) | 0.255 |
| 35+ | 455 | 0.95 (0.71-1.27) | 0.733 |
| **Cannabis Status** |  |  |  |
| Never Consumed Cannabis | 923 | Ref. | - |
| Former Cannabis Consumer | 571 | 1.05 (0.78-1.40) | 0.758 |
| Current Cannabis Consumer | 2,066 | 1.03 (0.80-1.33) | 0.809 |

| **CBMPs at 24 Months** |  |  |  |
| --- | --- | --- | --- |
| Oil | 821 | Ref. | - |
| Dried flower/flos | 777 | 0.45 (0.25-0.83) | 0.010 |
| Dried flower/flos, Oil | 1,667 | 0.47 (0.26-0.82) | 0.008 |
| Dried flower/flos, Pastille | 76 | 0.32 (0.14-0.73) | 0.007 |
| Dried flower/flos, Oil, Pastille | 68 | 0.32 (0.14-0.74) | 0.009 |
| Other | 84 | 0.55 (0.27-1.09) | 0.090 |
| None | 67 | 1.72 (0.86-3.36) | 0.117 |
| **Daily CBD Dose (mg/day) at 24 Months** |  |  |  |
| Min to Q1 | 898 | Ref. | - |
| Q1 to Median | 863 | 1.18 (0.90-1.54) | 0.231 |
| Median to Q3 | 888 | 0.98 (0.75-1.28) | 0.880 |
| Q3 to Max | 911 | 0.93 (0.71-1.21) | 0.578 |
| **Daily THC Dose (mg/day) at 24 Months** |  |  |  |
| Min to Q1 | 863 | Ref. | - |
| Q1 to Median | 879 | 3.62 (2.06-6.37) | <0.001 |
| Median to Q3 | 916 | 3.64 (1.99-6.67) | <0.001 |
| Q3 to Max | 902 | 3.40 (1.84-6.30) | <0.001 |
| **SQS at Baseline** |  |  |  |
| 7-10 | 533 | Ref. | - |
| 4-6 | 1,155 | 1.71 (1.29-2.28) | <0.001 |
| 0-3 | 1,872 | 1.76 (1.34-2.33) | <0.001 |
| **GAD-7 at Baseline** |  |  |  |
| <10 | 2,139 | Ref. | - |
| 10-14 | 649 | 11.23 (9.10-13.90) | <0.001 |
| 15+ | 772 | 24.36 (19.29-31.01) | <0.001 |
| **Prescribed Opioids at Baseline** |  |  |  |
| No | 2,177 | Ref. | - |
| Yes | 1,383 | 1.03 (0.86-1.24) | 0.722 |
| **Prescribed Gabapentinoids at Baseline** |  |  |  |
| No | 2,876 | Ref. | - |
| Yes | 684 | 1.02 (0.82-1.28) | 0.846 |

**Supplementary Table 36. Univariable Logistic Regression for a MCID in Single-Item Sleep Quality Scale at 24 Months.**

*BMI – body mass index; CBD – cannabidiol; CBMPs – cannabis-based medicinal products; CI – confidence interval; GAD-7 – Generalised Anxiety Disorder-7; Max – maximum; MCID – minimal clinically important difference; Min – minimum; OR – odds ratio; Q1 – quartile 1; Q3 – quartile 3; SQS – Single-Item Sleep Quality Scale; THC – (-)-trans-Δ9-tetrahydrocannabinol.*

| **Variable** | **n** | **OR (95% CI)** | **p-value** |
| --- | --- | --- | --- |
| **Primary Diagnosis** |  |  |  |
| Chronic Non-Cancer Pain | 2,276 | Ref. | - |
| Cancer pain | 116 | 0.85 (0.57-1.25) | 0.424 |
| Complex regional pain syndrome | 44 | 0.56 (0.28-1.07) | 0.093 |
| Endometriosis | 101 | 0.67 (0.43-1.02) | 0.065 |
| Hypermobility | 240 | 1.07 (0.82-1.41) | 0.603 |
| Inflammatory arthritis | 192 | 0.82 (0.60-1.12) | 0.219 |
| Neuropathic pain | 600 | 0.99 (0.82-1.19) | 0.904 |
| Osteoarthritis | 175 | 0.87 (0.63-1.19) | 0.381 |
| **Age (Years)** |  |  |  |
| 18-30 | 416 | Ref. | - |
| 31-40 | 1,007 | 1.06 (0.84-1.34) | 0.630 |
| 41-50 | 1,027 | 1.01 (0.80-1.27) | 0.944 |
| 51-60 | 728 | 1.04 (0.82-1.34) | 0.732 |
| 61+ | 566 | 0.88 (0.68-1.14) | 0.324 |
| **Sex** |  |  |  |
| Male | 2,172 | Ref. | - |
| Female | 1,572 | 1.07 (0.94-1.22) | 0.332 |
| **BMI (kg/m2)** |  |  |  |
| 20-24.99 | 1,093 | Ref. | - |
| <20 | 296 | 0.95 (0.73-1.24) | 0.728 |
| 25-29.99 | 1,165 | 1.01 (0.85-1.20) | 0.893 |
| 30-34.99 | 551 | 1.01 (0.82-1.25) | 0.928 |
| 35+ | 455 | 1.15 (0.92-1.44) | 0.217 |
| **Cannabis Status** |  |  |  |
| Never Consumed Cannabis | 1,002 | Ref. | - |
| Former Cannabis Consumer | 590 | 0.99 (0.81-1.22) | 0.944 |
| Current Cannabis Consumer | 2,152 | 0.90 (0.78-1.05) | 0.195 |

| **CBMPs at 24 Months** |  |  |  |
| --- | --- | --- | --- |
| Oil | 879 | Ref. | - |
| Dried flower/flos | 801 | 0.89 (0.73-1.08) | 0.228 |
| Dried flower/flos, Oil | 1,748 | 1.08 (0.92-1.28) | 0.339 |
| Dried flower/flos, Pastille | 77 | 0.86 (0.52-1.38) | 0.532 |
| Dried flower/flos, Oil, Pastille | 68 | 1.33 (0.80-2.18) | 0.262 |
| Other | 89 | 1.29 (0.83-2.01) | 0.250 |
| None | 82 | 0.87 (0.53-1.38) | 0.555 |
| **Daily CBD Dose (mg/day) at 24 Months** |  |  |  |
| Min to Q1 | 962 | Ref. | - |
| Q1 to Median | 914 | 1.02 (0.85-1.23) | 0.837 |
| Median to Q3 | 932 | 1.09 (0.91-1.31) | 0.354 |
| Q3 to Max | 936 | 1.09 (0.91-1.32) | 0.343 |
| **Daily THC Dose (mg/day) at 24 Months** |  |  |  |
| Min to Q1 | 936 | Ref. | - |
| Q1 to Median | 942 | 1.00 (0.83-1.21) | 0.974 |
| Median to Q3 | 949 | 1.07 (0.89-1.29) | 0.482 |
| Q3 to Max | 917 | 1.04 (0.86-1.25) | 0.689 |
| **SQS at Baseline** |  |  |  |
| 7-10 | 558 | Ref. | - |
| 4-6 | 1,212 | 15.71 (9.47-28.39) | <0.001 |
| 0-3 | 1,974 | 49.01 (29.77-88.02) | <0.001 |
| **GAD-7 at Baseline** |  |  |  |
| <10 | 2,245 | Ref. | - |
| 10-14 | 683 | 1.48 (1.24-1.76) | <0.001 |
| 15+ | 816 | 1.70 (1.45-2.01) | <0.001 |
| **Prescribed Opioids at Baseline** |  |  |  |
| No | 2,300 | Ref. | - |
| Yes | 1,444 | 1.22 (1.07-1.40) | 0.004 |
| **Prescribed Gabapentinoids at Baseline** |  |  |  |
| No | 3,020 | Ref. | - |
| Yes | 724 | 1.10 (0.93-1.29) | 0.274 |

**Supplementary Table 37. Multivariable Logistic Regression for a MCID in Single-Item Sleep Quality Scale at 24 Months.**

*BMI – body mass index; CBD – cannabidiol; CBMPs – cannabis-based medicinal products; CI – confidence interval; GAD-7 – Generalised Anxiety Disorder-7; Max – maximum; MCID – minimal clinically important difference; Min – minimum; OR – odds ratio; Q1 – quartile 1; Q3 – quartile 3; SQS – Single-Item Sleep Quality Scale; THC – (-)-trans-Δ9-tetrahydrocannabinol.*

| **Variable** | **n** | **OR (95% CI)** | **p-value** |
| --- | --- | --- | --- |
| **Primary Diagnosis** |  |  |  |
| Chronic Non-Cancer Pain | 2,153 | Ref. | - |
| Cancer pain | 110 | 1.07 (0.68-1.68) | 0.771 |
| Complex regional pain syndrome | 42 | 0.51 (0.24-1.02) | 0.065 |
| Endometriosis | 97 | 0.83 (0.49-1.37) | 0.462 |
| Hypermobility | 228 | 0.93 (0.68-1.28) | 0.677 |
| Inflammatory arthritis | 187 | 0.79 (0.56-1.11) | 0.182 |
| Neuropathic pain | 576 | 1.09 (0.88-1.34) | 0.447 |
| Osteoarthritis | 167 | 0.95 (0.66-1.37) | 0.793 |
| **Age (Years)** |  |  |  |
| 18-30 | 393 | Ref. | - |
| 31-40 | 969 | 0.97 (0.75-1.28) | 0.851 |
| 41-50 | 990 | 0.85 (0.65-1.12) | 0.248 |
| 51-60 | 697 | 0.83 (0.62-1.10) | 0.192 |
| 61+ | 511 | 0.78 (0.56-1.08) | 0.132 |
| **Sex** |  |  |  |
| Male | 2,077 | Ref. | - |
| Female | 1,483 | 1.01 (0.85-1.20) | 0.896 |
| **BMI (kg/m2)** |  |  |  |
| 20-24.99 | 1,093 | Ref. | - |
| <20 | 296 | 0.92 (0.68-1.23) | 0.580 |
| 25-29.99 | 1,165 | 1.00 (0.82-1.20) | 0.965 |
| 30-34.99 | 551 | 0.88 (0.70-1.11) | 0.273 |
| 35+ | 455 | 0.90 (0.70-1.15) | 0.393 |
| **Cannabis Status** |  |  |  |
| Never Consumed Cannabis | 923 | Ref. | - |
| Former Cannabis Consumer | 571 | 0.88 (0.68-1.13) | 0.304 |
| Current Cannabis Consumer | 2,066 | 0.85 (0.68-1.06) | 0.146 |

| **CBMPs at 24 Months** |  |  |  |
| --- | --- | --- | --- |
| Oil | 821 | Ref. | - |
| Dried flower/flos | 777 | 0.77 (0.46-1.30) | 0.332 |
| Dried flower/flos, Oil | 1,667 | 0.90 (0.55-1.49) | 0.686 |
| Dried flower/flos, Pastille | 76 | 0.58 (0.28-1.17) | 0.131 |
| Dried flower/flos, Oil, Pastille | 68 | 1.26 (0.60-2.66) | 0.547 |
| Other | 84 | 1.20 (0.66-2.19) | 0.540 |
| None | 67 | 0.76 (0.42-1.37) | 0.370 |
| **Daily CBD Dose (mg/day) at 24 Months** |  |  |  |
| Min to Q1 | 898 | Ref. | - |
| Q1 to Median | 863 | 0.90 (0.71-1.13) | 0.355 |
| Median to Q3 | 888 | 1.00 (0.79-1.27) | 0.969 |
| Q3 to Max | 911 | 0.99 (0.78-1.24) | 0.900 |
| **Daily THC Dose (mg/day) at 24 Months** |  |  |  |
| Min to Q1 | 863 | Ref. | - |
| Q1 to Median | 879 | 1.04 (0.63-1.72) | 0.868 |
| Median to Q3 | 916 | 1.23 (0.72-2.10) | 0.439 |
| Q3 to Max | 902 | 1.23 (0.71-2.11) | 0.459 |
| **SQS at Baseline** |  |  |  |
| 7-10 | 533 | Ref. | - |
| 4-6 | 1,155 | 16.32 (9.65-30.23) | <0.001 |
| 0-3 | 1,872 | 53.17 (31.58-98.23) | <0.001 |
| **GAD-7 at Baseline** |  |  |  |
| <10 | 2,139 | Ref. | - |
| 10-14 | 649 | 1.00 (0.82-1.22) | 0.972 |
| 15+ | 772 | 0.92 (0.76-1.11) | 0.388 |
| **Prescribed Opioids at Baseline** |  |  |  |
| No | 2,177 | Ref. | - |
| Yes | 1,383 | 1.08 (0.92-1.26) | 0.354 |
| **Prescribed Gabapentinoids at Baseline** |  |  |  |
| No | 2,876 | Ref. | - |
| Yes | 684 | 0.98 (0.80-1.18) | 0.806 |

**Supplementary Table 38. Univariable Logistic Regression for a MCID in BPI-SF Pain Severity at 24 Months.**

*BMI – body mass index; BPI-SF – Brief Pain Inventory Short Form; CBD – cannabidiol; CBMPs – cannabis-based medicinal products; CI – confidence interval; GAD-7 – Generalised Anxiety Disorder-7; Max – maximum; MCID – minimal clinically important difference; Min – minimum; OR – odds ratio; Q1 – quartile 1; Q3 – quartile 3; SQS – Single-Item Sleep Quality Scale; THC – (-)-trans-Δ9-tetrahydrocannabinol.*

| **Variable** | **n** | **OR (95% CI)** | **p-value** |
| --- | --- | --- | --- |
| **Primary Diagnosis** |  |  |  |
| Chronic Non-Cancer Pain | 2,276 | Ref. | - |
| Cancer pain | 116 | 0.73 (0.50-1.06) | 0.100 |
| Complex regional pain syndrome | 44 | 1.74 (0.93-3.39) | 0.091 |
| Endometriosis | 101 | 0.86 (0.58-1.28) | 0.460 |
| Hypermobility | 240 | 0.82 (0.63-1.08) | 0.155 |
| Inflammatory arthritis | 192 | 1.11 (0.83-1.50) | 0.489 |
| Neuropathic pain | 600 | 0.95 (0.79-1.14) | 0.591 |
| Osteoarthritis | 175 | 0.96 (0.71-1.31) | 0.809 |
| **Age (Years)** |  |  |  |
| 18-30 | 416 | Ref. | - |
| 31-40 | 1,007 | 0.95 (0.75-1.19) | 0.655 |
| 41-50 | 1,027 | 0.98 (0.78-1.23) | 0.868 |
| 51-60 | 728 | 1.12 (0.88-1.43) | 0.356 |
| 61+ | 566 | 1.06 (0.82-1.37) | 0.640 |
| **Sex** |  |  |  |
| Male | 2,172 | Ref. | - |
| Female | 1,572 | 1.08 (0.94-1.23) | 0.276 |
| **BMI (kg/m2)** |  |  |  |
| 20-24.99 | 1,093 | Ref. | - |
| <20 | 296 | 1.07 (0.83-1.39) | 0.606 |
| 25-29.99 | 1,165 | 0.88 (0.75-1.04) | 0.145 |
| 30-34.99 | 551 | 1.06 (0.86-1.30) | 0.599 |
| 35+ | 455 | 1.08 (0.86-1.34) | 0.506 |
| **Cannabis Status** |  |  |  |
| Never Consumed Cannabis | 1,002 | Ref. | - |
| Former Cannabis Consumer | 590 | 1.07 (0.87-1.31) | 0.510 |
| Current Cannabis Consumer | 2,152 | 1.03 (0.89-1.20) | 0.674 |

| **CBMPs at 24 Months** |  |  |  |
| --- | --- | --- | --- |
| Oil | 879 | Ref. | - |
| Dried flower/flos | 801 | 1.09 (0.90-1.32) | 0.399 |
| Dried flower/flos, Oil | 1,748 | 1.08 (0.92-1.28) | 0.327 |
| Dried flower/flos, Pastille | 77 | 1.63 (1.01-2.69) | 0.048 |
| Dried flower/flos, Oil, Pastille | 68 | 0.88 (0.54-1.45) | 0.619 |
| Other | 89 | 0.99 (0.64-1.53) | 0.954 |
| None | 82 | 0.88 (0.56-1.39) | 0.587 |
| **Daily CBD Dose (mg/day) at 24 Months** |  |  |  |
| Min to Q1 | 962 | Ref. | - |
| Q1 to Median | 914 | 1.00 (0.83-1.20) | 0.983 |
| Median to Q3 | 932 | 0.94 (0.78-1.12) | 0.470 |
| Q3 to Max | 936 | 0.96 (0.80-1.15) | 0.653 |
| **Daily THC Dose (mg/day) at 24 Months** |  |  |  |
| Min to Q1 | 936 | Ref. | - |
| Q1 to Median | 942 | 1.18 (0.98-1.42) | 0.073 |
| Median to Q3 | 949 | 1.13 (0.94-1.36) | 0.180 |
| Q3 to Max | 917 | 1.10 (0.91-1.32) | 0.325 |
| **SQS at Baseline** |  |  |  |
| 7-10 | 558 | Ref. | - |
| 4-6 | 1,212 | 1.34 (1.10-1.64) | 0.005 |
| 0-3 | 1,974 | 2.41 (1.99-2.93) | <0.001 |
| **GAD-7 at Baseline** |  |  |  |
| <10 | 2,245 | Ref. | - |
| 10-14 | 683 | 1.38 (1.16-1.64) | <0.001 |
| 15+ | 816 | 1.66 (1.41-1.96) | <0.001 |
| **Prescribed Opioids at Baseline** |  |  |  |
| No | 2,300 | Ref. | - |
| Yes | 1,444 | 1.16 (1.01-1.32) | 0.030 |
| **Prescribed Gabapentinoids at Baseline** |  |  |  |
| No | 3,020 | Ref. | - |
| Yes | 724 | 1.25 (1.06-1.47) | 0.009 |

**Supplementary Table 39. Multivariable Logistic Regression for a MCID in BPI-SF Pain Severity at 24 Months.**

*BMI – body mass index; BPI-SF – Brief Pain Inventory Short Form; CBD – cannabidiol; CBMPs – cannabis-based medicinal products; CI – confidence interval; GAD-7 – Generalised Anxiety Disorder-7; Max – maximum; MCID – minimal clinically important difference; Min – minimum; OR – odds ratio; Q1 – quartile 1; Q3 – quartile 3; SQS – Single-Item Sleep Quality Scale; THC – (-)-trans-Δ9-tetrahydrocannabinol.*

| **Variable** | **n** | **OR (95% CI)** | **p-value** |
| --- | --- | --- | --- |
| **Primary Diagnosis** |  |  |  |
| Chronic Non-Cancer Pain | 2,153 | Ref. | - |
| Cancer pain | 110 | 0.76 (0.51-1.13) | 0.178 |
| Complex regional pain syndrome | 42 | 1.59 (0.84-3.18) | 0.168 |
| Endometriosis | 97 | 0.99 (0.64-1.53) | 0.958 |
| Hypermobility | 228 | 0.84 (0.62-1.12) | 0.228 |
| Inflammatory arthritis | 187 | 1.15 (0.85-1.58) | 0.369 |
| Neuropathic pain | 576 | 0.98 (0.81-1.19) | 0.872 |
| Osteoarthritis | 167 | 0.96 (0.69-1.34) | 0.804 |
| **Age (Years)** |  |  |  |
| 18-30 | 393 | Ref. | - |
| 31-40 | 969 | 0.95 (0.74-1.21) | 0.658 |
| 41-50 | 990 | 0.94 (0.74-1.20) | 0.635 |
| 51-60 | 697 | 1.06 (0.81-1.38) | 0.669 |
| 61+ | 511 | 1.20 (0.90-1.62) | 0.217 |
| **Sex** |  |  |  |
| Male | 2,077 | Ref. | - |
| Female | 1,483 | 1.04 (0.89-1.22) | 0.609 |
| **BMI (kg/m2)** |  |  |  |
| 20-24.99 | 1,093 | Ref. | - |
| <20 | 296 | 1.06 (0.81-1.39) | 0.663 |
| 25-29.99 | 1,165 | 0.87 (0.73-1.03) | 0.100 |
| 30-34.99 | 551 | 0.97 (0.79-1.21) | 0.812 |
| 35+ | 455 | 0.93 (0.74-1.17) | 0.548 |
| **Cannabis Status** |  |  |  |
| Never Consumed Cannabis | 923 | Ref. | - |
| Former Cannabis Consumer | 571 | 1.05 (0.84-1.33) | 0.659 |
| Current Cannabis Consumer | 2,066 | 1.03 (0.84-1.27) | 0.755 |

| **CBMPs at 24 Months** |  |  |  |
| --- | --- | --- | --- |
| Oil | 821 | Ref. | - |
| Dried flower/flos | 777 | 0.87 (0.53-1.42) | 0.576 |
| Dried flower/flos, Oil | 1,667 | 0.81 (0.50-1.30) | 0.387 |
| Dried flower/flos, Pastille | 76 | 1.15 (0.59-2.28) | 0.676 |
| Dried flower/flos, Oil, Pastille | 68 | 0.70 (0.35-1.38) | 0.307 |
| Other | 84 | 0.85 (0.49-1.47) | 0.569 |
| None | 67 | 0.74 (0.43-1.25) | 0.261 |
| **Daily CBD Dose (mg/day) at 24 Months** |  |  |  |
| Min to Q1 | 898 | Ref. | - |
| Q1 to Median | 863 | 0.98 (0.79-1.22) | 0.872 |
| Median to Q3 | 888 | 0.95 (0.77-1.18) | 0.655 |
| Q3 to Max | 911 | 0.94 (0.76-1.15) | 0.533 |
| **Daily THC Dose (mg/day) at 24 Months** |  |  |  |
| Min to Q1 | 863 | Ref. | - |
| Q1 to Median | 879 | 1.40 (0.88-2.24) | 0.159 |
| Median to Q3 | 916 | 1.31 (0.80-2.17) | 0.282 |
| Q3 to Max | 902 | 1.31 (0.79-2.17) | 0.299 |
| **SQS at Baseline** |  |  |  |
| 7-10 | 533 | Ref. | - |
| 4-6 | 1,155 | 1.33 (1.08-1.65) | 0.008 |
| 0-3 | 1,872 | 2.24 (1.82-2.76) | <0.001 |
| **GAD-7 at Baseline** |  |  |  |
| <10 | 2,139 | Ref. | - |
| 10-14 | 649 | 1.18 (0.98-1.42) | 0.078 |
| 15+ | 772 | 1.30 (1.09-1.56) | 0.004 |
| **Prescribed Opioids at Baseline** |  |  |  |
| No | 2,177 | Ref. | - |
| Yes | 1,383 | 1.04 (0.90-1.20) | 0.618 |
| **Prescribed Gabapentinoids at Baseline** |  |  |  |
| No | 2,876 | Ref. | - |
| Yes | 684 | 1.15 (0.96-1.38) | 0.123 |

**Supplementary Table 40. Univariable Logistic Regression for a MCID in BPI-SF Pain Interference at 24 Months.**

*BMI – body mass index; BPI-SF – Brief Pain Inventory Short Form; CBD – cannabidiol; CBMPs – cannabis-based medicinal products; CI – confidence interval; GAD-7 – Generalised Anxiety Disorder-7; Max – maximum; MCID – minimal clinically important difference; Min – minimum; OR – odds ratio; Q1 – quartile 1; Q3 – quartile 3; SQS – Single-Item Sleep Quality Scale; THC – (-)-trans-Δ9-tetrahydrocannabinol.*

| **Variable** | **n** | **OR (95% CI)** | **p-value** |
| --- | --- | --- | --- |
| **Primary Diagnosis** |  |  |  |
| Chronic Non-Cancer Pain | 2,276 | Ref. | - |
| Cancer pain | 116 | 0.54 (0.37-0.78) | 0.001 |
| Complex regional pain syndrome | 44 | 1.38 (0.73-2.74) | 0.335 |
| Endometriosis | 101 | 0.85 (0.57-1.28) | 0.421 |
| Hypermobility | 240 | 1.27 (0.96-1.70) | 0.099 |
| Inflammatory arthritis | 192 | 1.16 (0.85-1.59) | 0.360 |
| Neuropathic pain | 600 | 0.87 (0.72-1.04) | 0.131 |
| Osteoarthritis | 175 | 0.93 (0.68-1.28) | 0.664 |
| **Age (Years)** |  |  |  |
| 18-30 | 416 | Ref. | - |
| 31-40 | 1,007 | 0.94 (0.74-1.19) | 0.605 |
| 41-50 | 1,027 | 0.76 (0.60-0.97) | 0.027 |
| 51-60 | 728 | 0.78 (0.61-1.00) | 0.056 |
| 61+ | 566 | 0.73 (0.56-0.95) | 0.019 |
| **Sex** |  |  |  |
| Male | 2,172 | Ref. | - |
| Female | 1,572 | 1.14 (0.99-1.30) | 0.062 |
| **BMI (kg/m2)** |  |  |  |
| 20-24.99 | 1,093 | Ref. | - |
| <20 | 296 | 1.03 (0.80-1.35) | 0.802 |
| 25-29.99 | 1,165 | 1.00 (0.84-1.18) | 0.998 |
| 30-34.99 | 551 | 1.27 (1.03-1.58) | 0.027 |
| 35+ | 455 | 1.29 (1.02-1.62) | 0.032 |
| **Cannabis Status** |  |  |  |
| Never Consumed Cannabis | 1,002 | Ref. | - |
| Former Cannabis Consumer | 590 | 1.30 (1.05-1.60) | 0.015 |
| Current Cannabis Consumer | 2,152 | 1.28 (1.10-1.50) | 0.001 |

| **CBMPs at 24 Months** |  |  |  |
| --- | --- | --- | --- |
| Oil | 879 | Ref. | - |
| Dried flower/flos | 801 | 1.20 (0.99-1.47) | 0.066 |
| Dried flower/flos, Oil | 1,748 | 1.12 (0.95-1.33) | 0.171 |
| Dried flower/flos, Pastille | 77 | 2.02 (1.20-3.53) | 0.010 |
| Dried flower/flos, Oil, Pastille | 68 | 1.71 (1.00-3.01) | 0.055 |
| Other | 89 | 0.85 (0.55-1.32) | 0.463 |
| None | 82 | 0.89 (0.56-1.41) | 0.613 |
| **Daily CBD Dose (mg/day) at 24 Months** |  |  |  |
| Min to Q1 | 962 | Ref. | - |
| Q1 to Median | 914 | 1.02 (0.84-1.23) | 0.860 |
| Median to Q3 | 932 | 0.85 (0.71-1.02) | 0.085 |
| Q3 to Max | 936 | 0.98 (0.82-1.19) | 0.868 |
| **Daily THC Dose (mg/day) at 24 Months** |  |  |  |
| Min to Q1 | 936 | Ref. | - |
| Q1 to Median | 942 | 1.14 (0.95-1.38) | 0.152 |
| Median to Q3 | 949 | 1.28 (1.06-1.54) | 0.009 |
| Q3 to Max | 917 | 1.32 (1.10-1.60) | 0.004 |
| **SQS at Baseline** |  |  |  |
| 7-10 | 558 | Ref. | - |
| 4-6 | 1,212 | 1.44 (1.18-1.76) | <0.001 |
| 0-3 | 1,974 | 2.54 (2.10-3.08) | <0.001 |
| **GAD-7 at Baseline** |  |  |  |
| <10 | 2,245 | Ref. | - |
| 10-14 | 683 | 1.67 (1.39-2.01) | <0.001 |
| 15+ | 816 | 1.99 (1.67-2.37) | <0.001 |
| **Prescribed Opioids at Baseline** |  |  |  |
| No | 2,300 | Ref. | - |
| Yes | 1,444 | 1.22 (1.07-1.40) | 0.004 |
| **Prescribed Gabapentinoids at Baseline** |  |  |  |
| No | 3,020 | Ref. | - |
| Yes | 724 | 1.15 (0.97-1.36) | 0.112 |

**Supplementary Table 41. Multivariable Logistic Regression for a MCID in BPI-SF Pain Interference at 24 Months.**

*BMI – body mass index; BPI-SF – Brief Pain Inventory Short Form; CBD – cannabidiol; CBMPs – cannabis-based medicinal products; CI – confidence interval; GAD-7 – Generalised Anxiety Disorder-7; Max – maximum; MCID – minimal clinically important difference; Min – minimum; OR – odds ratio; Q1 – quartile 1; Q3 – quartile 3; SQS – Single-Item Sleep Quality Scale; THC – (-)-trans-Δ9-tetrahydrocannabinol.*

| **Variable** | **n** | **OR (95% CI)** | **p-value** |
| --- | --- | --- | --- |
| **Primary Diagnosis** |  |  |  |
| Chronic Non-Cancer Pain | 2,153 | Ref. | - |
| Cancer pain | 110 | 0.59 (0.39-0.89) | 0.011 |
| Complex regional pain syndrome | 42 | 1.22 (0.63-2.49) | 0.565 |
| Endometriosis | 97 | 0.87 (0.56-1.36) | 0.526 |
| Hypermobility | 228 | 1.21 (0.89-1.67) | 0.230 |
| Inflammatory arthritis | 187 | 1.16 (0.84-1.60) | 0.382 |
| Neuropathic pain | 576 | 0.96 (0.79-1.17) | 0.690 |
| Osteoarthritis | 167 | 1.01 (0.72-1.43) | 0.947 |
| **Age (Years)** |  |  |  |
| 18-30 | 393 | Ref. | - |
| 31-40 | 969 | 0.92 (0.71-1.20) | 0.554 |
| 41-50 | 990 | 0.73 (0.56-0.94) | 0.017 |
| 51-60 | 697 | 0.75 (0.57-0.99) | 0.044 |
| 61+ | 511 | 0.87 (0.64-1.18) | 0.372 |
| **Sex** |  |  |  |
| Male | 2,077 | Ref. | - |
| Female | 1,483 | 1.13 (0.96-1.33) | 0.136 |
| **BMI (kg/m2)** |  |  |  |
| 20-24.99 | 1,093 | Ref. | - |
| <20 | 296 | 0.99 (0.76-1.31) | 0.962 |
| 25-29.99 | 1,165 | 1.02 (0.86-1.22) | 0.785 |
| 30-34.99 | 551 | 1.24 (0.99-1.54) | 0.062 |
| 35+ | 455 | 1.12 (0.88-1.43) | 0.344 |
| **Cannabis Status** |  |  |  |
| Never Consumed Cannabis | 923 | Ref. | - |
| Former Cannabis Consumer | 571 | 1.20 (0.94-1.52) | 0.137 |
| Current Cannabis Consumer | 2,066 | 1.23 (1.00-1.52) | 0.052 |

| **CBMPs at 24 Months** |  |  |  |
| --- | --- | --- | --- |
| Oil | 821 | Ref. | - |
| Dried flower/flos | 777 | 0.70 (0.42-1.17) | 0.180 |
| Dried flower/flos, Oil | 1,667 | 0.65 (0.40-1.06) | 0.091 |
| Dried flower/flos, Pastille | 76 | 1.01 (0.49-2.11) | 0.974 |
| Dried flower/flos, Oil, Pastille | 68 | 1.06 (0.51-2.23) | 0.877 |
| Other | 84 | 0.66 (0.37-1.15) | 0.142 |
| None | 67 | 0.67 (0.39-1.16) | 0.153 |
| **Daily CBD Dose (mg/day) at 24 Months** |  |  |  |
| Min to Q1 | 898 | Ref. | - |
| Q1 to Median | 863 | 0.92 (0.74-1.16) | 0.488 |
| Median to Q3 | 888 | 0.82 (0.66-1.03) | 0.088 |
| Q3 to Max | 911 | 0.89 (0.72-1.11) | 0.308 |
| **Daily THC Dose (mg/day) at 24 Months** |  |  |  |
| Min to Q1 | 863 | Ref. | - |
| Q1 to Median | 879 | 1.52 (0.94-2.50) | 0.089 |
| Median to Q3 | 916 | 1.62 (0.97-2.73) | 0.068 |
| Q3 to Max | 902 | 1.77 (1.05-3.01) | 0.033 |
| **SQS at Baseline** |  |  |  |
| 7-10 | 533 | Ref. | - |
| 4-6 | 1,155 | 1.35 (1.10-1.67) | 0.005 |
| 0-3 | 1,872 | 2.13 (1.73-2.62) | <0.001 |
| **GAD-7 at Baseline** |  |  |  |
| <10 | 2,139 | Ref. | - |
| 10-14 | 649 | 1.41 (1.16-1.71) | <0.001 |
| 15+ | 772 | 1.56 (1.29-1.89) | <0.001 |
| **Prescribed Opioids at Baseline** |  |  |  |
| No | 2,177 | Ref. | - |
| Yes | 1,383 | 1.11 (0.96-1.29) | 0.157 |
| **Prescribed Gabapentinoids at Baseline** |  |  |  |
| No | 2,876 | Ref. | - |
| Yes | 684 | 1.03 (0.85-1.24) | 0.782 |

**Supplementary Table 42. Univariable Logistic Regression for a MCID in SF-MPQ-2 Total at 24 Months.**

*BMI – body mass index; CBD – cannabidiol; CBMPs – cannabis-based medicinal products; CI – confidence interval; GAD-7 – Generalised Anxiety Disorder-7; Max – maximum; MCID – minimal clinically important difference; Min – minimum; OR – odds ratio; Q1 – quartile 1; Q3 – quartile 3; SF-MPQ-2 – Short-Form McGill Pain Questionnaire-2; SQS – Single-Item Sleep Quality Scale; THC – (-)-trans-Δ9-tetrahydrocannabinol.*

| **Variable** | **n** | **OR (95% CI)** | **p-value** |
| --- | --- | --- | --- |
| **Primary Diagnosis** |  |  |  |
| Chronic Non-Cancer Pain | 2,276 | Ref. | - |
| Cancer pain | 116 | 0.80 (0.55-1.17) | 0.248 |
| Complex regional pain syndrome | 44 | 2.07 (1.09-4.20) | 0.033 |
| Endometriosis | 101 | 1.09 (0.73-1.64) | 0.679 |
| Hypermobility | 240 | 1.09 (0.83-1.42) | 0.551 |
| Inflammatory arthritis | 192 | 0.84 (0.63-1.13) | 0.256 |
| Neuropathic pain | 600 | 0.85 (0.71-1.01) | 0.068 |
| Osteoarthritis | 175 | 0.82 (0.60-1.12) | 0.209 |
| **Age (Years)** |  |  |  |
| 18-30 | 416 | Ref. | - |
| 31-40 | 1,007 | 0.87 (0.69-1.09) | 0.228 |
| 41-50 | 1,027 | 0.87 (0.69-1.09) | 0.226 |
| 51-60 | 728 | 0.88 (0.69-1.13) | 0.326 |
| 61+ | 566 | 0.77 (0.59-0.99) | 0.040 |
| **Sex** |  |  |  |
| Male | 2,172 | Ref. | - |
| Female | 1,572 | 1.16 (1.02-1.32) | 0.029 |
| **BMI (kg/m2)** |  |  |  |
| 20-24.99 | 1,093 | Ref. | - |
| <20 | 296 | 1.31 (1.01-1.70) | 0.042 |
| 25-29.99 | 1,165 | 1.04 (0.88-1.22) | 0.661 |
| 30-34.99 | 551 | 1.22 (0.99-1.50) | 0.059 |
| 35+ | 455 | 1.21 (0.97-1.51) | 0.095 |
| **Cannabis Status** |  |  |  |
| Never Consumed Cannabis | 1,002 | Ref. | - |
| Former Cannabis Consumer | 590 | 1.25 (1.02-1.54) | 0.031 |
| Current Cannabis Consumer | 2,152 | 1.26 (1.09-1.47) | 0.002 |

| **CBMPs at 24 Months** |  |  |  |
| --- | --- | --- | --- |
| Oil | 879 | Ref. | - |
| Dried flower/flos | 801 | 1.24 (1.02-1.50) | 0.029 |
| Dried flower/flos, Oil | 1,748 | 1.18 (1.01-1.39) | 0.042 |
| Dried flower/flos, Pastille | 77 | 1.06 (0.66-1.69) | 0.818 |
| Dried flower/flos, Oil, Pastille | 68 | 1.60 (0.97-2.69) | 0.073 |
| Other | 89 | 1.30 (0.84-2.04) | 0.239 |
| None | 82 | 1.13 (0.72-1.79) | 0.603 |
| **Daily CBD Dose (mg/day) at 24 Months** |  |  |  |
| Min to Q1 | 962 | Ref. | - |
| Q1 to Median | 914 | 1.00 (0.84-1.20) | 0.979 |
| Median to Q3 | 932 | 1.03 (0.86-1.23) | 0.757 |
| Q3 to Max | 936 | 0.99 (0.83-1.18) | 0.902 |
| **Daily THC Dose (mg/day) at 24 Months** |  |  |  |
| Min to Q1 | 936 | Ref. | - |
| Q1 to Median | 942 | 1.14 (0.95-1.36) | 0.168 |
| Median to Q3 | 949 | 1.28 (1.06-1.53) | 0.009 |
| Q3 to Max | 917 | 1.19 (0.99-1.43) | 0.060 |
| **SQS at Baseline** |  |  |  |
| 7-10 | 558 | Ref. | - |
| 4-6 | 1,212 | 1.52 (1.24-1.87) | <0.001 |
| 0-3 | 1,974 | 2.74 (2.26-3.33) | <0.001 |
| **GAD-7 at Baseline** |  |  |  |
| <10 | 2,245 | Ref. | - |
| 10-14 | 683 | 1.77 (1.49-2.11) | <0.001 |
| 15+ | 816 | 2.04 (1.72-2.41) | <0.001 |
| **Prescribed Opioids at Baseline** |  |  |  |
| No | 2,300 | Ref. | - |
| Yes | 1,444 | 1.11 (0.98-1.27) | 0.109 |
| **Prescribed Gabapentinoids at Baseline** |  |  |  |
| No | 3,020 | Ref. | - |
| Yes | 724 | 1.24 (1.05-1.46) | 0.011 |

**Supplementary Table 43. Multivariable Logistic Regression for a MCID in SF-MPQ-2 Total at 24 Months.**

*BMI – body mass index; CBD – cannabidiol; CBMPs – cannabis-based medicinal products; CI – confidence interval; GAD-7 – Generalised Anxiety Disorder-7; Max – maximum; MCID – minimal clinically important difference; Min – minimum; OR – odds ratio; Q1 – quartile 1; Q3 – quartile 3; SF-MPQ-2 – Short-Form McGill Pain Questionnaire-2; SQS – Single-Item Sleep Quality Scale; THC – (-)-trans-Δ9-tetrahydrocannabinol.*

| **Variable** | **n** | **OR (95% CI)** | **p-value** |
| --- | --- | --- | --- |
| **Primary Diagnosis** |  |  |  |
| Chronic Non-Cancer Pain | 2,153 | Ref. | - |
| Cancer pain | 110 | 0.92 (0.61-1.38) | 0.682 |
| Complex regional pain syndrome | 42 | 1.91 (0.98-3.97) | 0.068 |
| Endometriosis | 97 | 1.24 (0.80-1.95) | 0.347 |
| Hypermobility | 228 | 1.00 (0.74-1.35) | 0.995 |
| Inflammatory arthritis | 187 | 0.85 (0.63-1.17) | 0.321 |
| Neuropathic pain | 576 | 0.89 (0.73-1.07) | 0.216 |
| Osteoarthritis | 167 | 0.89 (0.64-1.24) | 0.476 |
| **Age (Years)** |  |  |  |
| 18-30 | 393 | Ref. | - |
| 31-40 | 969 | 0.87 (0.68-1.11) | 0.262 |
| 41-50 | 990 | 0.86 (0.67-1.10) | 0.240 |
| 51-60 | 697 | 0.88 (0.67-1.14) | 0.328 |
| 61+ | 511 | 0.94 (0.70-1.26) | 0.680 |
| **Sex** |  |  |  |
| Male | 2,077 | Ref. | - |
| Female | 1,483 | 1.15 (0.98-1.35) | 0.079 |
| **BMI (kg/m2)** |  |  |  |
| 20-24.99 | 1,093 | Ref. | - |
| <20 | 296 | 1.26 (0.96-1.66) | 0.093 |
| 25-29.99 | 1,165 | 1.06 (0.89-1.25) | 0.541 |
| 30-34.99 | 551 | 1.18 (0.95-1.46) | 0.128 |
| 35+ | 455 | 1.07 (0.85-1.36) | 0.544 |
| **Cannabis Status** |  |  |  |
| Never Consumed Cannabis | 923 | Ref. | - |
| Former Cannabis Consumer | 571 | 1.23 (0.97-1.55) | 0.083 |
| Current Cannabis Consumer | 2,066 | 1.30 (1.06-1.60) | 0.012 |

| **CBMPs at 24 Months** |  |  |  |
| --- | --- | --- | --- |
| Oil | 821 | Ref. | - |
| Dried flower/flos | 777 | 1.16 (0.71-1.90) | 0.547 |
| Dried flower/flos, Oil | 1,667 | 1.01 (0.63-1.61) | 0.964 |
| Dried flower/flos, Pastille | 76 | 0.76 (0.39-1.47) | 0.416 |
| Dried flower/flos, Oil, Pastille | 68 | 1.37 (0.69-2.77) | 0.370 |
| Other | 84 | 1.17 (0.68-2.04) | 0.574 |
| None | 67 | 1.09 (0.63-1.87) | 0.765 |
| **Daily CBD Dose (mg/day) at 24 Months** |  |  |  |
| Min to Q1 | 898 | Ref. | - |
| Q1 to Median | 863 | 1.02 (0.82-1.27) | 0.852 |
| Median to Q3 | 888 | 1.11 (0.89-1.37) | 0.365 |
| Q3 to Max | 911 | 0.98 (0.80-1.22) | 0.886 |
| **Daily THC Dose (mg/day) at 24 Months** |  |  |  |
| Min to Q1 | 863 | Ref. | - |
| Q1 to Median | 879 | 1.04 (0.65-1.65) | 0.880 |
| Median to Q3 | 916 | 1.12 (0.68-1.84) | 0.662 |
| Q3 to Max | 902 | 1.06 (0.64-1.75) | 0.826 |
| **SQS at Baseline** |  |  |  |
| 7-10 | 533 | Ref. | - |
| 4-6 | 1,155 | 1.47 (1.19-1.82) | <0.001 |
| 0-3 | 1,872 | 2.41 (1.96-2.97) | <0.001 |
| **GAD-7 at Baseline** |  |  |  |
| <10 | 2,139 | Ref. | - |
| 10-14 | 649 | 1.50 (1.25-1.81) | <0.001 |
| 15+ | 772 | 1.58 (1.32-1.90) | <0.001 |
| **Prescribed Opioids at Baseline** |  |  |  |
| No | 2,177 | Ref. | - |
| Yes | 1,383 | 0.99 (0.86-1.15) | 0.931 |
| **Prescribed Gabapentinoids at Baseline** |  |  |  |
| No | 2,876 | Ref. | - |
| Yes | 684 | 1.13 (0.94-1.36) | 0.181 |

**Supplementary Table 44. Univariable Logistic Regression for a MCID in Pain VAS at 24 Months.**

*BMI – body mass index; CBD – cannabidiol; CBMPs – cannabis-based medicinal products; CI – confidence interval; GAD-7 – Generalised Anxiety Disorder-7; Max – maximum; MCID – minimal clinically important difference; Min – minimum; OR – odds ratio; Q1 – quartile 1; Q3 – quartile 3; SQS – Single-Item Sleep Quality Scale; THC – (-)-trans-Δ9-tetrahydrocannabinol; VAS – visual analogue scale.*

| **Variable** | **n** | **OR (95% CI)** | **p-value** |
| --- | --- | --- | --- |
| **Primary Diagnosis** |  |  |  |
| Chronic Non-Cancer Pain | 2,276 | Ref. | - |
| Cancer pain | 116 | 0.58 (0.40-0.84) | 0.004 |
| Complex regional pain syndrome | 44 | 1.47 (0.78-2.92) | 0.248 |
| Endometriosis | 101 | 0.83 (0.56-1.25) | 0.370 |
| Hypermobility | 240 | 0.91 (0.69-1.19) | 0.490 |
| Inflammatory arthritis | 192 | 1.00 (0.74-1.37) | 0.975 |
| Neuropathic pain | 600 | 0.92 (0.77-1.11) | 0.404 |
| Osteoarthritis | 175 | 1.15 (0.84-1.60) | 0.389 |
| **Age (Years)** |  |  |  |
| 18-30 | 416 | Ref. | - |
| 31-40 | 1,007 | 0.98 (0.78-1.24) | 0.869 |
| 41-50 | 1,027 | 1.01 (0.80-1.28) | 0.911 |
| 51-60 | 728 | 1.23 (0.96-1.57) | 0.103 |
| 61+ | 566 | 1.11 (0.86-1.44) | 0.425 |
| **Sex** |  |  |  |
| Male | 2,172 | Ref. | - |
| Female | 1,572 | 1.00 (0.88-1.15) | 0.951 |
| **BMI (kg/m2)** |  |  |  |
| 20-24.99 | 1,093 | Ref. | - |
| <20 | 296 | 1.26 (0.97-1.65) | 0.085 |
| 25-29.99 | 1,165 | 1.12 (0.94-1.32) | 0.200 |
| 30-34.99 | 551 | 1.11 (0.90-1.37) | 0.327 |
| 35+ | 455 | 1.28 (1.02-1.60) | 0.035 |
| **Cannabis Status** |  |  |  |
| Never Consumed Cannabis | 1,002 | Ref. | - |
| Former Cannabis Consumer | 590 | 1.03 (0.84-1.27) | 0.769 |
| Current Cannabis Consumer | 2,152 | 1.15 (0.99-1.34) | 0.070 |

| **CBMPs at 24 Months** |  |  |  |
| --- | --- | --- | --- |
| Oil | 879 | Ref. | - |
| Dried flower/flos | 801 | 1.26 (1.04-1.54) | 0.020 |
| Dried flower/flos, Oil | 1,748 | 1.16 (0.98-1.36) | 0.085 |
| Dried flower/flos, Pastille | 77 | 1.41 (0.87-2.34) | 0.168 |
| Dried flower/flos, Oil, Pastille | 68 | 0.86 (0.52-1.42) | 0.550 |
| Other | 89 | 1.35 (0.86-2.15) | 0.200 |
| None | 82 | 0.88 (0.56-1.39) | 0.568 |
| **Daily CBD Dose (mg/day) at 24 Months** |  |  |  |
| Min to Q1 | 962 | Ref. | - |
| Q1 to Median | 914 | 0.91 (0.76-1.10) | 0.324 |
| Median to Q3 | 932 | 0.93 (0.77-1.12) | 0.431 |
| Q3 to Max | 936 | 1.07 (0.89-1.28) | 0.499 |
| **Daily THC Dose (mg/day) at 24 Months** |  |  |  |
| Min to Q1 | 936 | Ref. | - |
| Q1 to Median | 942 | 1.24 (1.03-1.50) | 0.020 |
| Median to Q3 | 949 | 1.36 (1.13-1.64) | 0.001 |
| Q3 to Max | 917 | 1.28 (1.06-1.54) | 0.010 |
| **SQS at Baseline** |  |  |  |
| 7-10 | 558 | Ref. | - |
| 4-6 | 1,212 | 1.36 (1.11-1.66) | 0.003 |
| 0-3 | 1,974 | 2.29 (1.89-2.77) | <0.001 |
| **GAD-7 at Baseline** |  |  |  |
| <10 | 2,245 | Ref. | - |
| 10-14 | 683 | 1.46 (1.23-1.75) | <0.001 |
| 15+ | 816 | 1.57 (1.33-1.87) | <0.001 |
| **Prescribed Opioids at Baseline** |  |  |  |
| No | 2,300 | Ref. | - |
| Yes | 1,444 | 1.09 (0.95-1.24) | 0.237 |
| **Prescribed Gabapentinoids at Baseline** |  |  |  |
| No | 3,020 | Ref. | - |
| Yes | 724 | 1.22 (1.03-1.45) | 0.020 |

**Supplementary Table 45. Multivariable Logistic Regression for a MCID in Pain VAS at 24 Months.**

*BMI – body mass index; CBD – cannabidiol; CBMPs – cannabis-based medicinal products; CI – confidence interval; GAD-7 – Generalised Anxiety Disorder-7; Max – maximum; MCID – minimal clinically important difference; Min – minimum; OR – odds ratio; Q1 – quartile 1; Q3 – quartile 3; SQS – Single-Item Sleep Quality Scale; THC – (-)-trans-Δ9-tetrahydrocannabinol; VAS – visual analogue scale.*

| **Variable** | **n** | **OR (95% CI)** | **p-value** |
| --- | --- | --- | --- |
| **Primary Diagnosis** |  |  |  |
| Chronic Non-Cancer Pain | 2,153 | Ref. | - |
| Cancer pain | 110 | 0.63 (0.42-0.95) | 0.027 |
| Complex regional pain syndrome | 42 | 1.56 (0.80-3.24) | 0.207 |
| Endometriosis | 97 | 1.01 (0.65-1.57) | 0.971 |
| Hypermobility | 228 | 0.95 (0.70-1.27) | 0.709 |
| Inflammatory arthritis | 187 | 1.03 (0.75-1.41) | 0.853 |
| Neuropathic pain | 576 | 0.99 (0.82-1.21) | 0.945 |
| Osteoarthritis | 167 | 1.19 (0.84-1.69) | 0.327 |
| **Age (Years)** |  |  |  |
| 18-30 | 393 | Ref. | - |
| 31-40 | 969 | 0.93 (0.73-1.20) | 0.586 |
| 41-50 | 990 | 0.96 (0.75-1.24) | 0.774 |
| 51-60 | 697 | 1.17 (0.89-1.53) | 0.258 |
| 61+ | 511 | 1.24 (0.92-1.68) | 0.155 |
| **Sex** |  |  |  |
| Male | 2,077 | Ref. | - |
| Female | 1,483 | 0.97 (0.83-1.14) | 0.739 |
| **BMI (kg/m2)** |  |  |  |
| 20-24.99 | 1,093 | Ref. | - |
| <20 | 296 | 1.28 (0.97-1.68) | 0.083 |
| 25-29.99 | 1,165 | 1.11 (0.93-1.32) | 0.252 |
| 30-34.99 | 551 | 1.05 (0.85-1.30) | 0.655 |
| 35+ | 455 | 1.19 (0.94-1.50) | 0.158 |
| **Cannabis Status** |  |  |  |
| Never Consumed Cannabis | 923 | Ref. | - |
| Former Cannabis Consumer | 571 | 0.98 (0.78-1.24) | 0.872 |
| Current Cannabis Consumer | 2,066 | 1.13 (0.92-1.39) | 0.245 |

| **CBMPs at 24 Months** |  |  |  |
| --- | --- | --- | --- |
| Oil | 821 | Ref. | - |
| Dried flower/flos | 777 | 0.53 (0.31-0.90) | 0.020 |
| Dried flower/flos, Oil | 1,667 | 0.50 (0.30-0.83) | 0.008 |
| Dried flower/flos, Pastille | 76 | 0.54 (0.27-1.10) | 0.090 |
| Dried flower/flos, Oil, Pastille | 68 | 0.37 (0.18-0.74) | 0.005 |
| Other | 84 | 0.76 (0.43-1.35) | 0.352 |
| None | 67 | 0.73 (0.43-1.25) | 0.252 |
| **Daily CBD Dose (mg/day) at 24 Months** |  |  |  |
| Min to Q1 | 898 | Ref. | - |
| Q1 to Median | 863 | 0.88 (0.71-1.10) | 0.268 |
| Median to Q3 | 888 | 0.92 (0.74-1.14) | 0.430 |
| Q3 to Max | 911 | 1.01 (0.81-1.25) | 0.959 |
| **Daily THC Dose (mg/day) at 24 Months** |  |  |  |
| Min to Q1 | 863 | Ref. | - |
| Q1 to Median | 879 | 2.25 (1.37-3.79) | 0.002 |
| Median to Q3 | 916 | 2.55 (1.51-4.40) | <0.001 |
| Q3 to Max | 902 | 2.42 (1.42-4.20) | 0.001 |
| **SQS at Baseline** |  |  |  |
| 7-10 | 533 | Ref. | - |
| 4-6 | 1,155 | 1.34 (1.09-1.65) | 0.006 |
| 0-3 | 1,872 | 2.07 (1.69-2.55) | <0.001 |
| **GAD-7 at Baseline** |  |  |  |
| <10 | 2,139 | Ref. | - |
| 10-14 | 649 | 1.35 (1.12-1.64) | 0.002 |
| 15+ | 772 | 1.25 (1.04-1.51) | 0.018 |
| **Prescribed Opioids at Baseline** |  |  |  |
| No | 2,177 | Ref. | - |
| Yes | 1,383 | 0.96 (0.83-1.11) | 0.597 |
| **Prescribed Gabapentinoids at Baseline** |  |  |  |
| No | 2,876 | Ref. | - |
| Yes | 684 | 1.14 (0.95-1.37) | 0.167 |

**Supplementary Table 46. Overview of Cluster Associations**

Number (n) of participants per cluster, alongside mean Brief Pain Inventory Short Form (BPI-SF) pain severity score ± standard deviation (SD) at each recorded time period.

| **Cluster** | **Month** | **Mean ± SD** |
| --- | --- | --- |
| A (n=956) | Baseline | 5.85 ± 1.15 |
|  | 1 month | 5.30 ± 1.11 |
|  | 3 months | 5.12 ± 1.09 |
|  | 6 months | 5.42 ± 1.13 |
|  | 12 months | 5.27 ± 1.09 |
|  | 18 months | 5.93 ± 1.03 |
|  | 24 months | 5.79 ± 1.00 |
| B (n=946) | Baseline | 5.10 ± 1.33 |
|  | 1 month | 3.90 ± 1.21 |
|  | 3 months | 3.67 ± 1.19 |
|  | 6 months | 3.93 ± 1.21 |
|  | 12 months | 4.03 ± 1.21 |
|  | 18 months | 4.30 ± 1.21 |
|  | 24 months | 4.44 ± 1.23 |
| C (n=679) | Baseline | 7.72 ± 1.15 |
|  | 1 month | 7.07 ± 1.17 |
|  | 3 months | 6.81 ± 1.11 |
|  | 6 months | 6.78 ± 1.24 |
|  | 12 months | 6.49 ± 1.27 |
|  | 18 months | 6.63 ± 1.23 |
|  | 24 months | 6.30 ± 1.26 |
| D (n=675) | Baseline | 6.86 ± 1.19 |
|  | 1 month | 5.93 ± 1.16 |
|  | 3 months | 5.52 ± 1.26 |
|  | 6 months | 4.82 ± 1.23 |
|  | 12 months | 4.55 ± 1.33 |
|  | 18 months | 4.36 ± 1.15 |
|  | 24 months | 4.03 ± 1.17 |
| E (n=488) | Baseline | 4.26 ± 1.67 |
|  | 1 month | 2.81 ± 1.38 |
|  | 3 months | 2.51 ± 1.25 |
|  | 6 months | 2.20 ± 1.15 |
|  | 12 months | 2.23 ± 1.12 |
|  | 18 months | 2.49 ± 1.21 |
|  | 24 months | 2.57 ± 1.36 |

**Supplementary Table 47. Univariable Logistic Regression for Cluster Membership.**

p-values presented with Benjamini-Hochberg correction. BMI – body mass index; CBD – cannabidiol; CBMPs – cannabis-based medicinal products; CI – confidence interval; GAD-7 – Generalised Anxiety Disorder-7; Max – maximum; Min – minimum; OR – odds ratio; Q1 – quartile 1; Q3 – quartile 3; SQS – Single-Item Sleep Quality Scale; THC – (−)-trans-Δ9-tetrahydrocannabinol

*File name: Cluster_Univariable_Analysis.xlsx*

**Supplementary Table 48. Stepwise Multivariable Logistic Regression for Cluster Membership.**

p-values presented with Benjamini-Hochberg correction. BMI – body mass index; CBD – cannabidiol; CBMPs – cannabis-based medicinal products; CI – confidence interval; GAD-7 – Generalised Anxiety Disorder-7; Max – maximum; Min – minimum; OR – odds ratio; Q1 – quartile 1; Q3 – quartile 3; SQS – Single-Item Sleep Quality Scale; THC – (−)-trans-Δ9-tetrahydrocannabinol

*File name: Cluster_Stepwise_Multivariable_Analysis.xlsx*

**Supplementary Table 49. Multivariable Logistic Regression with Least Absolute Shrinkage and Selection Operator (LASSO) Regularisation for Cluster Membership.**

p-values presented with Benjamini-Hochberg correction. BMI – body mass index; CBD – cannabidiol; CBMPs – cannabis-based medicinal products; CI – confidence interval; GAD-7 – Generalised Anxiety Disorder-7; Max – maximum; Min – minimum; OR – odds ratio; Q1 – quartile 1; Q3 – quartile 3; SQS – Single-Item Sleep Quality Scale; THC – (−)-trans-Δ9-tetrahydrocannabinol

*File name: Cluster_LASSO_Multivariable_Analysis.xlsx*

**Supplementary Table 50. Cluster Trajectory Patterns and Associated Patient Characteristics**

Narrative synthesis of cluster trajectories, key patient characteristics significant on both stepwise and least absolute shrinkage and selection operator (LASSO) multivariable logistic regression, and clinical profiles. Brief Pain Inventory Short Form (BPI-SF) pain severity scores presented as mean ± standard deviation. *BMI – body mass index; CBD – cannabidiol; GAD-7 – Generalised Anxiety Disorder-7; Q1 – quartile 1; Q3 – quartile 3; SQS – Single-Item Sleep Quality Scale; THC – (−)-trans-Δ9-tetrahydrocannabinol.*

| **Trajectory Pattern** | **Cluster** | **Baseline BPI-SF Pain Severity** | **24-month BPI-SF Pain Severity** | **Change** | **Key Patient Characteristics** | **Clinical Profile** |
| --- | --- | --- | --- | --- | --- | --- |
| **Significant Improvement** | **C**  **(n=679)** | 7.72 ± 1.15 | 6.30 ± 1.26 | -1.41 | - Unemployed - Poor baseline sleep quality (SQS 0–3) - Severe baseline anxiety (GAD-7 ≥15) - Prescribed opioids at baseline - Prescribed gabapentinoids at baseline - Lower daily THC dose at 1 month | Patients with the most severe baseline pain showing gradual improvement over 24 months |
|  | **D**  **(n=675)** | 6.86 ± 1.19 | 4.03 ± 1.17 | -2.84 | - Unemployed - Poor baseline sleep quality (SQS 0–3) - Younger age (under 41 years) - Cannabis-naïve or current consumer (rather than ex-consumer) | Patients with severe baseline pain showing progressive, sustained improvement, the largest across clusters |
|  | **E**  **(n=488)** | 4.26 ± 1.67 | 2.57 ± 1.36 | -1.69 | - Cancer pain or neuropathic pain primary diagnosis - Male sex - Younger age (under 60 years) - Employed - Ex-smoker (rather than current smoker) - Good-to-excellent baseline sleep quality (SQS 7–10) - Low baseline anxiety (GAD-7 <10) - Not prescribed opioids at baseline | Patients with the mildest baseline pain achieving rapid improvement within one month, which is sustained |
| **Modest Improvement** | **B**  **(n=946)** | 5.10 ± 1.33 | 4.44 ± 1.23 | -0.66 | - Employed - Good-to-excellent baseline sleep quality (SQS 7–10) - Not prescribed gabapentinoids at baseline | Patients with moderate baseline pain achieving early improvement that partially attenuates by 24 months |
| **Initial Improvement with Decline** | **A**  **(n=956)** | 5.85 ± 1.15 | 5.79 ± 1.00 | -0.06 | - Prescribed opioids at baseline - Lower daily CBD dose at 1 month | Patients with moderate baseline pain showing initial improvement followed by return towards baseline |

**Supplementary Figure 1. Comparison of Model Performance between Stepwise Multivariable Logistic Regression and Multivariable Logistic Regression with Least Absolute Shrinkage and Selection Operator (LASSO) Regularisation**

Model performance assessed using the area under the curve (AUC).
